# HDAC3 Regulates Transcriptional Networks Governing Decidualization

**DOI:** 10.1096/fj.202600629R

**Published:** 2026-04-15

**Authors:** Loan Thi Kim Nguyen, Dinh Nam Tran, Shamsun Nahar, Tae Hoon Kim, Jae‐Wook Jeong, Jung‐Yoon Yoo

**Affiliations:** ^1^ Department of Obstetrics, Gynecology and Women's Health University of Missouri School of Medicine Columbia Missouri USA; ^2^ Department of Biomedical Laboratory Science Yonsei University Mirae Campus Wonju Republic of Korea

**Keywords:** decidualization, HDAC3, infertility, uterus

## Abstract

Decidualization is essential for embryo implantation and maintenance of pregnancy, during which quiescent endometrial stromal fibroblasts proliferate and differentiate into decidual stromal cells. Emerging evidence indicates that epigenetic regulators, including histone modifications, play critical roles in uterine receptivity, implantation, and stromal cell decidualization. Our previous study demonstrated that loss of histone deacetylase 3 (HDAC3) impairs endometrial receptivity and decidualization, resulting in female infertility. However, the genome‐wide transcriptomic alterations responsible for defective decidualization in loss of HDAC3 remain unclear. In this study, uterine‐specific *Hdac3* knockout (*Pgr* ^*cre/+*^ *Hdac3* ^*f/f*^; *Hdac3* ^*d/d*^) mice exhibited decidual defects following 3 days of artificial decidualization. RNA sequencing analysis of uteri from control and *Hdac3* ^*d/d*^ mice revealed widespread dysregulation of genes and pathways associated with decidualization. Pathway analysis identified significant alterations in RHOA, AMPK‐NOTCH1‐HEY1, and oxidative stress‐induced senescence signaling, implicating dysregulation of cytoskeletal remodeling, cellular metabolism, and oxidative stress responses in the HDAC3‐mediated decidual response. Notably, expression of *Limk1*, *Prkag1*, and *Cbx2* for key regulators of these pathways was significantly reduced in *Hdac3* ^*d/d*^ mice compared with controls. These findings demonstrate that HDAC3 is a key regulator of the transcriptional and signaling networks required for successful decidualization. Collectively, our study provides a comprehensive transcriptomic profile of HDAC3‐deficient uteri and uncovers key molecular mechanisms underlying impaired decidualization, thereby advancing our understanding of uterine function and pregnancy establishment.

## Graphical abstract

HDAC3 orchestrates transcriptional programs essential for uterine decidualization. Loss of HDAC3 disrupts cytoskeletal organization, metabolic signaling, and oxidative stress responses, impairing stromal cell differentiation and decidualization. These alterations compromise uterine receptivity and result in failed embryo implantation.

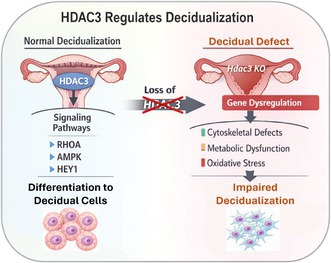

**Abbreviations**
AMPK: AMP‐activated protein kinase
ANOVA: analysis of variance
ART: assisted reproductive technology
BMP2: bone morphogenetic protein 2
CBX2: chromobox protein homolog 2
cDNA: complementary DNA
ChIP: chromatin immunoprecipitation
DAB: 33′‐diaminobenzidine
DAPI, 4′: 6‐diamidino‐2‐phenylindole
DEGs: differentially expressed genes
E2: estradiol
ECM: extracellular matrix
FASEB: Federation of American Societies for Experimental Biology
GD: gestational day
HDAC3: histone deacetylase 3
hESCs: human endometrial stromal cells
HEY1: hes‐related family bHLH transcription factor with YRPW motif 1
H‐score: histological score
IHC: immunohistochemistry
IPA: Ingenuity Pathway Analysis
KO: knockout
LIMK1: LIM domain kinase 1
NCoR: nuclear receptor corepressor
NGS: normal goat serum
P4: progesterone
PBS: phosphate‐buffered saline
PDZ: primary decidual zone
PGR: progesterone receptor
PRC1: Polycomb repressive complex 1
PRKAG1: protein kinase AMP‐activated non‐catalytic subunit gamma 1
qPCR: quantitative polymerase chain reaction
RHOA: Ras homolog family member A
RNA‐seq: RNA sequencing
ROCK: Rho‐associated protein kinase
RT‐qPCR: reverse transcription quantitative polymerase chain reaction
SDZ: secondary decidual zone
SEM: standard error of the mean
SMRT: silencing mediator for retinoid and thyroid hormone receptors
STAR: Spliced Transcripts Alignment to a Reference
WNT4: wingless‐type MMTV integration site family member 4

## 1 Introduction

Infertility is a rapidly increasing global health problem and ranks fifth among the most serious disabilities affecting women worldwide. Multiple factors contribute to infertility, including poor embryo quality and endometrial dysfunction; two of the most common causes of pregnancy failure [1, 2]. Despite remarkable advances in assisted reproductive technologies (ART), more than 75% of pregnancy loss is attributed to implantation failure of the fertilized embryo into the uterus [3, 4]. This highlights the critical importance of the decidualization process in establishing a receptive endometrium and maintaining early pregnancy. Decidualization is the transformation of quiescent endometrial stromal fibroblasts into specialized decidual stromal cells. Although the precise prevalence of decidualization defects in infertile women remains unclear, a deeper understanding of the molecular mechanisms governing decidualization is essential to improve implantation success and pregnancy outcomes in ART.

Decidualization is tightly regulated by ovarian steroid hormones, transcription factors, morphogens, cytokines, and signaling cascades that coordinate endometrial remodeling during early pregnancy [5, 6]. However, the knowledge on the epigenetic regulatory machinery governing uterine decidualization remains limited. In addition to these signaling molecules, epigenetic mechanisms have emerged as critical regulators of uterine receptivity and decidualization. Epigenetic regulation refers to heritable and reversible modifications that influence gene expression without altering DNA sequences, primarily through DNA methylation, histone modifications, and noncoding RNAs. Increasing evidence shows that steroid hormone‐dependent transformation of the endometrium relies on dynamic epigenetic modifications that control chromatin accessibility and transcriptional programs essential for endometrial function and decidualization [7]. Among these mechanisms, histone acetylation and deacetylation play pivotal roles in cyclical endometrial remodeling. Aberrant histone deacetylase (HDAC) activity has been linked to several uterine pathologies, including endometrial cancer, endometriosis, and infertility [8, 9, 10, 11].

Histone deacetylases (HDACs) are grouped into four classes based on structure, cofactor dependence, and evolutionary conservation [12, 13]. Class I HDACs (HDAC1, 2, 3, and 8) are primarily nuclear enzymes that regulate gene expression, cell proliferation, and differentiation. Among them, histone deacetylase 3 (HDAC3) is unique in that it can localize to both the nucleus and cytoplasm, functioning through enzymatic and non‐enzymatic mechanisms [14, 15]. HDAC3 enzymatic activity requires its interaction with the deacetylase‐activating domain (DAD) of nuclear receptor corepressors NCoR1 and NCoR2 (SMRT), forming complexes that regulate transcriptional repression [16, 17, 18]. Beyond its classical role as a transcriptional repressor, HDAC3 also acts as a coactivator in inflammatory and metabolic signaling pathways, including IL‐1 and Notch signaling [19, 20, 21].

Our previous study demonstrated that HDAC3 expression is markedly reduced in the eutopic endometrium of infertile women with endometriosis compared to fertile controls, and uterine‐specific ablation of *Hdac3* in mice results in implantation failure and decidualization defects [11]. Mechanistically, loss of HDAC3 leads to aberrant transcriptional activation of *Col1a1* and *Col1a2*, disrupting extracellular matrix remodeling essential for decidualization. However, the broader transcriptomic and molecular mechanisms through which HDAC3 regulates the decidualization process remain incompletely understood.

To address this gap, we utilized an artificial decidualization model in uterine‐specific *Hdac3* knockout (*Pgr* ^*cre/+*^ *Hdac3* ^*f/f*^; *Hdac3* ^*d/d*^) mice to investigate the molecular and transcriptomic alterations associated with defective decidualization. RNA sequencing and pathway analyses revealed that HDAC3 ablation profoundly alters the expression of genes and signaling networks involved in cytoskeletal remodeling, oxidative stress regulation, and cellular metabolism. In particular, the RHOA, AMPK‐NOTCH1‐HEY1, and oxidative stress‐induced senescence pathways were significantly dysregulated in *Hdac3* ^*d/d*^ mice. These findings provide new insights into the epigenetic regulation of decidualization and identify HDAC3 as a pivotal transcriptional regulator of uterine function and early pregnancy establishment.

## 2 Materials and Methods

### 2.1 Animals

All animal procedures were approved by the University of Missouri Animal Care and Use Committee. Mice were housed in the designated animal care facility at the University of Missouri under controlled temperature and humidity conditions with a 12‐h light/dark cycle. Mice were randomly assigned to experimental groups, as there were no noticeable differences in size or appearance at baseline. Following dissection, uterine tissues were immediately collected for RNA isolation and immunohistochemistry.

### 2.2 Artificial Decidual Induction and Tissue Collection

*Hdac3* ^*f/f*^ and *Hdac3* ^*d/d*^ mice [11] were generated by breeding *Hdac3* ^*d/d*^ males with *Hdac3* ^*f/f*^ females. Six‐week‐old female mice were used for inducing artificial decidualization as described in a previous study [22]. *Hdac3* ^*f/f*^ and *Hdac3* ^*d/d*^ mice were ovariectomized and allowed to recover for 2 weeks. Then mice were subjected to the following hormonal regimen: 100 ng of E2 per day for 3 days; 2 days rest; then three daily injections of 1 mg of P4 plus 6.7 ng of E2. After 6 h of the third P4 and E2 injection, a single uterine horn of each mouse was mechanically stimulated by scratching the full length of the anti‐mesometrial side with a burred needle. Another horn was left unstimulated as a control. Mice then were continuously injected P4 (1 mg/mouse) + E2 (6.7 ng/mouse) daily following mechanical stimulation, and uterine tissues were collected at 6 h after hormone administration on days 1, 3, and 5. At each time point, mice were euthanized, and the stimulated and control uterine horns were excised and weighed. The ratio of stimulated horns and control horns was calculated by dividing the weights of stimulated horns by those of control horns. Uterine tissues were immediately either fixed with 4% (w/v) paraformaldehyde for immunohistochemistry or frozen in dry ice and then stored at −80°C for RNA isolation.

### 2.3 RNA Isolation and RNA Sequencing Analysis

Total RNA was isolated from the frozen uterine tissues using QIAGEN RNeasy Mini kit (Qiagen, Valencia, CA). RNA purification and concentration were assessed by using UV spectroscopy with a NanoDrop instrument. All RNA samples were divided for RT‐qPCR and RNA‐seq analysis. RNA library formation and indexing was done by the Genomic Technology Core in University of Missouri. RNA fragments of 300–400 bp were obtained by preparing libraries from 500 ng of total RNA using the KAPA mRNA HyperPrep kit (Kapa Biosystems, Wilmington, MA). Prior to PCR amplification, cDNA fragments were ligated using Illumina TruSeq UD Indexed adapters (Illumina Inc., San Diego, CA) with IDT. The QuantiFluor dsDNA System (Promega Corp., Madison, WI) and Agilent DNA High Sensitivity chip (Agilent Technologies Inc.) were used to assess the concentration and quality of the library. Subsequently using the S2, a 100‐bp kit, the tagged libraries were combined and sequenced on an Illumina NovaSeq6000, yielding 50 bp paired end reads with an average of 50 million reads per sample. Base calling was carried out with Illumina RTA3, and Bcl2fastq v1.9.0 was used to demultiplex and convert the data to FastQ format. After filtering out low‐quality reads (average score < 20), the remaining reads were matched to the mm39 genome using STAR 2.7.9a [23]. Expression values were represented by counts and using EdgeR's exact negative binomial test [24], DEGs were found between *Hdac3* ^*f/f*^ and *Hdac3* ^*d/d*^ mice (*n* = 4). Criteria were set at fold change > ±2 and *p*‐value < 0.05. Tidyverse and ComplexHeatmap programs were used to create data visualizations, and Ingenuity Pathway Analysis (IPA; Qiagen Inc., CA, USA) was utilized for pathway and upstream regulator analysis [25].

### 2.4 RT‐qPCR

For RT‐qPCR, Complementary DNA (cDNA) was synthesized by using 1 μg of RNA with MMLV Reverse Transcriptase (Invitrogen, Carlsbad, CA) according to the manufacturer's instructions. Relative expressions of decidual markers, *Wnt4* and *Bmp2*, were measured by real‐time PCR with SYBR green analysis using an Applied Biosystems StepOnePlus system (Applied Biosystems, Foster City, CA, USA). The mRNA quantities were normalized against the housekeeping gene, 60S ribosomal protein L7. Analysis of mRNA expression was first undertaken by the standard curve method, and results were corroborated by cycle threshold values assessing gene expression. Primer sequences of *Wnt4*, *Bmp2*, and *Rpl7* are shown in Table 1.

**TABLE 1:** List of primers used in RT‐qPCR analysis.

Gene	Primer sequences
m*Bmp*2	
Forward	5′‐AGATCTGTACCGCAGGCACT‐3′
Reverse	5′‐CGTCACTGGGGACAGAACTT‐3′
m*Wnt4*	
Forward	5′‐ACAACACACACCAGTACGCCC‐3′
Reverse	5′‐GTTACACTTGACGTAGCAGCACCA‐3′
m*Rpl*7	
Forward	5′‐TCAATGGAGTAAGCCCAAAG‐3
Reverse	5′‐CAAGAGACCGAGCAATCAAG‐3′

### 2.5 Immunohistochemistry

Immunohistochemistry was performed as previously described [26]. Dewaxed, hydrated paraffin‐embedded tissue sections were blocked with 10% normal goat serum (NGS, v/v, Vector Laboratories) in phosphate‐buffered saline (PBS, pH 7.5) and then incubated overnight at 4°C with rabbit primary antibody anti‐LIMK1 (1:500 dilution; 3842S; Cell Signaling), anti‐PRKAG1 (1:500 dilution; A7300; Abclonal), or anti‐CBX2 (1:500 dilution; 18687S; Cell Signaling) diluted in 10% NGS. The following day, sections were incubated with biotinylated anti‐rabbit IgG secondary antibody (1:500 dilution; BA‐1000; Vector Laboratories) for 1 h at room temperature, followed by incubation with horseradish peroxidase (1:1000 dilution; 43–4323; Invitrogen) for 45 min. After PBS washes, immunoreactivity was detected using the Vectastain Elite DAB kit (Vector Laboratories). A semiquantitative grading system (H‐score) was used to compare the immunohistochemical staining intensities as previously described [27]. The overall score ranged from 0 to 300.

### 2.6 Statistical Analysis

Statistical significance of parametric data was obtained by performing one‐way analysis of variance (ANOVA) and Tukey's post hoc test for multiple comparisons. Students' *t*‐test were used to compare between two groups. Each experiment included at least four biological replicates. All statistical analyses were conducted using GraphPad Prism version 10 (GraphPad Software, San Diego, CA). A *p*‐value < 0.05 was considered statistically significant.

## 3 Results

### 3.1 Uterine HDAC3 Loss Causes an Early‐Stage Decidualization Defect

**FIGURE 1:**
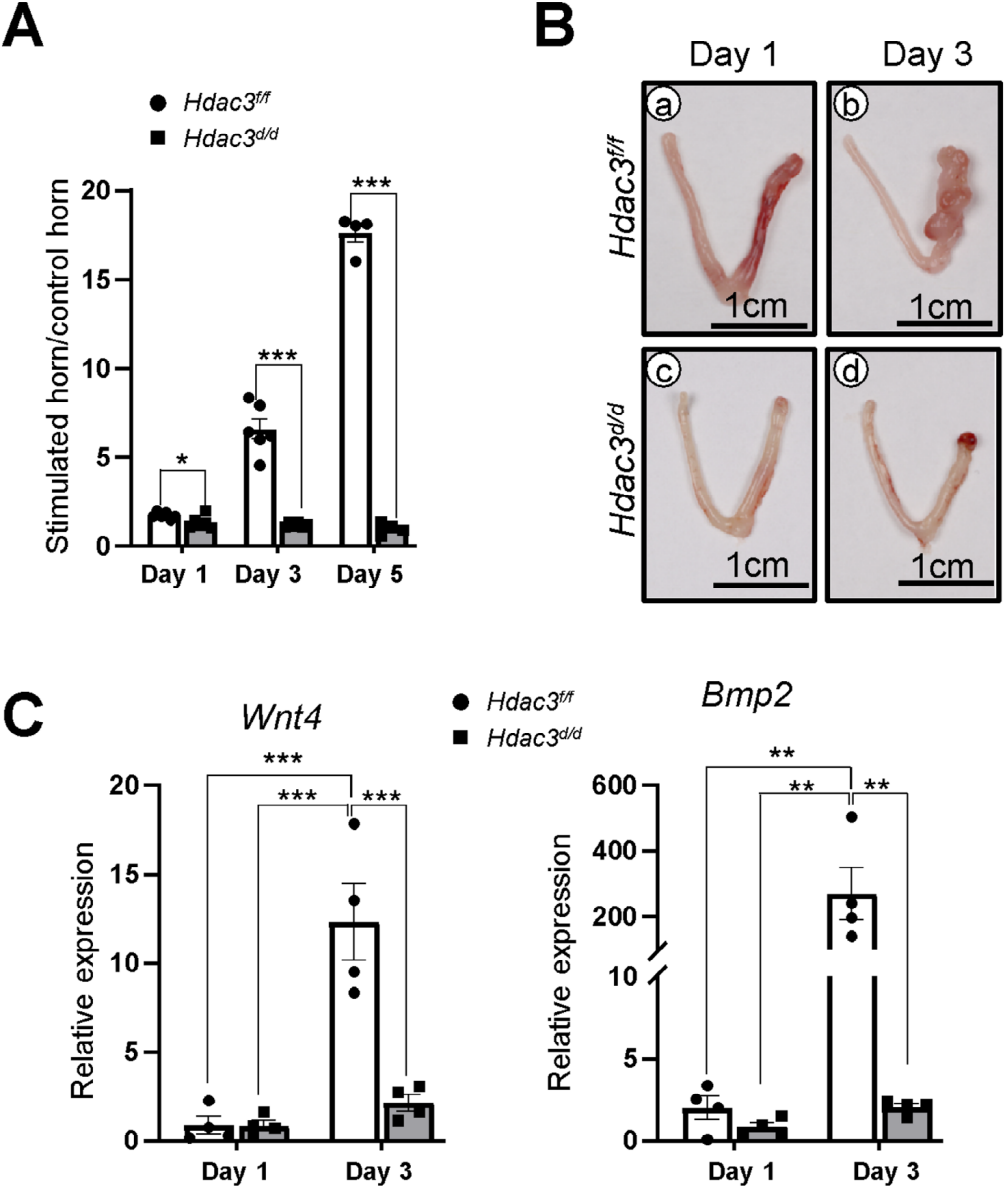
Decidualization defects detected in *Hdac3* ^*d/d*^ mice. (A) Decrease of the stimulated‐to‐control uterine weight ratio in *Hdac3* ^*f/f*^ and *Hdac3* ^*d/d*^ mice at decidualization day 1, 3, and 5. (B) Representative images of the uteri of *Hdac3* ^*f/f*^ and *Hdac3* ^*d/d*^ mice at decidualization day 1 and 3. (C) RT‐qPCR analysis for the expression of decidualization marker genes, *Wnt4* and *Bmp2*, in the uterus of *Hdac3* ^*f/f*^ and *Hdac3* ^*d/d*^ mice on day 1 and 3 of decidualization (*n* = 6 for each group at day 1 and 3). The results represent the mean ± SEM. *p < 0.05, **p < 0.01, ***p < 0.001 by ordinary one‐way ANOVA test.

To determine the effect of HDAC3 loss during decidualization, we applied artificial decidualization to control *Hdac3* ^*f/f*^ and *Hdac3* ^*d/d*^ mice. The stimulated and unstimulated uterine horns were collected on days 1, 3, and 5 to assess the decidual response (Figure 1A). In control mice, the stimulated uterine horn exhibited an initial decidual response after day 1 of mechanical stimulation, which markedly increased by day 3; representative morphological changes at day 5, consistent with our previous study [11], are not shown here to avoid redundancy. No response was observed in the unstimulated horns. In contrast, *Hdac3* ^*d/d*^ mice showed minimal thickening and enlargement from day 1 to day 3, and remained markedly impaired at day 5, indicating a severely impaired decidualization process during the early phase. The ratio of the stimulated to unstimulated uterine horn weight was significantly lower in *Hdac3* ^*d/d*^ mice compared to controls at all three time points, with the ratio in control mice being nearly fourfold higher by day 3. To further confirm these morphological findings at day 3, we examined the expression of *Wnt4* and *Bmp2*, two well‐established markers of decidualization [11]. Because expression of these markers at day 5 has been previously reported [11], we focused here on earlier time points to capture initial defects in decidualization rather than repeating established findings. Expression of *Wnt4* and *Bmp2* was robustly upregulated in the stimulated uterine horn of control mice after 3 days. However, their expression was significantly reduced in the stimulated uterine horns of *Hdac3* ^*d/d*^ mice compared to control mice (Figure 1C). These results indicate that uterine‐specific loss of *Hdac3* impairs early stages of decidualization.

### 3.2 Transcriptomic Profiling Identifies HDAC3‐Regulated Pathways During Decidualization

**FIGURE 2:**
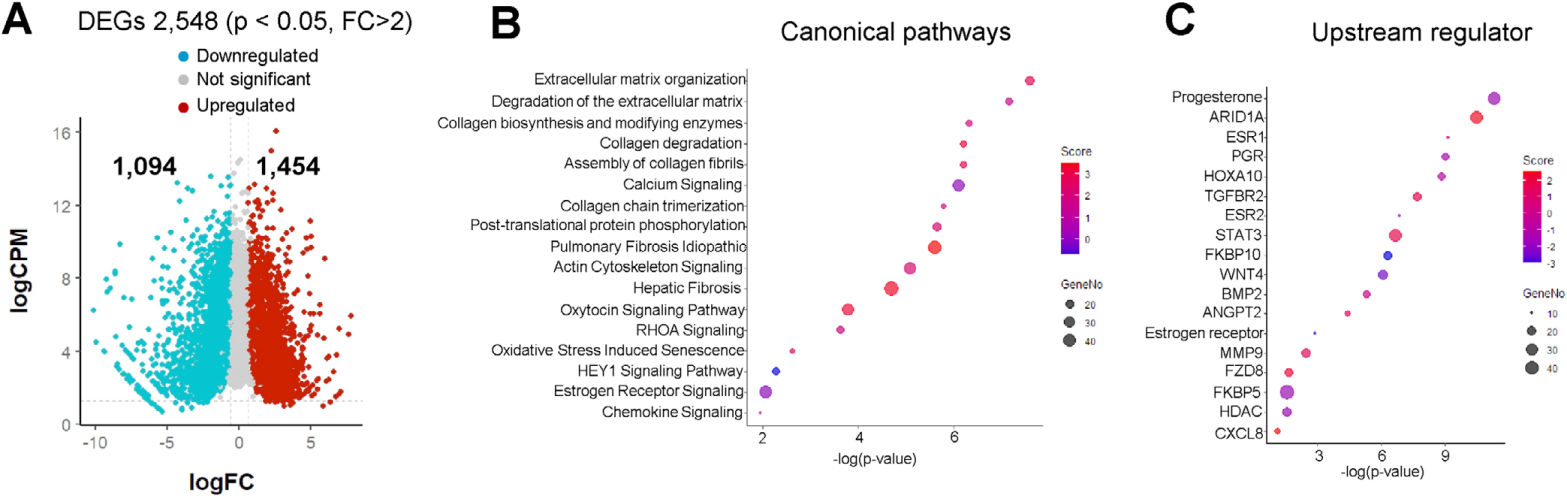
Transcriptome profile of stimulated horn in uterus of *Hdac3* ^*f/f*^ and *Hdac3* ^*d/d*^ mice at decidualization day 3. (A) Volcano plot showing the 2548 differentially expression genes (DEGs) with a fold change (FC) > ±2 and *p*‐value < 0.05 in *Hdac3* ^*d/d*^ mice uteri compared with the *Hdac3* ^*f/f*^ (control) mice (*n* = 4). Red dots represent the upregulated, teal dots represent downregulated and gray dots represent non‐significant DEGs in the data sets. (B) Dot plot illustrating the top 17 enriched canonical pathways in the stimulated horn of *Hdac3* ^*d/d*^ compared to the *Hdac3* ^*f/f*^ mouse uteri. (C) Dot plot showing the top 18 upstream regulators in the stimulated horn of *Hdac3* ^*d/d*^ compared to *Hdac3* ^*f/f*^ mouse uteri. Dot color represents the corresponding *z*‐score, and dot size reflects the number of DEGs involved in each pathway.

*Hdac3* ^*d/d*^ mice showed defective decidualization as early as day 3, establishing this as a key time point for molecular assessment. To identify the signaling pathways regulated by *Hdac3* during decidualization, RNA‐seq was performed on the stimulated uterine horns of *Hdac3* ^*f/f*^ and *Hdac3* ^*d/d*^ mice on day 3 (*n* = 4 for each group) (Figure 2). After alignment to the mouse reference genome and filtering out low‐expression transcripts, a total of 8004 genes were identified. Differential expression analysis revealed 2548 differentially expressed genes (DEGs) between *Hdac3* ^*f/f*^ and *Hdac3* ^*d/d*^ mice, including 1094 downregulated and 1454 upregulated genes (fold change ≥ 2 or ≤ −2, *p*‐value ≤ 0.05) (Figure 2A; Table S1). Pathway enrichment analysis using Ingenuity Pathway Analysis (IPA) revealed significant alterations in canonical pathways related to fibrosis, post‐translational modification, cytoskeletal remodeling, oxidative stress, extracellular matrix (ECM) organization, and chemokine signaling (Figure 2B). Consistent with previous findings, IPA upstream regulator analysis showed that several decidualization‐related genes, including *Pgr*, *Wnt4*, and *Bmp2*, were downregulated in *Hdac3* ^*d/d*^ uteri (Figure 2C). In addition, suppression of RHOA signaling, oxidative stress‐induced senescence, and AMPK‐NOTCH1‐HEY1 signaling pathways were identified. Given their potential involvement in uterine remodeling and cellular differentiation, we further investigated the contribution of these pathways to the decidualization process.

### 3.3 HDAC3 Loss Disrupts RHOA Signaling During Decidualization

**FIGURE 3:**
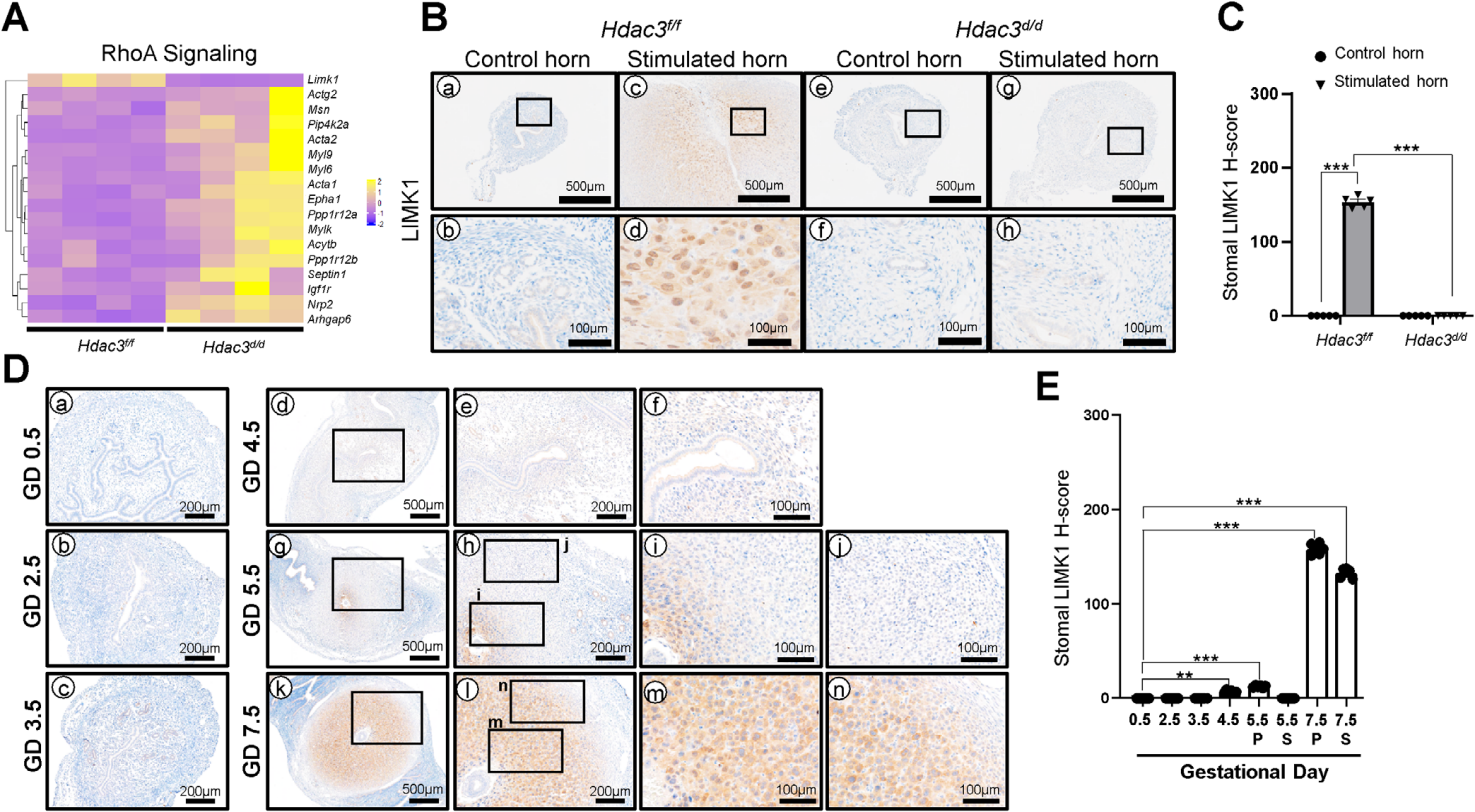
Loss of *Hdac3* in uterine results in decidualization defect due to dysregulation of RhoA Signaling. (A) Heatmap demonstrating the RhoA signaling‐regulatory genes in the stimulated horn in uterus of *Hdac3* ^*d/d*^ mice compared with that of *Hdac3* ^*f/f*^ mice. (B) Representative images of immunohistochemistry (IHC) of LIMK1 expression in the uteri of *Hdac3* ^*f/f*^ and *Hdac3* ^*d/d*^ mice on days 1 and 3 of decidualization (*n* = 6 for each group). (C) Semi‐quantitative analysis of LIMK1 levels in the uterus of *Hdac3* ^*f/f*^ and *Hdac3* ^*d/d*^ mice on days 1 and 3 of decidualization. (D) Representative IHC image of LIMK1 expression of the uterine tissues from gestation day (GD) 0.5 to 7.5 (*n* = 5 per time point). (E) Semi‐quantitative analysis of LIMK1 expression levels in the uterine tissue from GD 0.5 to GD 7.5. Quantification for stromal cells in GD 5.5 and GD 7.5 uterine sections was analyzed and plotted separately to distinguish primary (P) and secondary (S) decidualized cells. The results represent the mean ± SEM. **p < 0.01, ***p < 0.001 by ordinary one‐way ANOVA test.

RhoA activation plays a crucial role in the formation of stress fibers and organization of the actin cytoskeleton during decidualization in human endometrial stromal cells (hESCs) [28]. LIM kinase 1 (LIMK1), a downstream effector of RhoA signaling, phosphorylates cofilin, an actin‐depolymerizing factor, and regulates actin cytoskeletal reorganization through the RhoA‐ROCK‐LIMK1 pathway [29]. Pathway analysis using IPA identified 18 differentially expressed RhoA‐related genes in the stimulated uterine horns of *Hdac3* ^*d/d*^ mice compared with controls (Figure 3A). To further assess the involvement of RhoA signaling in HDAC3‐mediated decidualization, we examined LIMK1 expression in decidualized uteri of *Hdac3* ^*f/f*^ and *Hdac3* ^*d/d*^ mice using immunohistochemical analysis (Figure 3B). LIMK1 expression was undetectable in the unstimulated horns of both *Hdac3* ^*f/f*^ and *Hdac3* ^*d/d*^ mice. In contrast, strong LIMK1 staining was observed in decidual cells of the stimulated uterine horns from *Hdac3* ^*f/f*^ mice but was markedly reduced in *Hdac3* ^*d/d*^ mice. Quantitative H‐score analysis confirmed a significant reduction of LIMK1 expression in *Hdac3* ^*d/d*^ mice compared to controls (Figure 3C).

To determine the physiological expression pattern of LIMK1 during early pregnancy, we evaluated its localization in the mouse uteri from gestational day (GD) 0.5 to GD 7.5 (Figure 3D,E). LIMK1 was not detected in stromal, luminal, or glandular epithelial cells from GD 0.5 to GD 3.5. At GD 4.5, weak LIMK1 expression appeared in stromal cells but was absent in epithelial compartments. At GD5.5, LIMK expression became prominent in the primary decidual zone (PDZ) and weaker in the secondary decidual zone (SDZ). At GD 7.5, LIMK1 expression increased further in both PDZ and SDZ, with stronger staining in the PDZ. These results indicate that HDAC3 loss leads to dysregulation of RHOA signaling in the uterus during decidualization, likely through impaired LIMK1 expression and downstream cytoskeletal remodeling.

### 3.4 HDAC3 Loss Dysregulates AMPK‐NOTCH1‐HEY1 Signaling During Decidualization

**FIGURE 4:**
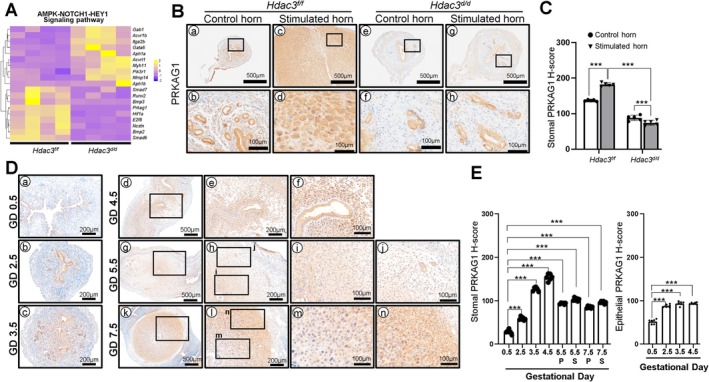
AMPK‐NOTCH1‐HEY1 signaling pathway involvement in the decidualization defect of *Hdac3*‐ablated mice uteri. (A) Heatmap showing the expression of AMPK‐NOTCH1‐HEY1 signaling pathway‐regulatory genes in the stimulated horn of *Hdac3* ^*d/d*^ mice compared to the *Hdac3* ^*f/f*^ mice uteri. (B) Representative IHC images of PRKAG1 in the *Hdac3* ^*f/f*^ and *Hdac3* ^*d/d*^ mice uteri on days 1 and 3 of decidualization (*n* = 6 for each group). (C) Semi‐quantitative analysis of PRKAG1 expression patterns in the *Hdac3* ^*f/f*^ and *Hdac3* ^*d/d*^ mice uteri on decidualization days of 1 and 3. (D) Representative IHC images of PRKAG1 of the uterine tissues from GD 0.5 to GD 7.5 (*n* = 5 per time point). (E) Semi‐quantitative analysis of PRKAG1 expression patterns in the stromal (left) and epithelial (right) cells from GD 0.5 to GD 7.5. Quantification for stromal cells in GD 5.5 and GD 7.5 uterine sections was analyzed and plotted separately to distinguish primary (P) and secondary (S) decidualized cells. The results represent the mean ± SEM. ***p < 0.001 by ordinary one‐way ANOVA test.

In addition to its role in RHOA signaling, transcriptomic analysis revealed dysregulation of the AMPK‐NOTCH1‐HEY1 signaling pathway in *Hdac3* ^*d/d*^ mice (Figure 4A). To further investigate this finding, we examined PRKAG1, a regulatory subunit of AMPK signaling that has been reported to modulate NOTCH1‐HEY1 signaling through metabolic regulation, in the uteri of *Hdac3* ^*f/f*^ and *Hdac3* ^*d/d*^ mice (Figure 4B). In control mice, PRKAG1 expression was weakly detected in the unstimulated horn but strongly induced in decidual cells of the stimulated horn following artificial decidualization, while expression in glandular epithelium remained largely unchanged. In contrast, PRKAG1 expression was significantly reduced in both the unstimulated and stimulated horns of *Hdac3* ^*d/d*^ mice (Figure 4C) with no notable changes observed in glandular compartments.

To determine the physiological expression pattern of PRKAG1 during early pregnancy, we evaluated its localization in mouse uteri from GD 0.5 to GD 7.5 (Figure 4D,E). PRKAG1 was weakly expressed in stromal and epithelial cells at GD0.5, gradually increased at GD2.5 and GD3.5, and reached peak expression in stromal cells at GD4.5. At GD5.5, PRKAG1 expression slightly decreased in both PDZ and SDZ, with stronger expression in the SDZ than in the PDZ. A similar expression pattern was maintained at GD7.5. These findings suggest that HDAC3 regulates decidualization in part through coordination of metabolic (AMPK) and transcriptional (NOTCH1‐HEY1) signaling pathways, which are functionally linked during stromal cell decidualization.

### 3.5 HDAC3 Loss Dysregulates the Oxidative Stress‐Induced Senescence Pathway

**FIGURE 5:**
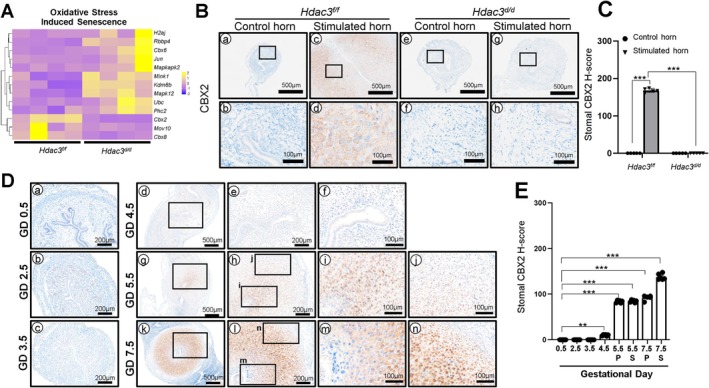
Loss of *Hdac3* in uterine results in decidualization defect due to dysregulation of Oxidative Stress‐Induced Senescence pathway. (A) Heatmap showing the gene expressions regulating the Oxidative Stress‐Induced Senescence pathway in the stimulated horn of *Hdac3* ^*d/d*^ mice compared to the *Hdac3* ^*f/f*^ mice uteri. (B) Representative IHC images of CBX2 in the uterus of *Hdac3* ^*f/f*^ and *Hdac3* ^*d/d*^ mice on days 1 and 3 of decidualization (*n* = 6 for each group). (C) Semi‐quantitative analysis of CBX2 expression levels in the uterus of *Hdac3* ^*f/f*^ and *Hdac3* ^*d/d*^ mice on days 1 and 3 of decidualization. (D) Representative IHC images of CBX2 in the uterine tissues from GD 0.5 to GD 7.5 (*n* = 5 per time point). (E) Semi‐quantitative analysis of CBX2 in the uterine tissues from GD 0.5 to GD 7.5. Quantification for stromal cells in GD 5.5 and GD 7.5 uterine sections was analyzed and plotted separately to distinguish primary (P) and secondary (S) decidualized cells. **p < 0.01, ***p < 0.001 by ordinary one‐way ANOVA test.

Pathway analysis revealed that the oxidative stress‐induced senescence signaling pathway was upregulated in the uteri of *Hdac3* ^*d/d*^ mice compared with controls (Figure 5A). Oxidative stress contributes to tissue damage and plays a key role in the pathogenesis of female infertility by reducing embryo implantation rates and impairing uterine decidualization. It is also implicated in various reproductive disorders, including endometriosis, recurrent pregnancy loss, and preeclampsia [30, 31, 32]. Elevated oxidative stress leads to DNA damage, often indicated by increased levels of 8‐hydroxy‐2′‐deoxyguanosine (8‐OHdG), which can induce cellular senescence [33, 34]. Premature decidual senescence has been shown to cause implantation failure, fetal loss, and preterm birth [5]. To further investigate this finding, we examined the expression of CBX2, a gene associated with oxidative stress and cellular senescence. Strong CBX2 expression was observed in the stimulated uterine horns of *Hdac3* ^*f/f*^ mice, whereas no CBX2 expression was detected in the control horns of *Hdac3* ^*f/f*^ mice or in either horn of *Hdac3* ^*d/d*^ mice (Figure 5B,C).

We next evaluated CBX2 expression during early pregnancy (Figure 5D,E). CBX2 was undetectable in stromal and epithelial cells from GD 0.5 to GD 3.5. From GD 4.5 onward, CBX2 expression gradually increased in stromal cells but remained absent in the epithelial compartments. At GD 5.5, CBX2 expression was comparable between PDZ and SDZ. By GD 7.5, CBX2 expression became markedly stronger in the SDZ compared with the PDZ. These findings suggest that HDAC3 loss leads to aberrant activation of oxidative stress‐induced senescence signaling, accompanied by reduced CBX2 expression, which may contribute to defective decidualization and impaired uterine function.

## 4 Discussion

This study provides strong evidence that HDAC3 plays an essential role in regulating endometrial decidualization. Our previous work demonstrated that HDAC3 is expressed in both human endometrial epithelial and stromal cells and is markedly reduced in the eutopic endometrium of infertile women with endometriosis [11], a condition associated with impaired decidualization and implantation failure. This clinical observation provided a rationale for using a uterine‐specific *Hdac3* knockout mouse model to investigate the mechanistic role of HDAC3 in decidualization. Accordingly, in the current study, we focused on stromal cells and revealed that uterine‐specific loss of *Hdac3* disrupts decidualization. Using transcriptomic analysis and functional validation, we identified three key downstream effectors, PRKAG1, LIMK1, and CBX2, whose expression was significantly reduced in *Hdac3* ^*d/d*^ uteri during decidualization. These findings highlight the broad transcriptional and signaling dysregulation caused by HDAC3 deficiency in the endometrium.

Decidualization is a hormone‐dependent differentiation process essential for embryo implantation and pregnancy maintenance. It involves extensive cellular remodeling, metabolic adaptation, and immune modulation within the uterine stroma. Impaired decidualization contributes to infertility, recurrent miscarriage, and uteroplacental disorders [35]. In agreement with our previous findings in human endometrium, *Hdac3* ^*d/d*^ mice exhibited a marked decidualization defect, suggesting that HDAC3 is indispensable for the transcriptional reprogramming required for uterine receptivity. RNA‐seq analysis revealed that multiple signaling pathways, including AMPK‐NOTCH1‐HEY1, RHOA, and oxidative stress‐induced senescence, were altered in the absence of HDAC3, indicating its broad regulatory role in cytoskeletal organization, energy metabolism, and stress responses during early pregnancy. Among these, the AMPK‐NOTCH1‐HEY1 axis represents a key integrative pathway linking cellular metabolism to transcriptional regulation during decidualization.

PRKAG1 encodes the γ1 regulatory subunit of AMP‐activated protein kinase (AMPK), a central sensor of cellular energy status that coordinates metabolism, growth, and stress responses [36, 37, 38, 39]. AMPK signaling has been shown to regulate NOTCH1‐HEY1 activity, linking cellular energy status to transcriptional control of cell differentiation [40]. It can also directly regulate HDAC complexes to alter gene transcription [41]. PRKAG1 overexpression has been reported in ovarian carcinomas [42], whereas its decline with aging correlates with increased frailty and metabolic dysfunction [43]. Although PRKAG1 has not been studied in the context of embryo implantation or decidualization, our results demonstrate that its expression is dynamically regulated in the uterus during early pregnancy, where it increases from GD 0.5 to GD 4.5 and is maintained in decidual cells at GD 5.5–7.5. The loss of HDAC3 led to downregulation of PRKAG1 and suppression of the AMPK‐NOTCH1‐HEY1 axis, suggesting that HDAC3 may facilitate decidualization through metabolic and transcriptional control of this pathway.

LIMK1, a downstream effector of the RHOA‐ROCK pathway, phosphorylates and inactivates cofilin to promote actin cytoskeletal reorganization [44]. Through its control of cytoskeletal dynamics, LIMK1 regulates critical cellular functions such as proliferation, migration, and differentiation [45, 46]. Aberrant LIMK1 expression has been implicated in multiple cancers, where it enhances invasion and metastasis [47, 48, 49, 50, 51]. In our study, LIMK1 was highly expressed in the decidual cells of control mice but nearly absent in *Hdac3* ^*d/d*^ uteri. During normal pregnancy, LIMK1 expression emerged in stromal cells by GD4.5 and intensified in PDZ and SDZ by GD 7.5. These observations suggest that HDAC3 may regulate RHOA‐LIMK1‐cofilin signaling to coordinate cytoskeletal remodeling necessary for stromal cell transformation during decidualization.

CBX2 is a chromobox family protein and a component of the Polycomb Repressive Complex 1 (PRC1), acting as an epigenetic reader that binds H3K9me3 and H3K27me3 to maintain transcriptional repression [52, 53, 54, 55, 56]. CBX2 overexpression has been associated with tumorigenesis, while HDAC inhibition downregulates CBX2, leading to growth arrest and apoptosis [57]. CBX2 also promotes proliferation and invasion through PI3K/AKT signaling [58], a pathway critical for decidualization [59, 60]. In our study, CBX2 expression was markedly reduced in *Hdac3* ^*d/d*^ uteri but strongly induced in decidual cells of control mice. During early pregnancy, CBX2 appeared in stromal cells by GD4.5 and progressively increased through GD7.5, suggesting a temporal role in stromal cell differentiation. These results imply that HDAC3 may influence chromatin organization and epigenetic stability during decidualization via regulation of CBX2 expression and activity.

Although our findings establish a critical role for HDAC3 in regulating decidualization and identify PRKAG1, LIMK1, and CBX2 as potential downstream effectors, several limitations should be acknowledged. First, our analyses were primarily based on whole uterine tissues, which may mask cell type‐specific transcriptional and signaling changes within the complex uterine microenvironment. Future studies employing single‐cell or spatial transcriptomic approaches would help define the specific cellular populations and regulatory networks directly governed by HDAC3. Second, while our data demonstrate strong correlations between HDAC3 loss and altered signaling pathways, the causal molecular interactions between HDAC3 and these downstream targets remain to be elucidated. Functional rescue or chromatin immunoprecipitation (ChIP) studies are needed to confirm direct transcriptional regulation. Third, this study relied on mouse models, and additional validation using human endometrial tissues or organoid systems will be essential to confirm translational relevance. Finally, although our model captures early stages of decidualization, it does not fully address how HDAC3 loss affects later implantation or placental development, which warrants further investigation.

Collectively, our findings demonstrate that HDAC3 orchestrates multiple transcriptional and signaling networks—metabolic (PRKAG1), cytoskeletal (LIMK1), and epigenetic (CBX2)—that are essential for uterine stromal differentiation and successful decidualization. Loss of HDAC3 disrupts these pathways, leading to defective decidual responses and potential implantation failure. These results expand our understanding of the epigenetic control of endometrial receptivity and identify HDAC3 as a central regulator linking metabolism, cytoskeletal remodeling, and chromatin dynamics during early pregnancy. Further studies are needed to define the precise molecular interactions between HDAC3 and its downstream effectors PRKAG1, LIMK1, and CBX2, including their post‐translational regulation and recruitment to chromatin during decidualization. Understanding these mechanisms may provide a foundation for developing targeted epigenetic or metabolic therapies to restore uterine function in infertility and pregnancy disorders associated with defective decidualization.

## Author Contributions

J‐.W.J. and Y‐.J.Y. were responsible for the concept of the study. L.T.K.N., D.N.T., and T.H.K. carried out experiments. L.T.K.N., D.N.T., S.N., T.H.K, and J‐.W.J. analyzed data. L.T.K.N., D.N.T., S.N., T.H.K, J‐.W.J., and Y‐.J.Y. contributed to writing the manuscript. All authors contributed to editing the manuscript.

## Funding

This work was supported by HHS | NIH | Eunice Kennedy Shriver National Institute of Child Health and Human Development (NICHD), R01HD101243 and R01HD102170 to J.‐W.J., and by the National Research Foundation of Korea (NRF) funded by the Korean government (MSIT), 2022R1A2C1091055, to J.‐Y.Y..

## Conflicts of Interest

Jae‐Wook Jeong serves as an editorial board member for the FASEB Journal and was recused from any editorial processes during the peer review of this manuscript, including the final decision on potential publication.

## Supporting information


**Table S1:** Differentially expressed genes between Hdac3 f/f and Hdac3 d/d mice uteri at decidualization day 3.

## Data Availability

The authors declare that all the relevant data, supplemental data, associated protocols, and materials supporting the findings of this study are present in the paper. The raw and processed RNA‐sequencing data generated in this study have been deposited in the NCBI Gene Expression Omnibus (GEO) under accession number GSE318623.
