# Supplementary material for: HDAC3 Regulates Transcriptional Networks Governing Decidualization
Source: FASEB J. 2026 Apr 15;40(8):e71815. doi: 10.1096/fj.202600629R (PMC13082311; doi:10.1096/fj.202600629R)
Supplement: Supplementary file 1 — Table S1: Differentially expressed genes between Hdac3 f/f and Hdac3 d/d mice uteri at decidualization day 3. [file FSB2-40-e71815-s001.pdf]

Supplementary Table 1. Differentially expressed genes between *Hdac3*$^{f/f}$ and *Hdac3*$^{d/d}$ mice uteri at decidualization day 3.

Essemble ID	Gene Symbol	Gene Description	Fold Change	P Value
ENSMUSG00000059213	Ddn	dendrin	-1130.05	0.00
ENSMUSG00000042401	Crtac1	cartilage acidic protein 1	-1008.93	0.00
ENSMUSG00000100430	1700095A13Rik	RIKEN cDNA 1700095A13 gene	-691.94	0.00
ENSMUSG00000091897	Gm17019	predicted gene 10471	-678.61	0.00
ENSMUSG00000044447	Dock5	dedicator of cytokinesis 5	-609.38	0.00
ENSMUSG00000114303	Gm4022	predicted gene 4022	-594.27	0.00
ENSMUSG00000028838	Extl1	exostosin like glycosyltransferase 1	-520.01	0.00
ENSMUSG00000053453	Thoc7	THO complex subunit 7	-412.01	0.00
ENSMUSG00000028601	Echdc2	enoyl-CoA hydratase domain containing 2	-401.00	0.00
ENSMUSG00000111417	Gm47430	predicted gene, 47430	-384.47	0.02
ENSMUSG00000018259	Prl8a2	prolactin family 8, subfamily a, member 2	-378.68	0.00
ENSMUSG00000111606	Gm7213	enolase 2, gamma neuronal pseudogene	-334.50	0.00
ENSMUSG00000113726	Gm36099	predicted gene, 36099	-317.00	0.00
ENSMUSG00000113303	4930423D22Rik	RIKEN cDNA 4930423D22 gene	-285.33	0.00
ENSMUSG00000072188	Gm10354	predicted gene 10471	-273.50	0.00
ENSMUSG00000060962	Dmkn	dermokine	-270.86	0.00
ENSMUSG00000067713	Prkag1	protein kinase AMP-activated non-catalytic subunit gamma 1	-263.31	0.00
ENSMUSG00000087339	Gm11586	predicted gene 11586	-242.18	0.00
ENSMUSG00000038300	Pth2	parathyroid hormone 2	-225.34	0.00
ENSMUSG00000110093	Gm45282	predicted gene 45282	-216.44	0.00
ENSMUSG00000094018	S100a2	S100 calcium binding protein A2	-199.63	0.00
ENSMUSG00000116989	Gm49709	predicted gene, 49709	-195.23	0.00
ENSMUSG00000105699	Gm43703	predicted gene 43703	-192.38	0.00
ENSMUSG00000090237	Gm16308	predicted gene 16308	-179.24	0.00
ENSMUSG00000028015	Ctso	cathepsin O	-174.51	0.00
ENSMUSG00000048217	Nags	N-acetylglutamate synthase	-165.69	0.00
ENSMUSG00000106185	Gm7631	UDP glucuronosyltransferase 2 family, polypeptide B1 pseudogene	-144.37	0.00
ENSMUSG00000113733	Gm34785	predicted gene, 34785	-142.59	0.00
ENSMUSG00000019214	Chtf18	chromosome transmission fidelity factor 18	-133.61	0.00
ENSMUSG00000072476	Rnf26rt	ring finger protein 26, retrotransposed	-133.39	0.00
ENSMUSG00000073208	Speer4c	predicted gene 10471	-133.37	0.00
ENSMUSG00000034533	Scn10a	sodium voltage-gated channel alpha subunit 10	-123.88	0.00
ENSMUSG00000034762	Glis1	GLIS family zinc finger 1	-123.51	0.00
ENSMUSG00000091255	Speer4e	predicted gene 10471	-121.22	0.00
ENSMUSG00000118333	4921531P14Rik	RIKEN cDNA 4921531P14 gene	-116.91	0.00
ENSMUSG00000099472	Gm29539	predicted gene 29539	-114.73	0.00
ENSMUSG00000034818	Celf5	CUGBP Elav-like family member 5	-106.34	0.02
ENSMUSG00000109116	Sycp1-ps1	synaptonemal complex protein 1, pseudogene 1	-104.80	0.00
ENSMUSG00000109016	Gm44647	predicted gene 44647	-103.66	0.00
ENSMUSG00000033219	Gm9758	predicted gene 10471	-103.00	0.00
ENSMUSG00000037451	Slc22a20	solute carrier family 22 (organic anion transporter), member 20	-102.51	0.00
ENSMUSG00000021913	Ogdhl	oxoglutarate dehydrogenase L	-100.46	0.00
ENSMUSG00000097727	F630040K05Rik	RIKEN cDNA F630040K05 gene	-98.06	0.00
ENSMUSG00000104568	Gm43255	predicted gene 43255	-94.72	0.00

Essemble ID	Gene Symbol	Gene Description	Fold Change	P Value
ENSMUSG00000022598	Psca	prostate stem cell antigen	-92.81	0.00
ENSMUSG00000114024	Gm46336	predicted gene, 46336	-91.09	0.05
ENSMUSG00000017922	Prl3c1	prolactin family 3, subfamily c, member 1	-88.50	0.00
ENSMUSG00000032323	Cyp11a1	cytochrome P450 family 11 subfamily A member 1	-85.03	0.00
ENSMUSG00000117987	Gm50222	predicted gene, 50222	-81.79	0.00
ENSMUSG00000034913	Cby2	chibby family member 2	-79.61	0.04
ENSMUSG00000018570	2810408A11Rik	RIKEN cDNA 2810408A11 gene	-70.29	0.00
ENSMUSG00000050463	Krt78	keratin 78	-68.13	0.04
ENSMUSG00000100361	Gm7063	apurinic/apyrimidinic endonuclease 1 pseudogene	-68.13	0.04
ENSMUSG00000086258	Gm15239	predicted gene 15239	-66.44	0.00
ENSMUSG00000121071	Gm57170	predicted gene, 57170	-66.21	0.03
ENSMUSG00000057899	Adgrf2	adhesion G protein-coupled receptor F2	-65.60	0.00
ENSMUSG00000046008	Pnlip	pancreatic lipase	-65.44	0.00
ENSMUSG00000079022	Col22a1	collagen type XXII alpha 1 chain	-64.87	0.00
ENSMUSG00000106114	Gm43286	predicted gene 43286	-61.62	0.01
ENSMUSG00000090589	Gm17180	predicted gene 17180	-60.50	0.00
ENSMUSG00000051648	Kctd19	potassium channel tetramerization domain containing 19	-58.02	0.00
ENSMUSG00000079602	Gata3un	Gata3 upstream neighbor	-56.55	0.00
ENSMUSG00000025400	Tac2	tachykinin precursor 3	-56.13	0.00
ENSMUSG00000113251	Gm18363	calcyclin binding protein pseudogene	-54.25	0.00
ENSMUSG00000107651	Gm44166	predicted gene, 44166	-54.21	0.00
ENSMUSG00000020921	Tmem101	transmembrane protein 101	-53.87	0.00
ENSMUSG00000096878	Gm21083	predicted gene, 21083	-53.65	0.00
ENSMUSG00000020258	Glyctk	glycerate kinase	-53.43	0.00
ENSMUSG00000089197	Gm25767	predicted gene, 25767	-53.03	0.04
ENSMUSG00000032611	1700102P08Rik	chromosome 3 open reading frame 62	-52.88	0.01
ENSMUSG00000029306	Ibsp	integrin binding sialoprotein	-50.90	0.03
ENSMUSG00000063821	Dusp29	dual specificity phosphatase 29	-50.90	0.00
ENSMUSG00000072573	Gm10369	predicted gene 10369	-49.86	0.00
ENSMUSG00000040364	Sec1	secretory blood group 1	-48.96	0.00
ENSMUSG00000082271	Gm12217	predicted gene 12217	-48.53	0.00
ENSMUSG00000107896	Gm8719	peptidyl prolyl isomerase H pseudogene	-47.99	0.00
ENSMUSG00000054003	Tdrd9	tudor domain containing 9	-47.33	0.00
ENSMUSG00000003410	Elavl3	ELAV like RNA binding protein 3	-45.50	0.02
ENSMUSG00000032105	Nherf4	NHERF family PDZ scaffold protein 4	-45.16	0.03
ENSMUSG00000011350	Gm5893	predicted gene 5893	-44.73	0.00
ENSMUSG00000080944	Gm12967	predicted gene 12967	-43.25	0.04
ENSMUSG00000056498	Tmem154	transmembrane protein 154	-43.12	0.00
ENSMUSG00000056162	Cndp1	carnosine dipeptidase 1	-42.90	0.00
ENSMUSG00000113996	Gm48455	predicted gene, 48455	-42.84	0.00
ENSMUSG00000086618	Gm13256	predicted gene 13256	-42.74	0.00
ENSMUSG00000113492	Gm47792	predicted gene, 47792	-42.72	0.00
ENSMUSG00000023918	Adgrf4	adhesion G protein-coupled receptor F4	-42.57	0.00
ENSMUSG00000026413	Pkp1	plakophilin 1	-41.34	0.04
ENSMUSG00000033294	Noc4l	nucleolar complex associated 4 homolog	-41.18	0.00
ENSMUSG00000120709	Gm56535	predicted gene, 56535	-40.89	0.00

Essemble ID	Gene Symbol	Gene Description	Fold Change	P Value
ENSMUSG00000024191	Bnip1	BCL2 interacting protein 1	-40.84	0.00
ENSMUSG00000047586	Nccrp1	NCCRP1, F-box associated domain containing	-40.61	0.00
ENSMUSG00000070564	Ntn5	netrin 5	-40.58	0.00
ENSMUSG00000021339	Mrs2	magnesium transporter MRS2	-40.21	0.00
ENSMUSG00000026167	Wnt10a	Wnt family member 10A	-40.06	0.00
ENSMUSG00000006567	Atp7b	ATPase copper transporting beta	-38.37	0.00
ENSMUSG00000085995	Gm2788	predicted gene 2788	-37.77	0.00
ENSMUSG00000097178	2310002F09Rik	RIKEN cDNA 2310002F09 gene	-36.88	0.00
ENSMUSG00000021768	Dusp13b	dual specificity phosphatase 13B	-35.52	0.00
ENSMUSG00000028836	Slc30a2	solute carrier family 30 member 2	-35.15	0.00
ENSMUSG00000034645	Zyg11a	zyg-11 family member A, cell cycle regulator	-34.05	0.00
ENSMUSG00000018822	Sfrp5	secreted frizzled related protein 5	-33.67	0.00
ENSMUSG00000092738	Mir5135	microRNA 5135	-33.52	0.00
ENSMUSG00000106445	Gm21190	predicted gene 10471	-30.92	0.00
ENSMUSG00000032842	Abcc10	ATP binding cassette subfamily C member 10	-30.80	0.00
ENSMUSG00000086108	Gm5602	predicted gene 5602	-29.85	0.00
ENSMUSG00000030111	A2m	alpha-2-macroglobulin	-29.33	0.00
ENSMUSG00000076441	Ass1	argininosuccinate synthase 1	-28.87	0.00
ENSMUSG00000005043	Sgsh	N-sulfoglucosamine sulfohydrolase	-28.33	0.00
ENSMUSG00000085843	Kank4os	KN motif and ankyrin repeat domains 4, opposite strand	-27.51	0.00
ENSMUSG00000115207	Gm49132	predicted gene, 49132	-27.48	0.00
ENSMUSG00000087541	Hopxos	HOP homeobox, opposite strand	-27.13	0.00
ENSMUSG00000099969	Gm28960	predicted gene 28960	-26.02	0.00
ENSMUSG00000097169	Gm26661	predicted gene, 26661	-25.34	0.00
ENSMUSG00000056600	Or2h2	olfactory receptor family 2 subfamily H member 2	-25.30	0.00
ENSMUSG00000020609	Apob	apolipoprotein B	-24.54	0.00
ENSMUSG00000042532	Golga7b	golgin A7 family member B	-24.35	0.00
ENSMUSG00000084775	Gm16741	predicted gene, 16741	-24.31	0.00
ENSMUSG00000032336	Nptn	neuroplastin	-24.08	0.00
ENSMUSG00000113338	A530046M15Rik	RIKEN cDNA A530046M15 gene	-23.74	0.00
ENSMUSG00000038201	Kcna7	potassium voltage-gated channel subfamily A member 7	-23.70	0.00
ENSMUSG00000086838	Gm16212	predicted gene 16212	-23.61	0.00
ENSMUSG00000107350	Gm19610	predicted gene, 19610	-22.76	0.00
ENSMUSG00000108432	Gm44756	predicted gene 44756	-22.25	0.00
ENSMUSG00000029335	Bmp3	bone morphogenetic protein 3	-22.13	0.00
ENSMUSG00000119981	Gm57382	predicted gene, 57382	-22.03	0.00
ENSMUSG00000113605	4930425K24Rik	RIKEN cDNA 4930425K24 gene	-21.79	0.00
ENSMUSG00000030376	Slc8a2	solute carrier family 8 member A2	-21.57	0.02
ENSMUSG00000028116	Myoz2	myozenin 2	-21.19	0.00
ENSMUSG00000113121	Gm35161	predicted gene, 35161	-20.50	0.00
ENSMUSG00000020649	Rrm2	ribonucleotide reductase regulatory subunit M2	-20.33	0.00
ENSMUSG00000060181	Slc35e3	solute carrier family 35 member E3	-19.87	0.00
ENSMUSG00000044824	Or55b10	olfactory receptor family 55 subfamily B member 10	-19.06	0.00
ENSMUSG00000059325	Hopx	HOP homeobox	-18.96	0.00
ENSMUSG00000086881	Gm13594	predicted gene 13594	-18.95	0.00
ENSMUSG00000027833	Shox2	SHOX homeobox 2	-18.84	0.00

Essemble ID	Gene Symbol	Gene Description	Fold Change	P Value
ENSMUSG00000112932	Gm48308	predicted gene, 48308	-18.61	0.00
ENSMUSG00000101801	1700020G17Rik	RIKEN cDNA 1700020G17 gene	-18.59	0.00
ENSMUSG00000085314	Gm14866	predicted gene 14866	-18.53	0.00
ENSMUSG00000039842	Mcph1	microcephalin 1	-18.48	0.00
ENSMUSG00000100510	Hand2os1	Hand2, opposite strand 1	-18.29	0.00
ENSMUSG00000039128	Cdc123	cell division cycle 123	-18.18	0.00
ENSMUSG00000112406	4921515L22Rik	RIKEN cDNA 4921515L22 gene	-18.14	0.00
ENSMUSG00000022952	Runx1	RUNX family transcription factor 1	-18.08	0.00
ENSMUSG00002076006	Gm56225	predicted gene, 56225	-17.98	0.00
ENSMUSG00000020099	Unc5b	unc-5 netrin receptor B	-17.67	0.00
ENSMUSG00000097891	Gm3650	predicted gene 3650	-17.66	0.01
ENSMUSG00000114935	Gm47040	predicted gene, 47040	-17.59	0.00
ENSMUSG00000096934	Gm26658	predicted gene, 26658	-17.42	0.00
ENSMUSG00000079434	Neu2	neuraminidase 2	-17.38	0.00
ENSMUSG00000037580	Gch1	GTP cyclohydrolase 1	-17.29	0.00
ENSMUSG00000021067	Sav1	salvador family WW domain containing protein 1	-17.26	0.00
ENSMUSG00000051397	Tacstd2	tumor associated calcium signal transducer 2	-17.05	0.00
ENSMUSG00000075249	Fsip2	fibrous sheath interacting protein 2	-17.04	0.00
ENSMUSG00000087067	Gm11532	predicted gene 11532	-17.01	0.00
ENSMUSG00000035365	Parpbp	PARP1 binding protein	-16.98	0.00
ENSMUSG00000102474	2610012C04Rik	RIKEN cDNA 2610012C04 gene	-16.90	0.00
ENSMUSG00000031245	Hmgn5	high-mobility group nucleosome binding domain 5	-16.83	0.00
ENSMUSG00000102545	6430573P05Rik	RIKEN cDNA 6430573P05 gene	-16.78	0.01
ENSMUSG00000034663	Bmp2k	BMP2 inducible kinase	-16.77	0.00
ENSMUSG00000082199	Gm15763	predicted gene 15763	-16.59	0.00
ENSMUSG00000025577	Cbx2	chromobox 2	-16.18	0.00
ENSMUSG00000030669	Calca	calcitonin related polypeptide beta	-16.10	0.00
ENSMUSG00000040446	Rprd1a	regulation of nuclear pre-mRNA domain containing 1A	-16.08	0.02
ENSMUSG00000120150	Gm56816	predicted gene, 56816	-16.04	0.04
ENSMUSG00000090293	Gm17034	predicted gene 17034	-16.03	0.00
ENSMUSG00000021221	Dpf3	double PHD fingers 3	-16.01	0.00
ENSMUSG00000024713	Pcsk5	proprotein convertase subtilisin/kexin type 5	-15.99	0.00
ENSMUSG00000037621	Atoh8	atonal bHLH transcription factor 8	-15.71	0.00
ENSMUSG00000053613	Notumos	notum palmitoleoyl-protein carboxylesterase, opposite strand	-15.69	0.00
ENSMUSG00000109697	Gm34030	predicted gene, 34030	-15.61	0.00
ENSMUSG00000035456	Prdm8	PR/SET domain 8	-15.40	0.00
ENSMUSG00000108362	Gm18561	TAR (HIV) RNA binding protein 2 pseudogene	-14.96	0.00
ENSMUSG00000095098	Ccdc85b	coiled-coil domain containing 85B	-14.63	0.00
ENSMUSG00000036553	Sh3tc1	SH3 domain and tetratricopeptide repeats 1	-14.59	0.00
ENSMUSG00000029334	Prkg2	protein kinase cGMP-dependent 2	-14.56	0.00
ENSMUSG00000045659	Plekha7	pleckstrin homology domain containing A7	-14.18	0.00
ENSMUSG00000027199	Gatm	glycine amidinotransferase	-14.03	0.00
ENSMUSG00000042842	Serpinb6b	serine (or cysteine) peptidase inhibitor, clade B, member 6b	-14.02	0.00
ENSMUSG00000033377	Palmd	palmdelphin	-13.99	0.00
ENSMUSG00000105506	Gm43743	predicted gene 43743	-13.80	0.00
ENSMUSG00000021765	Fst	follistatin	-13.74	0.00

Essemble ID	Gene Symbol	Gene Description	Fold Change	P Value
ENSMUSG00000044814	Or55b3	olfactory receptor family 55 subfamily B member 3	-13.36	0.00
ENSMUSG00000034981	Parm1	prostate androgen-regulated mucin-like protein 1	-13.29	0.00
ENSMUSG00000086448	9330162012Rik	cDNA RIKEN 9330162012 gene	-13.21	0.00
ENSMUSG00000043925	Or55b4	olfactory receptor family 55 subfamily B member 4	-12.93	0.00
ENSMUSG00000104784	Gm42984	predicted gene 42984	-12.83	0.01
ENSMUSG00000020572	Nampt	nicotinamide phosphoribosyltransferase	-12.81	0.00
ENSMUSG00000033857	Engase	endo-beta-N-acetylglucosaminidase	-12.60	0.00
ENSMUSG00000027358	Bmp2	bone morphogenetic protein 2	-12.52	0.00
ENSMUSG00000084873	C030047K22Rik	RIKEN cDNA C030047K22 gene	-12.46	0.01
ENSMUSG00000005148	Klf5	KLF transcription factor 5	-12.38	0.00
ENSMUSG00000001588	Acap1	ArfGAP with coiled-coil, ankyrin repeat and PH domains 1	-12.38	0.00
ENSMUSG00000110588	Gm45774	predicted gene 45774	-12.36	0.00
ENSMUSG00000018900	Slc22a5	solute carrier family 22 member 5	-12.35	0.00
ENSMUSG00000034039	Prss29	protease, serine 29	-12.29	0.01
ENSMUSG00000037624	Kcnk2	potassium two pore domain channel subfamily K member 2	-12.11	0.00
ENSMUSG00000034173	Zbed5	zinc finger BED-type containing 5	-12.01	0.01
ENSMUSG00000115098	Gm49134	predicted gene, 49134	-11.82	0.00
ENSMUSG00000030978	Rrm1	ribonucleotide reductase catalytic subunit M1	-11.77	0.00
ENSMUSG00000086683	Gm12867	predicted gene 12867	-11.61	0.00
ENSMUSG00000085838	Chn1os1	chimerin 1, opposite strand 1	-11.55	0.00
ENSMUSG00000085505	Gm12868	predicted gene 12868	-11.52	0.00
ENSMUSG00000106526	Gm42604	predicted gene 42604	-11.45	0.00
ENSMUSG00000118163	Gm41757	predicted gene, 41757	-11.45	0.00
ENSMUSG00000027496	Aurka	aurora kinase A	-11.41	0.00
ENSMUSG00000025551	Fgf14	fibroblast growth factor 14	-11.26	0.00
ENSMUSG00000097639	Platr4	pluripotency associated transcript 4	-11.24	0.00
ENSMUSG00000115959	Gm18494	fucosidase, alpha-L- 1, tissue pseudogene	-11.23	0.00
ENSMUSG00000084822	Myadml2os	myeloid-associated differentiation marker-like 2, opposite strand	-11.23	0.00
ENSMUSG00000058886	Deaf1	DEAF1 transcription factor	-11.17	0.00
ENSMUSG00000100303	2600014E21Rik	RIKEN cDNA 2600014E21 gene	-11.17	0.00
ENSMUSG00000101640	Gm28376	predicted gene 28376	-11.05	0.00
ENSMUSG00000050627	Gpd1l	glycerol-3-phosphate dehydrogenase 1 like	-11.01	0.00
ENSMUSG00000024302	Dtna	dystrobrevin alpha	-10.99	0.00
ENSMUSG00000085581	Gm12786	predicted gene 12786	-10.78	0.00
ENSMUSG00000033032	Afap1l1	actin filament associated protein 1 like 1	-10.74	0.03
ENSMUSG00000120018	6720470G18Rik	RIKEN cDNA 6720470G18 gene	-10.68	0.00
ENSMUSG00000090000	Ier3ip1	immediate early response 3 interacting protein 1	-10.62	0.00
ENSMUSG00000031465	Angpt2	angiopoietin 2	-10.60	0.00
ENSMUSG00000021241	Isca2	iron-sulfur cluster assembly 2	-10.52	0.00
ENSMUSG00000035354	Uvrag	UV radiation resistance associated	-10.35	0.00
ENSMUSG00000114230	Gm48239	predicted gene, 48239	-10.27	0.04
ENSMUSG00000046056	Sbsn	suprabasin	-10.18	0.02
ENSMUSG00000085440	Sorbs2os	sorbin and SH3 domain containing 2, opposite strand	-10.00	0.00
ENSMUSG00000120315	Gm57455	predicted gene, 57455	-9.98	0.00
ENSMUSG00000097462	9530026P05Rik	RIKEN cDNA 9530026P05 gene	-9.95	0.00
ENSMUSG00000032338	Hcn4	hyperpolarization activated cyclic nucleotide gated potassium channel 4	-9.92	0.00

Essemble ID	Gene Symbol	Gene Description	Fold Change	P Value
ENSMUSG00000010660	Plcd1	phospholipase C delta 1	-9.88	0.00
ENSMUSG00000034871	Fam151a	family with sequence similarity 151 member A	-9.81	0.00
ENSMUSG00000026073	Il1r2	interleukin 1 receptor type 2	-9.78	0.00
ENSMUSG00000097910	Hdnr	Hand2 downstream lncRNA	-9.77	0.00
ENSMUSG00000086430	4930551O13Rik	RIKEN cDNA 4930551O13 gene	-9.70	0.00
ENSMUSG00000113701	B230303A05Rik	RIKEN cDNA B230303A05 gene	-9.59	0.00
ENSMUSG00000074467	Gm10702	predicted gene 10702	-9.53	0.00
ENSMUSG00002075214	Gm56421	predicted gene, 43923	-9.44	0.00
ENSMUSG00000025496	Drd4	dopamine receptor D4	-9.43	0.00
ENSMUSG00000056174	Col8a2	collagen type VIII alpha 2 chain	-9.36	0.00
ENSMUSG00000039476	Prrx2	paired related homeobox 2	-9.30	0.00
ENSMUSG00000042988	Notum	notum, palmitoleoyl-protein carboxylesterase	-9.29	0.00
ENSMUSG00000099762	Gm21149	predicted gene 10471	-9.23	0.00
ENSMUSG00000087496	Gm13523	predicted gene 13523	-9.13	0.00
ENSMUSG00000033960	Jcad	junctional cadherin 5 associated	-9.10	0.00
ENSMUSG00000054091	1810037I17Rik	RIKEN cDNA 1810037I17 gene	-9.02	0.00
ENSMUSG00000118314	1700001K23Rik	RIKEN cDNA 1700001K23 gene	-8.93	0.00
ENSMUSG00000074628	Tldc2	TBC/LysM-associated domain containing 2	-8.89	0.00
ENSMUSG00000108256	Gm43923	predicted gene, 43923	-8.82	0.00
ENSMUSG00002076121	Gm56420	predicted gene, 56420	-8.80	0.00
ENSMUSG00000104507	A430027H14Rik	RIKEN cDNA A430027H14 gene	-8.76	0.00
ENSMUSG00000118101	F730048M01Rik	RIKEN cDNA F730048M01 gene	-8.72	0.00
ENSMUSG00000087261	Gm15477	predicted gene 15477	-8.70	0.00
ENSMUSG00000097139	Gm26626	predicted gene, 26626	-8.68	0.00
ENSMUSG00000103136	Gm37980	predicted gene, 37980	-8.65	0.01
ENSMUSG00000108691	Gm45226	predicted gene 45226	-8.63	0.00
ENSMUSG00000027820	Mme	membrane metalloendopeptidase	-8.62	0.00
ENSMUSG00000011263	Exoc3l2	exocyst complex component 3 like 2	-8.58	0.01
ENSMUSG00000064845	Gm26203	predicted gene, 26203	-8.56	0.00
ENSMUSG00000025348	Itga7	integrin subunit alpha 7	-8.48	0.00
ENSMUSG00000090427	Gm17225	predicted gene 17225	-8.44	0.00
ENSMUSG00000025144	Cenpx	centromere protein X	-8.35	0.00
ENSMUSG00000113334	D030007L05Rik	RIKEN cDNA D030007L05 gene	-8.35	0.00
ENSMUSG00000102909	Gm2453	predicted gene 2453	-8.28	0.00
ENSMUSG00000113804	Gm46424	predicted gene, 46424	-8.20	0.00
ENSMUSG00000021669	Cert1	ceramide transporter 1	-8.14	0.00
ENSMUSG00000105579	Gm43251	predicted gene 43251	-8.11	0.00
ENSMUSG00000109785	Gm45542	predicted gene 45542	-8.08	0.00
ENSMUSG00000014243	Zswim7	zinc finger SWIM-type containing 7	-8.03	0.00
ENSMUSG00000087607	Gm16034	predicted gene 16034	-7.98	0.00
ENSMUSG00000105260	Gm40040	predicted gene, 40040	-7.94	0.00
ENSMUSG00000044276	4933427E11Rik	RIKEN cDNA 4933427E11 gene	-7.91	0.00
ENSMUSG00000109307	Gm44700	predicted gene 44700	-7.77	0.00
ENSMUSG00000110674	Gm45892	predicted gene 45892	-7.76	0.00
ENSMUSG00000025578	Cbx8	chromobox 8	-7.73	0.00
ENSMUSG00000035383	Pmch	pro-melanin concentrating hormone	-7.68	0.00

Essemble ID	Gene Symbol	Gene Description	Fold Change	P Value
ENSMUSG00000063130	Calml3	calmodulin like 3	-7.56	0.00
ENSMUSG00000031461	Myom2	myomesin 2	-7.55	0.00
ENSMUSG00000026622	Nek2	NIMA related kinase 2	-7.53	0.00
ENSMUSG00000084948	1700061H18Rik	RIKEN cDNA 1700061H18 gene	-7.50	0.00
ENSMUSG00000033060	Lmo7	LIM domain 7	-7.50	0.00
ENSMUSG00000054555	Adam12	ADAM metallopeptidase domain 12	-7.48	0.00
ENSMUSG00000041020	Map7d2	MAP7 domain containing 2	-7.48	0.00
ENSMUSG00000104515	Gm37163	predicted gene, 37163	-7.45	0.00
ENSMUSG00000086998	Gm16033	predicted gene 16033	-7.40	0.00
ENSMUSG00000097320	Tmem147os	transmembrane protein 147, opposite strand	-7.37	0.00
ENSMUSG00000056718	Gm13199	predicted gene 13199	-7.34	0.00
ENSMUSG00000037679	Inf2	inverted formin 2	-7.28	0.00
ENSMUSG00000105945	Gm43570	predicted gene 43570	-7.26	0.00
ENSMUSG00000001524	Gtf2h4	general transcription factor IIH subunit 4	-7.25	0.01
ENSMUSG00000073725	Lmbrd1	LMBR1 domain containing 1	-7.24	0.01
ENSMUSG00000040387	Klhl32	kelch like family member 32	-7.24	0.00
ENSMUSG00000086544	Chn1os3	chimerin 1, opposite strand 3	-7.23	0.00
ENSMUSG00000079442	St6galnac4	ST6 N-acetylgalactosaminide alpha-2,6-sialyltransferase 4	-7.20	0.00
ENSMUSG00000041313	Slc7a1	solute carrier family 7 member 1	-7.18	0.00
ENSMUSG00000089812	Gm15867	predicted gene 15867	-7.18	0.00
ENSMUSG00000019989	Enpp3	ectonucleotide pyrophosphatase/phosphodiesterase 3	-7.16	0.00
ENSMUSG00000027326	Knl1	kinetochore scaffold 1	-7.16	0.02
ENSMUSG00000120408	Gm56955	predicted gene, 56955	-7.15	0.00
ENSMUSG00000085363	Gm15478	predicted gene 15478	-7.15	0.00
ENSMUSG00000039492	Ccdc27	coiled-coil domain containing 27	-7.12	0.00
ENSMUSG00000120868	Gm56800	predicted gene, 56800	-7.07	0.00
ENSMUSG00000113029	Gm40578	predicted gene, 40578	-7.04	0.00
ENSMUSG00000103843	Gm31266	predicted gene, 31266	-6.98	0.00
ENSMUSG00000099655	2310034G01Rik	RIKEN cDNA 2310034G01 gene	-6.92	0.00
ENSMUSG00000086796	4932702P03Rik	RIKEN cDNA 4932702P03 gene	-6.87	0.00
ENSMUSG00000107745	Gm43917	predicted gene, 43917	-6.84	0.00
ENSMUSG00000084986	Gm16224	predicted gene 16224	-6.81	0.01
ENSMUSG00000058571	Gpc6	glypican 6	-6.79	0.00
ENSMUSG00000040797	Iqsec3	IQ motif and Sec7 domain ArfGEF 3	-6.74	0.00
ENSMUSG00000074346	Kcnd3os	potassium voltage-gated channel, Shal-related family, member 3, opposite strand	-6.67	0.00
ENSMUSG00000005973	Rcn1	reticulocalbin 1	-6.65	0.00
ENSMUSG00000119968	D530017H19Rik	RIKEN cDNA D530017H19 gene	-6.64	0.00
ENSMUSG00000113898	Gm19144	synaptotagmin binding, cytoplasmic RNA interacting protein pseudogene	-6.64	0.00
ENSMUSG00000032305	Fam219b	family with sequence similarity 219 member B	-6.61	0.00
ENSMUSG00000036246	Gmip	GEM interacting protein	-6.47	0.00
ENSMUSG00000038193	Hand2	heart and neural crest derivatives expressed 2	-6.42	0.00
ENSMUSG00000006731	B4galnt1	beta-1,4-N-acetyl-galactosaminyltransferase 1	-6.40	0.00
ENSMUSG00000032625	Thsd7a	thrombospondin type 1 domain containing 7A	-6.38	0.00
ENSMUSG00000114833	Gm41192	predicted gene, 41192	-6.37	0.00
ENSMUSG00000034739	Mfrp	membrane frizzled-related protein	-6.37	0.00
ENSMUSG00000079462	Gm15737	predicted gene 15737	-6.36	0.00

Essemble ID	Gene Symbol	Gene Description	Fold Change	P Value
ENSMUSG00000036615	Rfxap	regulatory factor X associated protein	-6.31	0.00
ENSMUSG00000046179	E2f8	E2F transcription factor 8	-6.31	0.00
ENSMUSG00000086389	Gm15998	predicted gene 15998	-6.28	0.00
ENSMUSG00000075162	Gm10812	predicted gene 10812	-6.27	0.00
ENSMUSG00000090626	Tex9	testis expressed 9	-6.25	0.00
ENSMUSG00000020427	Igfbp3	insulin like growth factor binding protein 3	-6.23	0.00
ENSMUSG00000029674	Limk1	LIM domain kinase 1	-6.21	0.00
ENSMUSG00000022621	Rabl2	RAB, member of RAS oncogene family like 2A	-6.18	0.01
ENSMUSG00000046994	Mars2	methionyl-tRNA synthetase 2, mitochondrial	-6.13	0.01
ENSMUSG00000087601	Uchl1os	ubiquitin carboxy-terminal hydrolase L1, opposite strand	-6.12	0.00
ENSMUSG00000013629	Cad	carbamoyl-phosphate synthetase 2, aspartate transcarbamylase, and dihydroorotase	-6.11	0.00
ENSMUSG00000000594	Gm2a	ganglioside GM2 activator	-6.10	0.00
ENSMUSG00000114117	Gm48795	predicted gene, 48795	-6.07	0.00
ENSMUSG00000097404	Gm10814	predicted gene 10814	-6.04	0.00
ENSMUSG00000114088	Gm47898	predicted gene, 47898	-6.03	0.00
ENSMUSG00000072980	Oip5	Opa interacting protein 5	-6.03	0.00
ENSMUSG00000018893	Mb	myoglobin	-6.02	0.00
ENSMUSG00000030275	Etnk1	ethanolamine kinase 1	-6.01	0.00
ENSMUSG00000101625	Gm29371	predicted gene 29371	-5.97	0.02
ENSMUSG00000027082	Tfpi	tissue factor pathway inhibitor	-5.95	0.01
ENSMUSG00000111639	1700019L13Rik	RIKEN cDNA 1700019L13 gene	-5.91	0.00
ENSMUSG00000047759	Hs3st3a1	heparan sulfate-glucosamine 3-sulfotransferase 3A1	-5.89	0.00
ENSMUSG00002076326	Gm55134	predicted gene, 55134	-5.88	0.00
ENSMUSG00000116508	Gm49463	predicted gene, 49463	-5.86	0.00
ENSMUSG00000086629	2810403D21Rik	RIKEN cDNA 2810403D21 gene	-5.85	0.00
ENSMUSG00000109602	Gm31597	predicted gene, 31597	-5.85	0.00
ENSMUSG00000097466	D430036J16Rik	RIKEN cDNA D430036J16 gene	-5.79	0.00
ENSMUSG00000038347	Tcte2	t-complex-associated testis expressed 2	-5.78	0.00
ENSMUSG00000052928	Ctif	cap binding complex dependent translation initiation factor	-5.77	0.00
ENSMUSG00000109293	Dcst2	DC-STAMP domain containing 2	-5.75	0.00
ENSMUSG00000107802	1700126G02Rik	RIKEN cDNA 1700126G02 gene	-5.75	0.00
ENSMUSG00000040945	Rcc2	regulator of chromosome condensation 2	-5.74	0.00
ENSMUSG00000108617	Gm31749	predicted gene, 31749	-5.71	0.00
ENSMUSG00000020877	Scrn2	secernin 2	-5.68	0.04
ENSMUSG00000110696	Gm45706	predicted gene 45706	-5.67	0.00
ENSMUSG00000058192	Zfp846	zinc finger protein 846	-5.65	0.00
ENSMUSG00000120820	1700031C06Rik	RIKEN cDNA 1700031C06 gene	-5.63	0.00
ENSMUSG00000022883	Robo1	roundabout guidance receptor 1	-5.62	0.00
ENSMUSG00000085526	Gm16083	predicted gene 16083	-5.61	0.00
ENSMUSG00000087466	A330041J22Rik	RIKEN cDNA A330041J22 gene	-5.57	0.00
ENSMUSG00000102840	Gm38037	predicted gene, 38037	-5.56	0.00
ENSMUSG00000032042	Srpr	SRP receptor subunit alpha	-5.56	0.00
ENSMUSG00000113909	Gm36377	predicted gene, 36377	-5.52	0.00
ENSMUSG00000050010	Shisa3	shisa family member 3	-5.50	0.00
ENSMUSG00000079175	Gm11060	predicted gene 11060	-5.46	0.00
ENSMUSG00000112698	Gm7525	cofilin-1 pseudogene	-5.45	0.00

Essemble ID	Gene Symbol	Gene Description	Fold Change	P Value
ENSMUSG00000022474	Pmm1	phosphomannomutase 1	-5.40	0.00
ENSMUSG00000075229	Mix23	mitochondrial matrix import factor 23	-5.40	0.00
ENSMUSG00000113224	Gm48444	predicted gene, 48444	-5.40	0.01
ENSMUSG00000085192	Gm12195	predicted gene 12195	-5.35	0.05
ENSMUSG00000005813	Metap1	methionyl aminopeptidase 1	-5.34	0.01
ENSMUSG00000022701	Ccdc191	coiled-coil domain containing 191	-5.32	0.00
ENSMUSG00000019970	Sgk1	serum/glucocorticoid regulated kinase 1	-5.29	0.05
ENSMUSG00000048636	A730049H05Rik	RIKEN cDNA A730049H05 gene	-5.28	0.00
ENSMUSG00000025607	Copg2	COPI coat complex subunit gamma 2	-5.27	0.00
ENSMUSG00000045045	Lrfn4	leucine rich repeat and fibronectin type III domain containing 4	-5.27	0.00
ENSMUSG00000021508	Cxcl14	C-X-C motif chemokine ligand 14	-5.26	0.00
ENSMUSG00000103009	Gm56350	predicted gene, 56350	-5.24	0.00
ENSMUSG00000021109	Hif1a	hypoxia inducible factor 1 subunit alpha	-5.22	0.00
ENSMUSG00000017299	Dnttip1	deoxynucleotidyltransferase terminal interacting protein 1	-5.19	0.00
ENSMUSG00000120858	Gm56992	predicted gene, 56992	-5.18	0.00
ENSMUSG00000107597	Gm44764	predicted gene 44764	-5.15	0.01
ENSMUSG00000073656	Gm10558	predicted gene 10558	-5.14	0.00
ENSMUSG00000074987	Wt1os	WT1 transcription factor, opposite strand	-5.11	0.00
ENSMUSG00000086792	Gm12364	predicted gene 12364	-5.09	0.00
ENSMUSG00000119980	Gm57064	predicted gene, 57064	-5.08	0.00
ENSMUSG00000028099	Polr3c	RNA polymerase III subunit C	-5.07	0.03
ENSMUSG00000016458	Wt1	WT1 transcription factor	-5.03	0.00
ENSMUSG00000024658	Gm9750	predicted gene 9750	-5.00	0.00
ENSMUSG00000075029	4930558J22Rik	RIKEN cDNA 4930558J22 gene	-4.98	0.00
ENSMUSG00000034613	Ppm1h	protein phosphatase, Mg2+/Mn2+ dependent 1H	-4.98	0.00
ENSMUSG00000071398	2410004P03Rik	chromosome 2 open reading frame 50	-4.97	0.01
ENSMUSG00000098121	Gm27038	predicted gene, 27038	-4.96	0.00
ENSMUSG00000092286	Dtnbos	dystrobrevin, beta, opposite strand	-4.94	0.00
ENSMUSG00000053646	Plxnb1	plexin B1	-4.91	0.00
ENSMUSG00000106086	Lef1os1	LEF1 opposite strand RNA 1	-4.91	0.00
ENSMUSG00000028007	Snx7	sorting nexin 7	-4.91	0.00
ENSMUSG00000107546	Gm20560	predicted gene, 20560	-4.90	0.00
ENSMUSG00000105813	Gm42928	predicted gene 42928	-4.90	0.00
ENSMUSG00000001065	Zfp276	zinc finger protein 276	-4.88	0.00
ENSMUSG00000014353	Tmem87b	transmembrane protein 87B	-4.85	0.00
ENSMUSG00000079076	Gm3086	RuvB-like protein 1 pseudogene	-4.84	0.00
ENSMUSG00000043782	Bicdl2	BICD family like cargo adaptor 2	-4.82	0.00
ENSMUSG00000082870	Gm12165	predicted gene 12165	-4.81	0.00
ENSMUSG00000038371	Sbf2	SET binding factor 2	-4.81	0.00
ENSMUSG00000050141	Spata31f3	spermatogenesis associated 31 subfamily F member 3	-4.80	0.00
ENSMUSG00000024247	Pkdcc	protein kinase domain containing, cytoplasmic	-4.79	0.00
ENSMUSG00000036856	Wnt4	Wnt family member 4	-4.77	0.00
ENSMUSG00000062044	Lmtk3	lemur tyrosine kinase 3	-4.77	0.00
ENSMUSG00000044006	Cilp2	cartilage intermediate layer protein 2	-4.76	0.00
ENSMUSG00000038598	Shoc1	shortage in chiasmata 1	-4.75	0.01
ENSMUSG00000072589	Gm10371	predicted gene 10371	-4.75	0.00

Essemble ID	Gene Symbol	Gene Description	Fold Change	P Value
ENSMUSG00002075463	Gm56190	predicted gene, 56190	-4.74	0.00
ENSMUSG00000061559	Skic8	SKI8 subunit of superkiller complex	-4.72	0.00
ENSMUSG00000050953	Gja1	gap junction protein alpha 1	-4.71	0.00
ENSMUSG00000006463	Zdhhc24	zinc finger DHHC-type containing 24	-4.70	0.01
ENSMUSG00000022847	Thpo	thrombopoietin	-4.68	0.00
ENSMUSG00000069763	Tmem100	transmembrane protein 100	-4.67	0.00
ENSMUSG00000042564	Fam227a	family with sequence similarity 227 member A	-4.64	0.05
ENSMUSG00000040896	Kcnd3	potassium voltage-gated channel subfamily D member 3	-4.62	0.00
ENSMUSG00000073073	Gm8098	predicted gene 8098	-4.62	0.00
ENSMUSG00000022537	Tmem44	transmembrane protein 44	-4.62	0.00
ENSMUSG00000049985	Ankrd55	ankyrin repeat domain 55	-4.61	0.00
ENSMUSG00000097847	4930478K11Rik	RIKEN cDNA 4930478K11 gene	-4.61	0.00
ENSMUSG00000073394	Runx2os1	runt related transcription factor 2, opposite strand 1	-4.61	0.00
ENSMUSG00000040738	Ints8	integrator complex subunit 8	-4.59	0.00
ENSMUSG00000090150	Acad11	acyl-CoA dehydrogenase family member 11	-4.59	0.00
ENSMUSG00000120997	Gm57385	predicted gene, 57385	-4.58	0.00
ENSMUSG00000024889	Rce1	Ras converting CAAX endopeptidase 1	-4.58	0.00
ENSMUSG00000078240	Gm3550	ribosomal protein L29, retrotransposed	-4.57	0.00
ENSMUSG00000033781	Asb13	ankyrin repeat and SOCS box containing 13	-4.57	0.00
ENSMUSG00000026792	Lrsam1	leucine rich repeat and sterile alpha motif containing 1	-4.55	0.00
ENSMUSG00000029319	Coq2	coenzyme Q2, polyprenyltransferase	-4.54	0.00
ENSMUSG00000113491	Gm19221	predicted gene, 19221	-4.50	0.00
ENSMUSG00000068600	Gml2	glycosylphosphatidylinositol anchored molecule like	-4.50	0.00
ENSMUSG00000054061	Gm9934	predicted gene 9934	-4.49	0.00
ENSMUSG00000087109	4930474H06Rik	RIKEN cDNA 4930474H06 gene	-4.48	0.00
ENSMUSG00000117309	Gm49960	predicted gene, 49960	-4.48	0.00
ENSMUSG00000087528	9830144P21Rik	RIKEN cDNA 9830144P21 gene	-4.48	0.00
ENSMUSG00000042708	Shcbp1l	SHC binding and spindle associated 1 like	-4.46	0.00
ENSMUSG00000071359	Tbpl1	TATA-box binding protein like 1	-4.44	0.00
ENSMUSG00000028688	Toe1	target of EGR1, exonuclease	-4.42	0.00
ENSMUSG00000047696	Ccdc144b	coiled-coil domain containing 144B	-4.41	0.00
ENSMUSG00000045275	Lca5l	lebercilin LCA5 like	-4.40	0.00
ENSMUSG00000012296	Tjap1	tight junction associated protein 1	-4.37	0.00
ENSMUSG00000030577	Cd22	CD22 molecule	-4.37	0.00
ENSMUSG00000055972	2810407A14Rik	RIKEN cDNA 2810407A14 gene	-4.36	0.00
ENSMUSG00000029153	Ociad2	OCIA domain containing 2	-4.33	0.01
ENSMUSG00000075602	Ly6a	lymphocyte antigen 6 family member A	-4.33	0.00
ENSMUSG00000112388	AI463170	expressed sequence AI463170	-4.31	0.00
ENSMUSG00000025644	Gm7628	predicted gene 7628	-4.31	0.00
ENSMUSG00000024530	Prelid3a	PRELI domain containing 3A	-4.30	0.00
ENSMUSG00000048668	Rhno1	RAD9-HUS1-RAD1 interacting nuclear orphan 1	-4.28	0.00
ENSMUSG00000085023	Gm12744	predicted gene 12744	-4.27	0.00
ENSMUSG00000028011	Tdo2	tryptophan 2,3-dioxygenase	-4.25	0.00
ENSMUSG00000025027	Xpnpep1	X-prolyl aminopeptidase 1	-4.24	0.00
ENSMUSG00000121075	Gm56727	predicted gene, 56727	-4.23	0.00
ENSMUSG00000098482	Mir6363	microRNA 6363	-4.23	0.00

Essemble ID	Gene Symbol	Gene Description	Fold Change	P Value
ENSMUSG00000093650	Gm20631	predicted gene 20631	-4.23	0.00
ENSMUSG00002075808	Gm55497	predicted gene, 55497	-4.22	0.00
ENSMUSG00000056427	Slit3	slit guidance ligand 3	-4.21	0.00
ENSMUSG00000114922	Ppifos	peptidylprolyl isomerase F, opposite strand	-4.21	0.00
ENSMUSG00000035305	Ror1	receptor tyrosine kinase like orphan receptor 1	-4.21	0.00
ENSMUSG00000034321	Exosc1	exosome component 1	-4.20	0.03
ENSMUSG00000023147	Get1	guided entry of tail-anchored proteins factor 1	-4.19	0.00
ENSMUSG00000011256	Adam19	ADAM metallopeptidase domain 19	-4.18	0.00
ENSMUSG00000030671	Pde3b	phosphodiesterase 3B	-4.17	0.00
ENSMUSG00000021385	Ippk	inositol-pentakisphosphate 2-kinase	-4.17	0.00
ENSMUSG00000006390	Elovl1	ELOVL fatty acid elongase 1	-4.16	0.00
ENSMUSG00000112894	Gm47009	predicted gene, 47009	-4.15	0.00
ENSMUSG00000067973	Gm6994	predicted gene 6994	-4.14	0.00
ENSMUSG00000002227	Mov10	Mov10 RISC complex RNA helicase	-4.14	0.02
ENSMUSG00000021281	Tnfaip2	TNF alpha induced protein 2	-4.14	0.00
ENSMUSG00000062410	Hsd3b3	hydroxy-delta-5-steroid dehydrogenase, 3 beta- and steroid delta-isomerase 1	-4.13	0.00
ENSMUSG00000107402	4732416N19Rik	RIKEN cDNA 4732416N19 gene	-4.13	0.00
ENSMUSG00000087355	Gm13187	predicted gene 13187	-4.11	0.00
ENSMUSG00000093803	Ppp2r3d	protein phosphatase 2 (formerly 2A), regulatory subunit B'', delta	-4.11	0.00
ENSMUSG00000032312	Csk	C-terminal Src kinase	-4.10	0.00
ENSMUSG00000070871	Ccnyl1	cyclin Y like 1	-4.10	0.00
ENSMUSG00000028860	Sytl1	synaptotagmin like 1	-4.10	0.00
ENSMUSG00000036334	Igsf10	immunoglobulin superfamily member 10	-4.08	0.00
ENSMUSG00000105063	Gm43488	predicted gene 43488	-4.08	0.00
ENSMUSG00000084968	Gm12743	predicted gene 12743	-4.05	0.00
ENSMUSG00000044674	Fzd1	frizzled class receptor 1	-4.04	0.00
ENSMUSG00000118578	1700014B07Rik	RIKEN cDNA 1700014B07 gene	-4.03	0.00
ENSMUSG00000121132	Gm52182	predicted gene, 52182	-4.02	0.00
ENSMUSG00000028703	Lrrc41	leucine rich repeat containing 41	-4.00	0.00
ENSMUSG00000086427	Hoxa11os	homeobox A11, opposite strand	-4.00	0.00
ENSMUSG00000062074	Ccn6	cellular communication network factor 6	-3.98	0.00
ENSMUSG00000026584	Scyl3	SCY1 like pseudokinase 3	-3.97	0.00
ENSMUSG00000045095	Magi1	membrane associated guanylate kinase, WW and PDZ domain containing 1	-3.97	0.00
ENSMUSG00000120661	Gm57121	predicted gene 43488	-3.97	0.00
ENSMUSG00000025026	Add3	adducin 3	-3.95	0.00
ENSMUSG00000117427	Gm36199	predicted gene, 36199	-3.94	0.00
ENSMUSG00000112259	Gm17980	WD repeat domain 75 pseudogene	-3.93	0.00
ENSMUSG00000048376	F2r	coagulation factor II thrombin receptor	-3.93	0.02
ENSMUSG00000038210	Hoxa11	homeobox A11	-3.92	0.00
ENSMUSG00000108688	Gm44985	predicted gene, 57121	-3.90	0.00
ENSMUSG00000085830	Grin1os	glutamate receptor, ionotropic, NMDA1 (zeta 1), opposite strand	-3.89	0.00
ENSMUSG00000085996	A830012C17Rik	RIKEN cDNA A830012C17 gene	-3.87	0.00
ENSMUSG00000086196	Gm13571	predicted gene 13571	-3.87	0.00
ENSMUSG00000085083	Gm11615	predicted gene 11615	-3.87	0.00
ENSMUSG00000108934	Gm44732	predicted gene 44732	-3.86	0.00
ENSMUSG00000049928	Glp2r	glucagon like peptide 2 receptor	-3.84	0.00

Essemble ID	Gene Symbol	Gene Description	Fold Change	P Value
ENSMUSG00000042286	Stab1	stabilin 1	-3.83	0.00
ENSMUSG00000048617	Rtbdn	retbindin	-3.83	0.00
ENSMUSG00000116858	Gm49797	predicted gene, 49797	-3.81	0.00
ENSMUSG00000042451	Mybph	myosin binding protein H	-3.81	0.00
ENSMUSG00000098292	Gm27194	predicted gene 27194	-3.80	0.00
ENSMUSG00000053615	Gm9913	predicted gene 9913	-3.79	0.00
ENSMUSG00000063576	Klhdc3	kelch domain containing 3	-3.79	0.00
ENSMUSG00000024587	Nars1	asparaginyl-tRNA synthetase 1	-3.79	0.01
ENSMUSG00000037428	Vgf	VGF nerve growth factor inducible	-3.78	0.02
ENSMUSG00000021963	Sap18	Sin3A associated protein 18	-3.77	0.00
ENSMUSG00000021066	Atl1	atlastin GTPase 1	-3.77	0.00
ENSMUSG00000087036	Gm11748	predicted gene 11748	-3.76	0.00
ENSMUSG00000035798	Zdhhc17	zinc finger DHHC-type palmitoyltransferase 17	-3.75	0.00
ENSMUSG00000043059	Zfp513	zinc finger protein 513	-3.75	0.00
ENSMUSG00000025817	Nudt5	nudix hydrolase 5	-3.74	0.00
ENSMUSG00000031762	Mt2	metallothionein 2	-3.74	0.00
ENSMUSG00000097226	Gm26600	predicted gene, 26600	-3.74	0.00
ENSMUSG00000105104	Gm43729	predicted gene 43729	-3.74	0.00
ENSMUSG00000121235	Gm57252	predicted gene, 57252	-3.73	0.00
ENSMUSG00000032401	Lctl	lactase like	-3.72	0.00
ENSMUSG00000108103	Gm44081	predicted gene, 44081	-3.72	0.00
ENSMUSG00000100209	Gm28793	predicted gene 28793	-3.71	0.00
ENSMUSG00000048277	Syngr2	synaptogyrin 2	-3.71	0.00
ENSMUSG00000099874	Gm29629	predicted gene 29629	-3.68	0.01
ENSMUSG00000029591	Ung	uracil DNA glycosylase	-3.66	0.00
ENSMUSG00000019139	Isyna1	inositol-3-phosphate synthase 1	-3.66	0.01
ENSMUSG00000038271	Iffo1	intermediate filament family orphan 1	-3.65	0.00
ENSMUSG00000069136	A130006I12Rik	RIKEN cDNA A130006I12 gene	-3.64	0.00
ENSMUSG00000034349	Smc4	structural maintenance of chromosomes 4	-3.63	0.00
ENSMUSG00000066057	Gm1976	predicted gene 1976	-3.63	0.00
ENSMUSG00000021807	Rtraf	RNA transcription, translation and transport factor	-3.63	0.00
ENSMUSG00000082283	Gm14472	predicted gene 14472	-3.63	0.01
ENSMUSG00000078722	Gm12394	spermatogenesis associated 31 subfamily F member 1E	-3.62	0.00
ENSMUSG00000027714	Exosc9	exosome component 9	-3.62	0.00
ENSMUSG00000085818	Gm13267	predicted gene 13267	-3.61	0.00
ENSMUSG00000097986	Gm26953	predicted gene, 26953	-3.61	0.00
ENSMUSG00000104786	Gm43573	predicted gene 43573	-3.61	0.00
ENSMUSG00000104043	Gm6525	ribosomal protein L36a pseudogene	-3.61	0.03
ENSMUSG00000027985	Lef1	lymphoid enhancer binding factor 1	-3.60	0.00
ENSMUSG00000036867	Smad6	SMAD family member 6	-3.60	0.00
ENSMUSG00000017485	Top2b	DNA topoisomerase II beta	-3.59	0.00
ENSMUSG00000021286	Zfyve21	zinc finger FYVE-type containing 21	-3.59	0.00
ENSMUSG00000015668	Pdzd11	PDZ domain containing 11	-3.59	0.00
ENSMUSG00000086103	Gm11832	predicted gene 11832	-3.58	0.00
ENSMUSG00000101674	4930444A19Rik	RIKEN cDNA 4930444A19 gene	-3.58	0.00
ENSMUSG00000035936	Aldh5a1	aldehyde dehydrogenase 5 family member A1	-3.58	0.00

Essemble ID	Gene Symbol	Gene Description	Fold Change	P Value
ENSMUSG00000108574	Gm44981	predicted gene 44981	-3.57	0.00
ENSMUSG00000068036	Afdn	afadin, adherens junction formation factor	-3.57	0.00
ENSMUSG00000110765	Gm47468	predicted gene, 47468	-3.56	0.00
ENSMUSG00000110007	Gm45352	predicted gene 45352	-3.56	0.00
ENSMUSG00000110816	Gm19178	amyloid beta (A4) precursor protein-binding, family B, member 1 pseudogene	-3.54	0.00
ENSMUSG00000098449	Gm7467	predicted gene 7467	-3.54	0.00
ENSMUSG00000097002	Gm2670	predicted gene 2670	-3.54	0.00
ENSMUSG00000020566	Atp6v1c2	ATPase H+ transporting V1 subunit C2	-3.54	0.00
ENSMUSG00000087314	Prkag2os2	protein kinase, AMP-activated, gamma 2 non-catalytic subunit, opposite strand 2	-3.54	0.00
ENSMUSG00000024942	Capn1	calpain 1	-3.53	0.00
ENSMUSG00000032010	Usp2	ubiquitin specific peptidase 2	-3.52	0.00
ENSMUSG00000020400	Tnip1	TNFAIP3 interacting protein 1	-3.52	0.03
ENSMUSG00000033361	Prrg3	proline rich and Gla domain 3	-3.52	0.02
ENSMUSG00000093598	A730085K08Rik	RIKEN cDNA A730085K08 gene	-3.52	0.00
ENSMUSG00000028497	Hacd4	3-hydroxyacyl-CoA dehydratase 4	-3.51	0.00
ENSMUSG00000043126	D830039M14Rik	RIKEN cDNA D830039M14 gene	-3.51	0.00
ENSMUSG00000015647	Lama5	laminin subunit alpha 5	-3.48	0.00
ENSMUSG00000045238	A730035I17Rik	RIKEN cDNA A730035I17 gene	-3.48	0.00
ENSMUSG00000021676	Iqgap2	IQ motif containing GTPase activating protein 2	-3.48	0.00
ENSMUSG00000111889	Gm48071	predicted gene, 48071	-3.48	0.00
ENSMUSG00000111929	Gm48780	predicted gene, 48780	-3.46	0.00
ENSMUSG00000025739	Gng13	G protein subunit gamma 13	-3.46	0.00
ENSMUSG00000003865	Gys1	glycogen synthase 1	-3.45	0.00
ENSMUSG00000120886	Gm56584	predicted gene, 56584	-3.44	0.00
ENSMUSG00000026313	Hdac4	histone deacetylase 4	-3.44	0.01
ENSMUSG00000114345	Gm10873	predicted gene 10873	-3.43	0.00
ENSMUSG00000038225	Primpol	primase and DNA directed polymerase	-3.42	0.00
ENSMUSG00000025742	Prps2	phosphoribosyl pyrophosphate synthetase 2	-3.42	0.01
ENSMUSG00000111514	E230014E18Rik	RIKEN cDNA E230014E18 gene	-3.41	0.00
ENSMUSG00000106760	Gm42802	predicted gene 42802	-3.40	0.00
ENSMUSG00000113245	Gm48574	predicted gene, 48574	-3.40	0.00
ENSMUSG00000038523	1700003F12Rik	RIKEN cDNA 1700003F12 gene	-3.39	0.00
ENSMUSG00000120943	Gm57054	predicted gene, 57054	-3.39	0.00
ENSMUSG00000028207	Asph	aspartate beta-hydroxylase	-3.38	0.01
ENSMUSG00000110237	Gm18258	gamma-glutamyl hydrolase pseudogene	-3.38	0.01
ENSMUSG00000085523	Gm15945	predicted gene 15945	-3.37	0.00
ENSMUSG00000086709	Gm16263	predicted gene 16263	-3.36	0.00
ENSMUSG00000021400	Wrnip1	WRN helicase interacting protein 1	-3.36	0.00
ENSMUSG00000021068	Nin	ninein	-3.36	0.00
ENSMUSG00000120198	Gm4633	predicted gene 4633	-3.36	0.00
ENSMUSG00000028044	Cks1b	CDC28 protein kinase regulatory subunit 1B	-3.36	0.00
ENSMUSG00000085628	Appbp2os	amyloid beta precursor protein binding protein 2, opposite strand	-3.35	0.00
ENSMUSG00000019080	Mfsd3	major facilitator superfamily domain containing 3	-3.35	0.00
ENSMUSG00000085184	4933439K11Rik	RIKEN cDNA 4933439K11 gene	-3.35	0.00
ENSMUSG00000046378	Asphd1	aspartate beta-hydroxylase domain containing 1	-3.35	0.00
ENSMUSG00000084979	Gm16267	predicted gene 16267	-3.35	0.00

Essemble ID	Gene Symbol	Gene Description	Fold Change	P Value
ENSMUSG00000082095	Gm11991	predicted gene 11991	-3.34	0.01
ENSMUSG00000040441	Slc26a10	solute carrier family 26, member 10	-3.34	0.00
ENSMUSG00000006958	Chrd	chordin	-3.33	0.00
ENSMUSG00000099556	Gm28857	predicted gene 28857	-3.33	0.00
ENSMUSG00000070953	Rabepk	Rab9 effector protein with kelch motifs	-3.33	0.00
ENSMUSG00000116777	Zfp520-ps	zinc finger protein 520, pseudogene	-3.32	0.00
ENSMUSG00000015882	Lcorl	ligand dependent nuclear receptor corepressor like	-3.32	0.00
ENSMUSG00000033454	Zbtb1	zinc finger and BTB domain containing 1	-3.32	0.00
ENSMUSG00000106750	Gm43151	predicted gene 43151	-3.32	0.00
ENSMUSG00000064032	Gm10143	predicted gene 10143	-3.31	0.00
ENSMUSG00000027639	Samhd1	SAM and HD domain containing deoxynucleoside triphosphate triphosphohydrolase 1	-3.31	0.02
ENSMUSG00000086514	Gm11747	predicted gene 11747	-3.31	0.00
ENSMUSG00002076948	Gm55327	predicted gene, 55327	-3.31	0.00
ENSMUSG00000099879	Gm29123	predicted gene 29123	-3.29	0.00
ENSMUSG00000114708	Gm30177	predicted gene, 30177	-3.29	0.00
ENSMUSG00000097805	Gm17473	predicted gene, 17473	-3.28	0.00
ENSMUSG00000029838	Ptn	pleiotrophin	-3.27	0.00
ENSMUSG00000020962	Gtf2a1	general transcription factor IIA subunit 1	-3.27	0.00
ENSMUSG00000031765	Mt1	metallothionein 1	-3.27	0.01
ENSMUSG00000110899	Gm48840	predicted gene, 48840	-3.27	0.01
ENSMUSG00000026976	Pax8	paired box 8	-3.26	0.00
ENSMUSG00000025289	Prdx4	peroxiredoxin 4	-3.26	0.00
ENSMUSG00000119956	Gm57370	predicted gene, 57370	-3.26	0.00
ENSMUSG00000114994	Gm49097	predicted gene, 49097	-3.25	0.00
ENSMUSG00000112274	Gm47525	predicted gene, 47525	-3.25	0.00
ENSMUSG00000114515	Gm57854	predicted gene, 57854	-3.24	0.00
ENSMUSG00000031075	Ano1	anoctamin 1	-3.23	0.00
ENSMUSG00000115097	Gm49150	predicted gene, 49150	-3.23	0.00
ENSMUSG00000080783	Gm8250	predicted gene 8250	-3.22	0.00
ENSMUSG00000026970	Rbms1	RNA binding motif single stranded interacting protein 1	-3.20	0.03
ENSMUSG00000087535	Zmiz1os1	Zmiz1 opposite strand 1	-3.20	0.00
ENSMUSG00000058388	Phtf1	putative homeodomain transcription factor 1	-3.20	0.00
ENSMUSG00000121151	Gm56909	predicted gene, 56909	-3.19	0.00
ENSMUSG00000097061	9330151L19Rik	RIKEN cDNA 9330151L19 gene	-3.19	0.00
ENSMUSG00000079614	Seh1l	SEH1 like nucleoporin	-3.19	0.00
ENSMUSG00000084780	Gm15350	predicted gene 15350	-3.18	0.00
ENSMUSG00000086600	C030005K06Rik	RIKEN cDNA C030005K06 gene	-3.17	0.00
ENSMUSG00000043140	Tmem186	transmembrane protein 186	-3.17	0.00
ENSMUSG00000102145	Gm38056	predicted gene, 38056	-3.16	0.00
ENSMUSG00000114536	Gm48837	predicted gene, 48837	-3.16	0.00
ENSMUSG00000046079	Lrrc8d	leucine rich repeat containing 8 VRAC subunit D	-3.16	0.02
ENSMUSG00000053460	Ggcx	gamma-glutamyl carboxylase	-3.16	0.00
ENSMUSG00000106896	G630022F23Rik	RIKEN cDNA G630022F23 gene	-3.14	0.00
ENSMUSG00000118144	Gm50313	predicted gene, 50313	-3.14	0.00
ENSMUSG00000056457	Prl2c3	prolactin family 2, subfamily c, member 2	-3.14	0.00
ENSMUSG00000032340	Neo1	neogenin 1	-3.13	0.00

Essemble ID	Gene Symbol	Gene Description	Fold Change	P Value
ENSMUSG00000032306	Mpi	mannose phosphate isomerase	-3.13	0.00
ENSMUSG00000112150	9530018F02Rik	RIKEN cDNA 9530018F02 gene	-3.13	0.00
ENSMUSG00000033963	Fancd2os	FANCD2 opposite strand	-3.12	0.00
ENSMUSG00000036503	Rnf13	ring finger protein 13	-3.12	0.00
ENSMUSG00000064330	Pde6h	phosphodiesterase 6H	-3.12	0.00
ENSMUSG00000028555	Ttc39a	tetratricopeptide repeat domain 39A	-3.10	0.00
ENSMUSG00000001025	S100a6	S100 calcium binding protein A6	-3.10	0.03
ENSMUSG00000085403	Gm13068	predicted gene 13068	-3.09	0.00
ENSMUSG00000102817	Gm6185	solute carrier family 9, member C2 pseudogene	-3.09	0.00
ENSMUSG00000026273	Mterf4	mitochondrial transcription termination factor 4	-3.08	0.00
ENSMUSG00000026409	Pfkfb2	6-phosphofructo-2-kinase/fructose-2,6-biphosphatase 2	-3.07	0.00
ENSMUSG00000021061	Sptb	spectrin beta, erythrocytic	-3.06	0.00
ENSMUSG00000076439	Mog	myelin oligodendrocyte glycoprotein	-3.06	0.00
ENSMUSG00000115220	Gm49768	predicted gene, 49768	-3.05	0.00
ENSMUSG00000024273	2700062C07Rik	chromosome 18 open reading frame 21	-3.04	0.00
ENSMUSG00000039084	Chad	chondroadherin	-3.03	0.00
ENSMUSG00000092381	Lncob1	lncRNA osteoblastogenesis associated 1	-3.03	0.00
ENSMUSG00000085606	Gm15792	predicted gene 15792	-3.03	0.00
ENSMUSG00000035983	Gm7008	predicted gene 7008	-3.03	0.00
ENSMUSG00000046230	Vps13a	vacuolar protein sorting 13 homolog A	-3.02	0.00
ENSMUSG00000064120	Mocs1	molybdenum cofactor synthesis 1	-3.00	0.00
ENSMUSG00000026648	Dclre1c	DNA cross-link repair 1C	-2.99	0.00
ENSMUSG00000087193	Gm14820	predicted gene 14820	-2.99	0.00
ENSMUSG00000024140	Epas1	endothelial PAS domain protein 1	-2.98	0.00
ENSMUSG00000085485	Gm14024	predicted gene 14024	-2.97	0.00
ENSMUSG00000030096	Slc6a6	solute carrier family 6 member 6	-2.97	0.00
ENSMUSG00000039176	Polg	DNA polymerase gamma, catalytic subunit	-2.96	0.00
ENSMUSG00000085941	Gm11201	predicted gene 11201	-2.96	0.00
ENSMUSG00000112684	Gm47958	predicted gene, 47958	-2.96	0.00
ENSMUSG00000022596	Slurp1	secreted LY6/PLAUR domain containing 1	-2.96	0.00
ENSMUSG00000021124	Vti1b	vesicle transport through interaction with t-SNAREs 1B	-2.95	0.00
ENSMUSG00000107143	Gm6598	predicted gene 6598	-2.95	0.00
ENSMUSG00000031637	Lrp2bp	LRP2 binding protein	-2.95	0.00
ENSMUSG00000022219	Cideb	cell death inducing DFFA like effector b	-2.95	0.00
ENSMUSG00000027832	Ptx3	pentraxin 3	-2.95	0.03
ENSMUSG00000022370	Mrpl13	mitochondrial ribosomal protein L13	-2.94	0.00
ENSMUSG00000019864	Rtn4ip1	reticulon 4 interacting protein 1	-2.94	0.00
ENSMUSG00000053626	Tll1	tolloid like 1	-2.94	0.01
ENSMUSG00000042642	Flad1	flavin adenine dinucleotide synthetase 1	-2.93	0.00
ENSMUSG00000109881	Gm45507	predicted gene 45507	-2.93	0.00
ENSMUSG00000021196	Pfkp	phosphofructokinase, platelet	-2.93	0.00
ENSMUSG00000029231	Pdgfra	platelet derived growth factor receptor alpha	-2.92	0.00
ENSMUSG00000086898	Itpr3os	inositol 1,4,5-triphosphate receptor 3, opposite strand	-2.92	0.00
ENSMUSG00000055912	Tmem150a	transmembrane protein 150A	-2.92	0.00
ENSMUSG00000096992	Gm26788	predicted gene, 26788	-2.92	0.00
ENSMUSG00000027806	Tsc22d2	TSC22 domain family member 2	-2.92	0.01

Essemble ID	Gene Symbol	Gene Description	Fold Change	P Value
ENSMUSG00000109724	Gm18194	zinc finger protein 329 pseudogene	-2.91	0.00
ENSMUSG00000100916	Lhb	luteinizing hormone subunit beta	-2.91	0.00
ENSMUSG00000021270	Hsp90aa1	heat shock protein 90 alpha family class A member 1	-2.91	0.01
ENSMUSG00000103906	Tigd5	tigger transposable element derived 5	-2.91	0.00
ENSMUSG00000107810	Gm18609	Rho GTPase activating protein 20 pseudogene	-2.90	0.00
ENSMUSG00000087611	4930458D05Rik	RIKEN cDNA 4930458D05 gene	-2.90	0.00
ENSMUSG00000063334	Krr1	KRR1 small subunit processome component homolog	-2.89	0.00
ENSMUSG00000029507	Pus1	pseudouridine synthase 1	-2.89	0.00
ENSMUSG00000039153	Runx2	RUNX family transcription factor 2	-2.88	0.00
ENSMUSG00000048661	Lemd3	LEM domain containing 3	-2.88	0.04
ENSMUSG00000015889	Lta4h	leukotriene A4 hydrolase	-2.88	0.03
ENSMUSG00000035629	Rubcn	rubicon autophagy regulator	-2.87	0.01
ENSMUSG00000114246	Gm48603	predicted gene, 48603	-2.86	0.00
ENSMUSG00000108347	Gm19187	solute carrier family 1 (neutral amino acid transporter), member 5 pseudogene	-2.86	0.01
ENSMUSG00000003271	Sult2b1	sulfotransferase family 2B member 1	-2.86	0.00
ENSMUSG00000086241	4930483K19Rik	RIKEN cDNA 4930483K19 gene	-2.86	0.00
ENSMUSG00000019578	Ubxn6	UBX domain protein 6	-2.85	0.00
ENSMUSG00000003458	Ncstn	nicastrin	-2.85	0.00
ENSMUSG00000026017	Carf	calcium responsive transcription factor	-2.85	0.00
ENSMUSG00000085106	Gm16000	predicted gene 16000	-2.84	0.00
ENSMUSG00000035314	Gdpd5	glycerophosphodiester phosphodiesterase domain containing 5	-2.84	0.02
ENSMUSG00000005947	Itgae	integrin subunit alpha E	-2.84	0.00
ENSMUSG00000089742	Gm15898	predicted gene 15898	-2.83	0.00
ENSMUSG00000038239	Hrc	histidine rich calcium binding protein	-2.83	0.02
ENSMUSG00000114165	Gm47513	predicted gene, 47513	-2.83	0.00
ENSMUSG00000073471	Rsph3a	radial spoke head 3	-2.82	0.04
ENSMUSG00000115154	Gm49067	predicted gene, 49067	-2.82	0.00
ENSMUSG00000055968	Gm9988	predicted gene 9988	-2.82	0.00
ENSMUSG00000097535	Gm26592	predicted gene, 26592	-2.81	0.00
ENSMUSG00000029430	Ran	RAN, member RAS oncogene family	-2.80	0.03
ENSMUSG00002075710	Gm55820	predicted gene, 55820	-2.80	0.00
ENSMUSG00000026275	Ppp1r7	protein phosphatase 1 regulatory subunit 7	-2.80	0.01
ENSMUSG00000054770	Kctd18	potassium channel tetramerization domain containing 18	-2.80	0.00
ENSMUSG00000071073	Lrrc73	leucine rich repeat containing 73	-2.79	0.00
ENSMUSG00000101872	Gm29237	predicted gene 29237	-2.79	0.00
ENSMUSG00000107081	Gm43701	predicted gene 43701	-2.79	0.02
ENSMUSG00000045679	Slc66a3	solute carrier family 66 member 3	-2.79	0.00
ENSMUSG00000097547	B230110C06Rik	RIKEN cDNA B230110C06 gene	-2.78	0.00
ENSMUSG00000024222	Fkbp5	FKBP prolyl isomerase 5	-2.78	0.02
ENSMUSG00000022023	Wbp4	WW domain binding protein 4	-2.78	0.00
ENSMUSG00000106588	Gm17590	predicted gene, 17590	-2.78	0.00
ENSMUSG00000082107	Gm16216	predicted gene 16216	-2.77	0.01
ENSMUSG00000056121	Fez2	fasciculation and elongation protein zeta 2	-2.77	0.00
ENSMUSG00000087522	Gm371	germ cell-less homolog 1 pseudogene	-2.77	0.01
ENSMUSG00000097520	4930488L21Rik	GNAO1 antisense RNA 1	-2.77	0.00
ENSMUSG00000038506	Dcun1d2	defective in cullin neddylation 1 domain containing 2	-2.77	0.00

Essemble ID	Gene Symbol	Gene Description	Fold Change	P Value
ENSMUSG00000105784	Gm35065	predicted gene, 35065	-2.76	0.00
ENSMUSG00000085688	Gm15395	predicted gene 15395	-2.75	0.00
ENSMUSG00000034614	Pik3ip1	phosphoinositide-3-kinase interacting protein 1	-2.74	0.00
ENSMUSG00000031561	Tenm3	teneurin transmembrane protein 3	-2.74	0.01
ENSMUSG00000086259	Tmem150cos	transmembrane protein 150C, opposite strand	-2.74	0.00
ENSMUSG00000102323	Gm37790	predicted gene, 37790	-2.73	0.00
ENSMUSG00000085055	Gm15958	predicted gene 15958	-2.73	0.00
ENSMUSG00000003868	Ruvbl2	RuvB like AAA ATPase 2	-2.72	0.00
ENSMUSG00000022371	Col14a1	collagen type XIV alpha 1 chain	-2.72	0.00
ENSMUSG00000103576	Gm38188	predicted gene, 38188	-2.71	0.00
ENSMUSG00000097806	Gm6556	predicted gene 6556	-2.71	0.00
ENSMUSG00000074627	Mroh8	maestro heat like repeat family member 8	-2.71	0.00
ENSMUSG00000086900	Kcnab3os	potassium voltage-gated channel, shaker-related subfamily, beta member 3, opposite strand	-2.70	0.00
ENSMUSG00000101939	Gm28438	predicted gene 28438	-2.70	0.00
ENSMUSG00000046727	Cystm1	cysteine-rich transmembrane module containing 1	-2.70	0.01
ENSMUSG00000024422	Dhx16	DEAH-box helicase 16	-2.70	0.04
ENSMUSG00000057880	Abat	4-aminobutyrate aminotransferase	-2.69	0.00
ENSMUSG00000120833	Gm56540	predicted gene, 56540	-2.69	0.00
ENSMUSG00000020176	Grb10	growth factor receptor bound protein 10	-2.68	0.01
ENSMUSG00000053931	Cnn3	calponin 3	-2.68	0.00
ENSMUSG00000085737	Gm13442	predicted gene 13442	-2.68	0.00
ENSMUSG00000086424	Gm15569	predicted gene 15569	-2.68	0.00
ENSMUSG00000038206	Fbxo8	F-box protein 8	-2.68	0.00
ENSMUSG00000020077	Srgn	serglycin	-2.68	0.00
ENSMUSG00000087429	Gm16235	predicted gene 16235	-2.68	0.00
ENSMUSG00000026781	Acbd5	acyl-CoA binding domain containing 5	-2.68	0.00
ENSMUSG00000028437	Ubap1	ubiquitin associated protein 1	-2.68	0.00
ENSMUSG00000120525	Gm56755	predicted gene, 56755	-2.67	0.01
ENSMUSG00000085465	Gm15347	predicted gene 15347	-2.67	0.00
ENSMUSG00000047534	Mis18bp1	MIS18 binding protein 1	-2.67	0.00
ENSMUSG00000097724	Gm26850	predicted gene, 26850	-2.66	0.00
ENSMUSG00000028641	P3h1	prolyl 3-hydroxylase 1	-2.66	0.00
ENSMUSG00000038060	Dlec1	DLEC1 cilia and flagella associated protein	-2.66	0.00
ENSMUSG00000107792	Gm43914	predicted gene, 43914	-2.65	0.00
ENSMUSG00000003123	Lipe	lipase E, hormone sensitive type	-2.64	0.00
ENSMUSG00000025759	Mfsd8	major facilitator superfamily domain containing 8	-2.64	0.00
ENSMUSG00000021671	Poc5	POC5 centriolar protein	-2.64	0.05
ENSMUSG00000042104	Uggt2	UDP-glucose glycoprotein glucosyltransferase 2	-2.64	0.00
ENSMUSG00000087004	Gm14154	predicted gene 14154	-2.63	0.03
ENSMUSG00000029028	Lrrc47	leucine rich repeat containing 47	-2.63	0.00
ENSMUSG00000000628	Hk2	hexokinase 2	-2.63	0.05
ENSMUSG00000024074	Crim1	cysteine rich transmembrane BMP regulator 1	-2.63	0.02
ENSMUSG00000034212	Ankmy1	ankyrin repeat and MYND domain containing 1	-2.62	0.00
ENSMUSG00000028468	Rgp1	RGP1 homolog, RAB6A GEF complex partner 1	-2.62	0.02
ENSMUSG00000090207	4930524O07Rik	RIKEN cDNA 4930524O07 gene	-2.62	0.00
ENSMUSG00000026478	Lamc1	laminin subunit gamma 1	-2.62	0.00

Essemble ID	Gene Symbol	Gene Description	Fold Change	P Value
ENSMUSG00000109781	Gm45509	predicted gene 45509	-2.62	0.00
ENSMUSG00000031990	Jam3	junctional adhesion molecule 3	-2.62	0.00
ENSMUSG00000106777	Gm43150	predicted gene 43150	-2.61	0.00
ENSMUSG00000042606	Hirip3	HIRA interacting protein 3	-2.61	0.00
ENSMUSG00000120161	Gm56510	predicted gene, 56510	-2.61	0.00
ENSMUSG00000032327	Stra6	signaling receptor and transporter of retinol STRA6	-2.61	0.02
ENSMUSG00000037664	Cdkn1c	cyclin dependent kinase inhibitor 1C	-2.61	0.00
ENSMUSG00000030357	Fkbp4	FKBP prolyl isomerase 4	-2.61	0.00
ENSMUSG00000046338	Gpat2	glycerol-3-phosphate acyltransferase 2, mitochondrial	-2.61	0.00
ENSMUSG00000021981	Cab39l	calcium binding protein 39 like	-2.61	0.00
ENSMUSG00000029203	Ube2k	ubiquitin conjugating enzyme E2 K	-2.61	0.02
ENSMUSG00000041594	Tmtc4	transmembrane O-mannosyltransferase targeting cadherins 4	-2.60	0.00
ENSMUSG00000049323	Smcr8	SMCR8-C9orf72 complex subunit	-2.60	0.00
ENSMUSG00000105860	Gm42578	predicted gene 42578	-2.60	0.05
ENSMUSG00000051390	Zbtb22	zinc finger and BTB domain containing 22	-2.58	0.00
ENSMUSG00000024944	Arl2	ADP ribosylation factor like GTPase 2	-2.58	0.02
ENSMUSG00000089994	Gm16239	predicted gene 16239	-2.58	0.02
ENSMUSG00000103613	Gm34882	predicted gene, 34882	-2.58	0.00
ENSMUSG00000101172	Gm28535	predicted gene 28535	-2.58	0.00
ENSMUSG00000107432	Gm36189	predicted gene, 36189	-2.58	0.00
ENSMUSG00000112443	Gm19007	sulfotransferase family, cytosolic, 1C, member 2 pseudogene	-2.57	0.01
ENSMUSG00000086083	Gm15829	predicted gene 15829	-2.57	0.00
ENSMUSG00000061878	Sphk1	sphingosine kinase 1	-2.57	0.01
ENSMUSG00000046413	Irx3os	iroquois homeobox 3, opposite strand	-2.57	0.00
ENSMUSG00000085747	Slc13a2os	solute carrier family 13 (sodium-dependent dicarboxylate transporter), member 2, opposite stra	-2.57	0.04
ENSMUSG00000028811	Yars1	tyrosyl-tRNA synthetase 1	-2.56	0.00
ENSMUSG00000120036	Gm57017	predicted gene, 57017	-2.56	0.00
ENSMUSG00000034544	Rsrc1	arginine and serine rich coiled-coil 1	-2.56	0.01
ENSMUSG00000025142	Aspscr1	ASPSCR1 tether for SLC2A4, UBX domain containing	-2.56	0.04
ENSMUSG00000035934	Pknox2	PBX/knotted 1 homeobox 2	-2.55	0.04
ENSMUSG00000014850	Msh3	mutS homolog 3	-2.55	0.00
ENSMUSG00000038930	Rccd1	RCC1 domain containing 1	-2.55	0.01
ENSMUSG00000046318	Ccbe1	collagen and calcium binding EGF domains 1	-2.55	0.00
ENSMUSG00000071074	Yipf3	Yip1 domain family member 3	-2.55	0.00
ENSMUSG00000034059	Ypel4	yippee like 4	-2.54	0.00
ENSMUSG00000027329	Spef1	sperm flagellar 1	-2.54	0.00
ENSMUSG00000097445	Gm26631	predicted gene, 26631	-2.54	0.00
ENSMUSG00000109647	Gm45510	predicted gene 45510	-2.54	0.00
ENSMUSG00000024462	Gabbr1	gamma-aminobutyric acid type B receptor subunit 1	-2.53	0.00
ENSMUSG00000020914	Top2a	DNA topoisomerase II alpha	-2.53	0.03
ENSMUSG00000041040	Fam117b	family with sequence similarity 117 member B	-2.53	0.00
ENSMUSG00000027171	Prrg4	proline rich and Gla domain 4	-2.52	0.01
ENSMUSG00000115122	Gm49685	predicted gene, 49685	-2.52	0.00
ENSMUSG00000110516	Gm45758	predicted gene 45758	-2.52	0.00
ENSMUSG00000022972	Cfap298	cilia and flagella associated protein 298	-2.51	0.01
ENSMUSG00000098041	Gm26981	predicted gene, 26981	-2.51	0.00

Essemble ID	Gene Symbol	Gene Description	Fold Change	P Value
ENSMUSG00000087593	Gm16174	predicted gene 16174	-2.50	0.00
ENSMUSG00000085438	Oip5os1	Opa interacting protein 5, opposite strand 1	-2.50	0.00
ENSMUSG00000117129	Gm49928	predicted gene, 49928	-2.50	0.00
ENSMUSG00000004221	Ikbkg	inhibitor of nuclear factor kappa B kinase regulatory subunit gamma	-2.50	0.00
ENSMUSG00000022091	Sorbs3	sorbin and SH3 domain containing 3	-2.50	0.00
ENSMUSG00000025534	Gusb	glucuronidase beta	-2.50	0.00
ENSMUSG00000033166	Dis3	DIS3 homolog, exosome endoribonuclease and 3'-5' exoribonuclease	-2.49	0.04
ENSMUSG00000022751	Nit2	nitrilase family member 2	-2.49	0.02
ENSMUSG00000047767	Atg16l2	autophagy related 16 like 2	-2.49	0.00
ENSMUSG00000034371	Tkfc	triokinase and FMN cyclase	-2.48	0.00
ENSMUSG00000046591	Ticrr	TOPBP1 interacting checkpoint and replication regulator	-2.48	0.01
ENSMUSG00000005102	Eif2ak4	eukaryotic translation initiation factor 2 alpha kinase 4	-2.48	0.04
ENSMUSG00000091514	Gm17484	predicted gene, 17484	-2.48	0.00
ENSMUSG00000086806	Gm13054	predicted gene 13054	-2.48	0.02
ENSMUSG00000026466	Tor1aip1	torsin 1A interacting protein 1	-2.48	0.00
ENSMUSG00000104330	Gm38285	predicted gene, 38285	-2.47	0.00
ENSMUSG00000024137	E4f1	E4F transcription factor 1	-2.47	0.01
ENSMUSG00000023909	Paqr4	progestin and adipoQ receptor family member 4	-2.47	0.00
ENSMUSG00000021054	Sgpp1	sphingosine-1-phosphate phosphatase 1	-2.47	0.03
ENSMUSG00000060002	Chpt1	choline phosphotransferase 1	-2.46	0.05
ENSMUSG00000079608	Stard6	StAR related lipid transfer domain containing 6	-2.46	0.00
ENSMUSG00000112860	Gm47439	predicted gene, 47439	-2.46	0.00
ENSMUSG00000117004	Gm38593	predicted gene, 38593	-2.46	0.01
ENSMUSG00000086446	Prkag2os1	protein kinase, AMP-activated, gamma 2 non-catalytic subunit, opposite strand 1	-2.46	0.00
ENSMUSG00000050148	Ubqln2	ubiquilin 2	-2.46	0.00
ENSMUSG00000097467	Gm26737	predicted gene, 26737	-2.45	0.00
ENSMUSG00000049327	Kmt5a	lysine methyltransferase 5A	-2.45	0.03
ENSMUSG00000026575	Nme7	NME/NM23 family member 7	-2.45	0.00
ENSMUSG00000031519	Asb5	ankyrin repeat and SOCS box containing 5	-2.44	0.00
ENSMUSG00000109282	Gm45188	predicted gene 45188	-2.44	0.00
ENSMUSG00000038156	Spon1	spondin 1	-2.44	0.00
ENSMUSG00000074220	Zfp382	zinc finger protein 382	-2.43	0.01
ENSMUSG00000087691	Cd55os	CD55 molecule, opposite strand sequence	-2.43	0.00
ENSMUSG00000039470	Zdhhc2	zinc finger DHHC-type palmitoyltransferase 2	-2.43	0.00
ENSMUSG00000107999	Gm44123	predicted gene, 44123	-2.43	0.00
ENSMUSG00000103400	Gm15853	predicted gene 15853	-2.42	0.00
ENSMUSG00000103975	Gm37676	predicted gene, 37676	-2.42	0.00
ENSMUSG00000045826	Ptprcap	protein tyrosine phosphatase receptor type C associated protein	-2.42	0.00
ENSMUSG00000087377	AV099323	expressed sequence AV099323	-2.42	0.00
ENSMUSG00000097412	1810014B01Rik	RIKEN cDNA 1810014B01 gene	-2.41	0.00
ENSMUSG00000097399	Gm26555	predicted gene, 26555	-2.41	0.01
ENSMUSG00000039701	Usp53	ubiquitin specific peptidase 53	-2.41	0.03
ENSMUSG00000087518	Gm13561	predicted gene 13561	-2.41	0.01
ENSMUSG00000029402	Snrnp35	small nuclear ribonucleoprotein U11/U12 subunit 35	-2.41	0.01
ENSMUSG00000024370	Cdc23	cell division cycle 23	-2.41	0.01
ENSMUSG00000097250	Gm26771	predicted gene, 26771	-2.40	0.00

Essemble ID	Gene Symbol	Gene Description	Fold Change	P Value
ENSMUSG00000031823	Zdhhc7	zinc finger DHHC-type palmitoyltransferase 7	-2.39	0.00
ENSMUSG00000021707	Dhfr	dihydrofolate reductase	-2.39	0.00
ENSMUSG00000053358	Gm9905	predicted gene 9905	-2.39	0.00
ENSMUSG00000086401	Gm15559	predicted gene 15559	-2.39	0.00
ENSMUSG00000021140	Pcnx	pecanex 1	-2.39	0.03
ENSMUSG00000114157	Gm47870	predicted gene, 47870	-2.39	0.00
ENSMUSG00000117751	Gm50256	predicted gene, 50256	-2.39	0.01
ENSMUSG00000078652	Psme3	proteasome activator subunit 3	-2.38	0.05
ENSMUSG00000019124	Scrn1	secernin 1	-2.38	0.00
ENSMUSG00000099034	2810039B14Rik	RIKEN cDNA 2810039B14 gene	-2.38	0.00
ENSMUSG00000058392	Rrp1b	ribosomal RNA processing 1B	-2.37	0.02
ENSMUSG00000020869	Lrrc59	leucine rich repeat containing 59	-2.37	0.00
ENSMUSG00000115546	Gm49077	predicted gene, 49077	-2.36	0.00
ENSMUSG00000085500	Gm16976	predicted gene, 16976	-2.36	0.01
ENSMUSG00000044306	4930500M09Rik	RIKEN cDNA 4930500M09 gene	-2.36	0.00
ENSMUSG00000021036	Sptlc2	serine palmitoyltransferase long chain base subunit 2	-2.36	0.04
ENSMUSG00000025980	Hspd1	heat shock protein family D (Hsp60) member 1	-2.36	0.03
ENSMUSG00000005371	Fbxo11	F-box protein 11	-2.35	0.00
ENSMUSG00000070315	4930581F22Rik	RIKEN cDNA 4930581F22 gene	-2.35	0.00
ENSMUSG00000115811	Gm41293	predicted gene, 41293	-2.35	0.01
ENSMUSG00000100320	Gm29417	predicted gene 29417	-2.35	0.00
ENSMUSG00000100931	Gm28085	predicted gene 28085	-2.35	0.00
ENSMUSG00000024666	Tmem138	transmembrane protein 138	-2.35	0.01
ENSMUSG00002075544	Gm56435	predicted gene, 56435	-2.34	0.01
ENSMUSG00000046080	Clec9a	C-type lectin domain containing 9A	-2.34	0.00
ENSMUSG00000101337	Dnah7c	dynein axonemal heavy chain 7	-2.34	0.00
ENSMUSG00000007338	Mrpl49	mitochondrial ribosomal protein L49	-2.34	0.00
ENSMUSG00000025232	Hexa	hexosaminidase subunit alpha	-2.34	0.01
ENSMUSG00000031441	Atp11a	ATPase phospholipid transporting 11A	-2.33	0.03
ENSMUSG00000062470	Fbxl12os	F-box and leucine-rich repeat protein 12, opposite strand	-2.33	0.00
ENSMUSG00000118057	B020010K11Rik	RIKEN cDNA B020010K11 gene	-2.33	0.01
ENSMUSG00000108320	Gm44877	predicted gene 44877	-2.33	0.00
ENSMUSG00000031485	Plpbp	pyridoxal phosphate binding protein	-2.33	0.03
ENSMUSG00000055401	Fbxo6	F-box protein 6	-2.33	0.00
ENSMUSG00000112297	Gm17816	ubiquitin associated protein 2-like pseudogene	-2.32	0.00
ENSMUSG00000070730	Rmdn3	regulator of microtubule dynamics 3	-2.32	0.00
ENSMUSG00000033434	Gtpbp6	GTP binding protein 6 (putative)	-2.32	0.01
ENSMUSG00000013928	1700020L13Rik	RIKEN cDNA 1700020L13 gene	-2.32	0.00
ENSMUSG00000022471	Xrcc6	X-ray repair cross complementing 6	-2.31	0.00
ENSMUSG00000006095	Tbcb	tubulin folding cofactor B	-2.31	0.00
ENSMUSG00000097610	A930012L18Rik	RIKEN cDNA A930012L18 gene	-2.31	0.00
ENSMUSG00000079355	Ackr4	atypical chemokine receptor 4	-2.30	0.00
ENSMUSG00000030979	Uros	uroporphyrinogen III synthase	-2.30	0.05
ENSMUSG00000115801	Gm55330	predicted gene, 55330	-2.30	0.01
ENSMUSG00000002885	Adgre5	adhesion G protein-coupled receptor E5	-2.30	0.00
ENSMUSG00000111864	Rpl26-ps6	ribosomal protein L26, pseudogene 6	-2.29	0.00

Essemble ID	Gene Symbol	Gene Description	Fold Change	P Value
ENSMUSG00000017491	Rarb	retinoic acid receptor beta	-2.29	0.00
ENSMUSG00000084869	Gm13063	predicted gene 13063	-2.29	0.00
ENSMUSG00000091133	Gm17108	predicted gene 17108	-2.29	0.00
ENSMUSG00000106108	Gm43221	predicted gene 43221	-2.29	0.00
ENSMUSG00000026546	Cfap45	cilia and flagella associated protein 45	-2.28	0.00
ENSMUSG00000026558	Uck2	uridine-cytidine kinase 2	-2.28	0.03
ENSMUSG00000006527	Sfmbt1	Scm like with four mbt domains 1	-2.28	0.02
ENSMUSG00000097101	1810034E14Rik	RIKEN cDNA 1810034E14 gene	-2.28	0.00
ENSMUSG00000020441	2310033P09Rik	chromosome 1 open reading frame 35	-2.27	0.01
ENSMUSG00000106978	N4bp2os	NEDD4 binding protein 2, opposite strand	-2.27	0.00
ENSMUSG00000107245	Gm42803	predicted gene 42803	-2.27	0.00
ENSMUSG00000086421	Gm14091	predicted gene 14091	-2.27	0.01
ENSMUSG00000097745	AI115009	expressed sequence AI115009	-2.27	0.00
ENSMUSG00000107954	Gm43942	predicted gene, 43942	-2.27	0.00
ENSMUSG00000090441	Gm17651	predicted gene, 17651	-2.27	0.00
ENSMUSG00000058835	Abi1	abl interactor 1	-2.27	0.00
ENSMUSG00000115025	Gm4240	ribosomal protein L17 pseudogene	-2.26	0.00
ENSMUSG00000097048	1600020E01Rik	RIKEN cDNA 1600020E01 gene	-2.26	0.00
ENSMUSG00000109130	Gm45187	predicted gene 45187	-2.26	0.00
ENSMUSG00000026950	Neb	nebulin	-2.26	0.03
ENSMUSG00000066475	Gm10268	60S ribosomal protein L17 pseudogene	-2.25	0.00
ENSMUSG00000019320	Noxo1	NADPH oxidase organizer 1	-2.25	0.00
ENSMUSG00000028557	Rnf11	ring finger protein 11	-2.25	0.00
ENSMUSG00000036036	Zfp57	ZFP57 zinc finger protein	-2.25	0.01
ENSMUSG00000108425	Gm44706	predicted gene 44706	-2.25	0.00
ENSMUSG00000055850	Rnf181	ring finger protein 181	-2.25	0.04
ENSMUSG00000022369	Mtbp	MDM2 binding protein	-2.25	0.00
ENSMUSG00000021288	Klc1	kinesin light chain 1	-2.25	0.00
ENSMUSG00000118210	Gm50394	predicted gene, 50394	-2.24	0.04
ENSMUSG00000061904	Slc25a3	solute carrier family 25 member 3	-2.24	0.04
ENSMUSG00000092539	Gm20468	predicted gene 20468	-2.24	0.00
ENSMUSG00000106662	Gm43034	predicted gene 43034	-2.24	0.00
ENSMUSG00000117477	Gm50092	predicted gene, 50092	-2.24	0.00
ENSMUSG00000052926	Rnaseh2a	ribonuclease H2 subunit A	-2.24	0.00
ENSMUSG00000048578	Mlec	malectin	-2.23	0.00
ENSMUSG00000053310	Nrgn	neurogranin	-2.23	0.00
ENSMUSG00000087206	Gm16175	predicted gene 16175	-2.23	0.00
ENSMUSG00000113087	4930473H19Rik	RIKEN cDNA 4930473H19 gene	-2.23	0.00
ENSMUSG00000056682	4930465K10Rik	RIKEN cDNA 4930465K10 gene	-2.22	0.01
ENSMUSG00000085715	Tsix	TSIX transcript, XIST antisense RNA	-2.22	0.00
ENSMUSG00000026797	Stxbp1	syntaxin binding protein 1	-2.22	0.00
ENSMUSG00000022146	Osmr	oncostatin M receptor	-2.22	0.01
ENSMUSG00000056116	H2-T22	histocompatibility 2, T region locus 22	-2.22	0.00
ENSMUSG00000110611	Gm20163	predicted gene, 20163	-2.21	0.00
ENSMUSG00000038987	Cfap157	cilia and flagella associated protein 157	-2.21	0.00
ENSMUSG00000036168	Ccdc38	coiled-coil domain containing 38	-2.21	0.00

Essemble ID	Gene Symbol	Gene Description	Fold Change	P Value
ENSMUSG00000053825	Ppfia2	PTPRF interacting protein alpha 2	-2.20	0.00
ENSMUSG00000027175	Tcp11l1	t-complex 11 like 1	-2.20	0.01
ENSMUSG00000110221	Gm36210	predicted gene, 36210	-2.20	0.01
ENSMUSG00000005233	Spc25	SPC25 component of NDC80 kinetochore complex	-2.20	0.03
ENSMUSG00000004980	Hnrnpa2b1	heterogeneous nuclear ribonucleoprotein A2/B1	-2.19	0.04
ENSMUSG00000104600	Dmrt1i	Dmrt1 interacting ncRNA	-2.19	0.00
ENSMUSG00000085509	Gm11521	predicted gene 11521	-2.19	0.00
ENSMUSG00000120289	6820445E23Rik	RIKEN cDNA 6820445E23 gene	-2.19	0.00
ENSMUSG00000002580	Mien1	migration and invasion enhancer 1	-2.19	0.00
ENSMUSG00000098062	Gm26931	predicted gene, 26931	-2.19	0.00
ENSMUSG00000075224	Lrrc55	leucine rich repeat containing 55	-2.18	0.01
ENSMUSG00000021340	Gpld1	glycosylphosphatidylinositol specific phospholipase D1	-2.18	0.00
ENSMUSG00000087552	Gm15819	predicted gene 15819	-2.18	0.00
ENSMUSG00002075094	Gm55587	predicted gene, 55587	-2.18	0.00
ENSMUSG00000040648	Ppip5k2	diphosphoinositol pentakisphosphate kinase 2	-2.18	0.00
ENSMUSG00000054717	Hmgb2	high mobility group box 2	-2.18	0.01
ENSMUSG00000045763	Basp1	brain abundant membrane attached signal protein 1	-2.17	0.01
ENSMUSG00000112302	Gm48226	predicted gene, 48226	-2.17	0.03
ENSMUSG00000033486	Catsper2	cation channel sperm associated 2	-2.17	0.03
ENSMUSG00000022070	Bora	BORA aurora kinase A activator	-2.16	0.00
ENSMUSG00000032041	Tirap	TIR domain containing adaptor protein	-2.16	0.02
ENSMUSG00000103710	D030062O11Rik	RIKEN cDNA D030062O11 gene	-2.16	0.05
ENSMUSG00000047907	Tshz2	teashirt zinc finger homeobox 2	-2.15	0.00
ENSMUSG00000086407	Gm14123	predicted gene 14123	-2.15	0.00
ENSMUSG00000020740	Gga3	golgi associated, gamma adaptin ear containing, ARF binding protein 3	-2.15	0.00
ENSMUSG00000024660	Incenp	inner centromere protein	-2.15	0.01
ENSMUSG00000031696	Vps35	VPS35 retromer complex component	-2.15	0.01
ENSMUSG00000025880	Smad7	SMAD family member 7	-2.15	0.04
ENSMUSG00000028549	Itgb3bp	integrin subunit beta 3 binding protein	-2.15	0.00
ENSMUSG00000035781	R3hdm4	R3H domain containing 4	-2.15	0.00
ENSMUSG00000084799	Ino80dos	INO80 complex subunit D, opposite strand	-2.15	0.00
ENSMUSG00000050821	Fam131a	family with sequence similarity 131 member A	-2.14	0.01
ENSMUSG00000046145	1700018M17Rik	RIKEN cDNA 1700018M17 gene	-2.14	0.00
ENSMUSG00000085731	Gm16229	predicted gene 16229	-2.14	0.00
ENSMUSG00000083907	Plk-ps1	polo like kinase, pseudogene 1	-2.13	0.00
ENSMUSG00000028329	Xpa	XPA, DNA damage recognition and repair factor	-2.13	0.00
ENSMUSG00000038416	Cdc16	cell division cycle 16	-2.13	0.01
ENSMUSG00000086740	Gm17029	predicted gene 17029	-2.13	0.00
ENSMUSG00000116716	Gm49643	predicted gene, 49643	-2.13	0.00
ENSMUSG00000121028	Gm56867	predicted gene, 56867	-2.13	0.00
ENSMUSG00000040775	Gm9772	zinc finger protein 758 pseudogene	-2.12	0.01
ENSMUSG00000025505	Tmem80	transmembrane protein 80	-2.12	0.00
ENSMUSG00000044350	Lacc1	laccase domain containing 1	-2.12	0.00
ENSMUSG00000002831	Plin4	perilipin 4	-2.11	0.01
ENSMUSG00000031788	Kifc3	kinesin family member C3	-2.11	0.00
ENSMUSG00000091154	Proscos	proline synthetase co-transcribed, opposite strand	-2.11	0.00

Essemble ID	Gene Symbol	Gene Description	Fold Change	P Value
ENSMUSG00000113068	Gm48133	predicted gene, 48133	-2.11	0.00
ENSMUSG00002076468	Gm55032	predicted gene, 55032	-2.11	0.01
ENSMUSG00000057359	Gm17494	predicted gene, 17494	-2.11	0.01
ENSMUSG00000056411	Gm12500	predicted gene 12500	-2.11	0.00
ENSMUSG00000021714	Cenpk	centromere protein K	-2.11	0.00
ENSMUSG00000084892	Gm14471	predicted gene 14471	-2.11	0.00
ENSMUSG00000061887	Ssbp3	single stranded DNA binding protein 3	-2.11	0.02
ENSMUSG00000109036	2210406H18Rik	RIKEN cDNA 2210406H18 gene	-2.11	0.00
ENSMUSG00000097491	Gm2366	predicted gene 2366	-2.11	0.00
ENSMUSG00000087443	Ppp1r18os	protein phosphatase 1, regulatory subunit 18, opposite strand	-2.10	0.00
ENSMUSG00000022064	Pibf1	progesterone immunomodulatory binding factor 1	-2.10	0.00
ENSMUSG00000090115	Usp49	ubiquitin specific peptidase 49	-2.10	0.02
ENSMUSG00002076403	Gm56278	predicted gene, 56278	-2.10	0.00
ENSMUSG00000046675	Lyset	lysosomal enzyme trafficking factor	-2.10	0.00
ENSMUSG00000101308	Gm28989	predicted gene 28989	-2.10	0.00
ENSMUSG00000118523	Gm34319	predicted gene, 34319	-2.10	0.00
ENSMUSG00000108624	Gm45091	predicted gene 45091	-2.09	0.04
ENSMUSG00000102559	Gm37570	predicted gene, 37570	-2.09	0.04
ENSMUSG00000114470	Gm49395	predicted gene, 49395	-2.09	0.02
ENSMUSG00000036078	Sigmar1	sigma non-opioid intracellular receptor 1	-2.09	0.00
ENSMUSG00000021018	Polr2h	RNA polymerase II, I and III subunit H	-2.09	0.04
ENSMUSG00000086418	Gm13362	predicted gene 13362	-2.09	0.00
ENSMUSG00000097240	Gm26614	predicted gene, 26614	-2.08	0.04
ENSMUSG00000111632	Gm47327	predicted gene, 47327	-2.08	0.00
ENSMUSG00000046245	Pilra	paired immunoglobin like type 2 receptor alpha	-2.08	0.00
ENSMUSG00000032112	Trappc4	trafficking protein particle complex subunit 4	-2.08	0.00
ENSMUSG00000026018	Ica1l	islet cell autoantigen 1 like	-2.08	0.00
ENSMUSG00000014747	Ankrd53	ankyrin repeat domain 53	-2.08	0.01
ENSMUSG00000085664	Atxn7l1os2	ataxin 7-like 1, opposite strand 2	-2.08	0.00
ENSMUSG00000105198	Gm42502	predicted gene 42502	-2.08	0.01
ENSMUSG00000002010	Idh3g	isocitrate dehydrogenase (NAD(+)) 3 non-catalytic subunit gamma	-2.07	0.04
ENSMUSG00000108391	Gm9768	predicted gene 9768	-2.07	0.03
ENSMUSG00000028483	Snapc3	small nuclear RNA activating complex polypeptide 3	-2.07	0.00
ENSMUSG00000113852	Gm48062	predicted gene, 48062	-2.07	0.01
ENSMUSG00000009621	Vav2	vav guanine nucleotide exchange factor 2	-2.07	0.02
ENSMUSG00000032307	Ube2q2	ubiquitin conjugating enzyme E2 Q2	-2.06	0.00
ENSMUSG00000013622	Atraid	all-trans retinoic acid induced differentiation factor	-2.06	0.01
ENSMUSG00000064090	Vrk2	VRK serine/threonine kinase 2	-2.06	0.00
ENSMUSG00000032036	Kirrel3	kirre like nephrin family adhesion molecule 3	-2.06	0.01
ENSMUSG00000052241	A930035D04Rik	RIKEN cDNA A930035D04 gene	-2.06	0.00
ENSMUSG00000071652	Ints5	integrator complex subunit 5	-2.06	0.02
ENSMUSG00000085355	3010003L21Rik	RIKEN cDNA 3010003L21 gene	-2.05	0.00
ENSMUSG00000107502	Gm44065	predicted gene, 44065	-2.05	0.00
ENSMUSG00000021420	Fars2	phenylalanyl-tRNA synthetase 2, mitochondrial	-2.05	0.00
ENSMUSG00000087022	9130024F11Rik	RIKEN cDNA 9130024F11 gene	-2.05	0.02
ENSMUSG00000006010	Odr4	odr-4 GPCR localization factor homolog	-2.05	0.01

Essemble ID	Gene Symbol	Gene Description	Fold Change	P Value
ENSMUSG00000035953	Pip4p1	phosphatidylinositol-4,5-bisphosphate 4-phosphatase 1	-2.05	0.00
ENSMUSG00000019761	Krt10	keratin 10	-2.05	0.03
ENSMUSG00000087132	A930001C03Rik	RIKEN cDNA A930001C03 gene	-2.04	0.00
ENSMUSG00000039133	9330171B17Rik	RIKEN cDNA 9330171B17 gene	-2.04	0.00
ENSMUSG00000067367	Lyar	Ly1 antibody reactive	-2.04	0.01
ENSMUSG00000045968	Teddm2	transmembrane epididymal family member 2	-2.04	0.00
ENSMUSG00000117710	Gm10817	predicted gene 10817	-2.04	0.00
ENSMUSG00000026228	Htr2b	5-hydroxytryptamine receptor 2B	-2.04	0.01
ENSMUSG00000091155	Serpine3	serpin family E member 3	-2.03	0.00
ENSMUSG00000121133	Gm56865	predicted gene, 56865	-2.03	0.00
ENSMUSG00000100084	2310068J16Rik	RIKEN cDNA 2310068J16 gene	-2.03	0.00
ENSMUSG00000087433	Gm14167	predicted gene 14167	-2.03	0.02
ENSMUSG00000083898	Gm7340	predicted gene 7340	-2.03	0.00
ENSMUSG00000040681	Hmgn1	high mobility group nucleosome binding domain 1	-2.03	0.00
ENSMUSG00000079286	Gm11084	predicted gene 11084	-2.03	0.05
ENSMUSG00000116223	Gm41396	predicted gene, 41396	-2.03	0.00
ENSMUSG00000091507	Gm17194	predicted gene 17194	-2.03	0.01
ENSMUSG00000107086	Gm43808	predicted gene 43808	-2.02	0.00
ENSMUSG00000087397	Rapgef3os2	Rap guanine nucleotide exchange factor (GEF) 3, opposite strand 2	-2.02	0.04
ENSMUSG00000087190	D430001F17Rik	RIKEN cDNA D430001F17 gene	-2.02	0.00
ENSMUSG00000024131	Slc3a1	solute carrier family 3 member 1	-2.02	0.00
ENSMUSG00000018581	Dnah11	dynein axonemal heavy chain 11	-2.02	0.00
ENSMUSG00000019763	Rmnd1	required for meiotic nuclear division 1 homolog	-2.02	0.01
ENSMUSG00000082860	Gm13204	predicted gene 13204	-2.02	0.00
ENSMUSG00000031902	Nfatc3	nuclear factor of activated T cells 3	-2.02	0.00
ENSMUSG00000045962	Wnk1	WNK lysine deficient protein kinase 1	-2.02	0.01
ENSMUSG00000022476	Polr3h	RNA polymerase III subunit H	-2.01	0.00
ENSMUSG00000097407	4933408J17Rik	RIKEN cDNA 4933408J17 gene	-2.01	0.00
ENSMUSG00000003038	Hmgn2	high mobility group nucleosomal binding domain 2	-2.01	0.01
ENSMUSG00000029363	Rfc5	replication factor C subunit 5	-2.01	0.00
ENSMUSG00000044807	Zfp354c	zinc finger protein 354C	-2.01	0.05
ENSMUSG00000030159	Clec1b	C-type lectin domain family 1 member B	-2.01	0.02
ENSMUSG00000025816	Sec61a2	SEC61 translocon subunit alpha 2	-2.01	0.02
ENSMUSG00000108720	Gm44672	predicted gene 44672	-2.01	0.00
ENSMUSG00000086839	Gm11973	predicted gene 11973	-2.01	0.01
ENSMUSG00000109485	Gm45016	predicted gene 45016	-2.01	0.04
ENSMUSG00000043991	Pura	purine rich element binding protein A	-2.00	0.02
ENSMUSG00000027827	Kcnab1	potassium voltage-gated channel subfamily A regulatory beta subunit 1	-2.00	0.04
ENSMUSG00000049517	Rps23	ribosomal protein S23	2.00	0.02
ENSMUSG00000097877	Gm26703	predicted gene, 26703	2.00	0.00
ENSMUSG00000006517	Mvd	mevalonate diphosphate decarboxylase	2.00	0.00
ENSMUSG00000026229	Psmd1	proteasome 26S subunit, non-ATPase 1	2.01	0.00
ENSMUSG00000000532	Acvr1b	activin A receptor type 1B	2.01	0.00
ENSMUSG00000042790	Rnf214	ring finger protein 214	2.01	0.00
ENSMUSG00000114585	Gm32401	predicted gene, 32401	2.01	0.02
ENSMUSG00000020610	Amz2	archaelysin family metallopeptidase 2	2.01	0.00

Essemble ID	Gene Symbol	Gene Description	Fold Change	P Value
ENSMUSG00000032998	Foxj3	forkhead box J3	2.01	0.00
ENSMUSG00000018476	Kdm6b	lysine demethylase 6B	2.01	0.00
ENSMUSG00000111268	Gm47126	predicted gene, 47126	2.01	0.01
ENSMUSG00000019471	Cdc37	cell division cycle 37, HSP90 cochaperone	2.02	0.01
ENSMUSG00000090062	Galnt6os	polypeptide N-acetylgalactosaminyltransferase 6, opposite strand	2.02	0.00
ENSMUSG00000002393	Nr2f6	nuclear receptor subfamily 2 group F member 6	2.02	0.03
ENSMUSG00000029859	Epha1	EPH receptor A1	2.03	0.00
ENSMUSG00000102564	Gm37035	predicted gene, 37035	2.03	0.00
ENSMUSG00000030271	Ogg1	8-oxoguanine DNA glycosylase	2.03	0.00
ENSMUSG00000097887	Gm26542	predicted gene, 26542	2.03	0.00
ENSMUSG00002075963	Gm56108	predicted gene, 56108	2.03	0.00
ENSMUSG00000026726	Cubn	cubilin	2.03	0.02
ENSMUSG00000107090	Gm42884	predicted gene 42884	2.03	0.00
ENSMUSG00000108257	Gm44251	predicted gene, 44251	2.03	0.04
ENSMUSG00000097768	2310043M15Rik	RIKEN cDNA 2310043M15 gene	2.03	0.00
ENSMUSG00000051495	Irf2bp2	interferon regulatory factor 2 binding protein 2	2.04	0.02
ENSMUSG00000039953	Clstn1	calsyntenin 1	2.04	0.01
ENSMUSG00000029530	Ccr9	C-C motif chemokine receptor 9	2.04	0.00
ENSMUSG00000018707	Dync1h1	dynein cytoplasmic 1 heavy chain 1	2.04	0.03
ENSMUSG00000037111	Setd7	SET domain containing 7, histone lysine methyltransferase	2.04	0.00
ENSMUSG00000028597	Gpx7	glutathione peroxidase 7	2.04	0.04
ENSMUSG00000091191	Gm17334	predicted gene, 17334	2.04	0.00
ENSMUSG00000086058	Unc45bos	unc-45 myosin chaperone B, opposite strand	2.04	0.00
ENSMUSG00000086648	Gm15511	predicted gene 15511	2.05	0.00
ENSMUSG00000055435	Maf	MAF bZIP transcription factor	2.05	0.02
ENSMUSG00000034111	Tmed8	transmembrane p24 trafficking protein family member 8	2.05	0.01
ENSMUSG00000097974	Gm10605	predicted gene 10605	2.05	0.00
ENSMUSG00000045128	Rpl18a	ribosomal protein L18a	2.05	0.03
ENSMUSG00000017679	Ttpal	alpha tocopherol transfer protein like	2.05	0.00
ENSMUSG00000117337	Gm49964	predicted gene, 49964	2.05	0.01
ENSMUSG00000084786	Ubl5	ubiquitin like 5	2.05	0.00
ENSMUSG00000121068	Gm57050	predicted gene, 57050	2.05	0.01
ENSMUSG00000051844	B230319C09Rik	RIKEN cDNA B230319C09 gene	2.05	0.01
ENSMUSG00000032085	Tagln	transgelin	2.06	0.00
ENSMUSG00000022312	Eif3h	eukaryotic translation initiation factor 3 subunit H	2.06	0.01
ENSMUSG00000040842	Szrd1	SUZ RNA binding domain containing 1	2.06	0.05
ENSMUSG00000121083	4930423K06Rik	RIKEN cDNA 4930423K06 gene	2.06	0.01
ENSMUSG00000093629	Prox2os	prospero homeobox 2 opposite strand	2.07	0.00
ENSMUSG00000002428	Hltf	helicase like transcription factor	2.07	0.01
ENSMUSG00000072612	Gm10382	predicted gene 10382	2.07	0.00
ENSMUSG00000047986	Palm3	paralemmin 3	2.07	0.04
ENSMUSG00000104605	Gm42922	predicted gene 42922	2.07	0.03
ENSMUSG00000087644	Gm14703	predicted gene 14703	2.07	0.00
ENSMUSG00000024969	Mark2	microtubule affinity regulating kinase 2	2.07	0.02
ENSMUSG00000114702	Gm40828	predicted gene, 40828	2.08	0.00
ENSMUSG00000036002	Atosb	atos homolog B	2.08	0.00

Essemble ID	Gene Symbol	Gene Description	Fold Change	P Value
ENSMUSG00000038387	Rras	RAS related	2.08	0.00
ENSMUSG00000002718	Cse1l	chromosome segregation 1 like	2.08	0.00
ENSMUSG00000081512	Gm15821	predicted gene 15821	2.08	0.00
ENSMUSG00000033209	Ttc28	tetratricopeptide repeat domain 28	2.08	0.01
ENSMUSG00000044709	Gemin7	gem nuclear organelle associated protein 7	2.08	0.01
ENSMUSG00000034247	Plekhm1	pleckstrin homology and RUN domain containing M1	2.08	0.00
ENSMUSG00000108832	Gm44832	predicted gene 44832	2.08	0.01
ENSMUSG00000036241	Ube2r2	ubiquitin conjugating enzyme E2 R2	2.08	0.01
ENSMUSG00000097111	Peak1os	pseudopodium-enriched atypical kinase 1, opposite strand	2.09	0.00
ENSMUSG00000084925	1810062O18Rik	RIKEN cDNA 1810062O18 gene	2.09	0.00
ENSMUSG00000105799	Mir126b	microRNA 126	2.09	0.05
ENSMUSG00000045302	Preb	prolactin regulatory element binding	2.09	0.00
ENSMUSG00000040723	Rcsd1	RCSD domain containing 1	2.09	0.02
ENSMUSG00000029030	Tprg1l	tumor protein p63 regulated 1 like	2.10	0.01
ENSMUSG00000020889	Nr1d1	nuclear receptor subfamily 1 group D member 1	2.10	0.00
ENSMUSG00000116884	C130040N14Rik	RIKEN cDNA C130040N14 gene	2.10	0.00
ENSMUSG00000029810	Tmem176b	transmembrane protein 176B	2.10	0.01
ENSMUSG00000031021	Tmem9b	TMEM9 domain family member B	2.10	0.00
ENSMUSG00000038587	Akap12	A-kinase anchoring protein 12	2.10	0.02
ENSMUSG00000054364	Rhob	ras homolog family member B	2.10	0.01
ENSMUSG00000120324	Gm57007	predicted gene, 57007	2.10	0.00
ENSMUSG00000061689	Dlgap4	DLG associated protein 4	2.10	0.01
ENSMUSG00000021391	Cenpp	centromere protein P	2.10	0.00
ENSMUSG00000029250	Polr2b	RNA polymerase II subunit B	2.10	0.00
ENSMUSG00000052403	Fcnaos	ficolin A, opposite strand	2.11	0.04
ENSMUSG00000086930	Frs3os	fibroblast growth factor receptor substrate 3, opposite strand	2.11	0.00
ENSMUSG00000000157	Itgb2l	integrin beta 2-like	2.11	0.01
ENSMUSG00000090019	Gimap1	GTPase, IMAP family member 1	2.11	0.01
ENSMUSG00000030744	Rps3	ribosomal protein S3	2.11	0.03
ENSMUSG00000070604	Vsig10l	V-set and immunoglobulin domain containing 10 like	2.11	0.00
ENSMUSG00000027615	Hps3	HPS3 biogenesis of lysosomal organelles complex 2 subunit 1	2.11	0.01
ENSMUSG00000106433	Gm32102	predicted gene, 32102	2.11	0.02
ENSMUSG00000078772	Gm12353	predicted gene 12353	2.12	0.00
ENSMUSG00000031592	Pcm1	pericentriolar material 1	2.12	0.04
ENSMUSG00000058812	0610039K10Rik	RIKEN cDNA 0610039K10 gene	2.12	0.00
ENSMUSG00000070462	Tlnrd1	talin rod domain containing 1	2.12	0.00
ENSMUSG00000023949	Tcte1	t-complex-associated-testis-expressed 1	2.12	0.02
ENSMUSG00000040034	Nup43	nucleoporin 43	2.12	0.00
ENSMUSG00000029552	Tes	testin LIM domain protein	2.12	0.01
ENSMUSG00000109408	A930037H05Rik	RIKEN cDNA A930037H05 gene	2.12	0.01
ENSMUSG00000018474	Chd3	chromodomain helicase DNA binding protein 3	2.12	0.01
ENSMUSG00000120623	Gm56503	predicted gene, 56503	2.13	0.02
ENSMUSG00000021182	Ccdc88c	coiled-coil domain containing 88C	2.13	0.04
ENSMUSG00000074398	Gm15441	predicted gene 15441	2.13	0.00
ENSMUSG00000029108	Pcdh7	protocadherin 7	2.13	0.00
ENSMUSG00000044950	Pwwp2a	PWWP domain containing 2A	2.13	0.01

Essemble ID	Gene Symbol	Gene Description	Fold Change	P Value
ENSMUSG00000108353	Gm45205	predicted gene 45205	2.13	0.00
ENSMUSG00000000346	Dazap2	DAZ associated protein 2	2.13	0.00
ENSMUSG00000106736	Gm42573	predicted gene 42573	2.13	0.00
ENSMUSG00000069678	Pcgf1	polycomb group ring finger 1	2.13	0.03
ENSMUSG00000032030	Cul5	cullin 5	2.14	0.00
ENSMUSG00000048796	Cyb561d1	cytochrome b561 family member D1	2.14	0.02
ENSMUSG00000028059	Arhgef2	Rho/Rac guanine nucleotide exchange factor 2	2.14	0.03
ENSMUSG00000027394	Ttl	tubulin tyrosine ligase	2.14	0.00
ENSMUSG00000060032	H2aj	H2A.J histone	2.15	0.04
ENSMUSG00000049502	Dtx3l	deltex E3 ubiquitin ligase 3L	2.15	0.00
ENSMUSG00000016528	Mapkapk2	MAPK activated protein kinase 2	2.15	0.03
ENSMUSG00000039798	2600006K01Rik	RIKEN cDNA 2600006K01 gene	2.15	0.00
ENSMUSG00000016253	Nelfcd	negative elongation factor complex member C/D	2.15	0.00
ENSMUSG00000038623	Tm6sf1	transmembrane 6 superfamily member 1	2.15	0.00
ENSMUSG00000096727	Psmb9	proteasome 20S subunit beta 9	2.15	0.00
ENSMUSG00000104234	5330439B14Rik	RIKEN cDNA 5330439B14 gene	2.16	0.00
ENSMUSG00000031749	St3gal2	ST3 beta-galactoside alpha-2,3-sialyltransferase 2	2.16	0.00
ENSMUSG00000110980	Gm47204	predicted gene, 47204	2.16	0.00
ENSMUSG00000031320	Rps4x	ribosomal protein S4 Y-linked 1	2.16	0.04
ENSMUSG00000028780	Sema3c	semaphorin 3C	2.16	0.03
ENSMUSG00000086560	Gm13372	predicted gene 13372	2.16	0.01
ENSMUSG00000036819	Jmjd4	jumonji domain containing 4	2.17	0.00
ENSMUSG00000090034	Gm15917	predicted gene 15917	2.17	0.00
ENSMUSG00000000740	Rpl13	ribosomal protein L13	2.17	0.00
ENSMUSG00000089701	Gm15978	predicted gene 15978	2.17	0.00
ENSMUSG00000024608	Rps14	ribosomal protein S14	2.17	0.01
ENSMUSG00000064350	mt-Ty	mitochondrially encoded tRNA tyrosine	2.17	0.00
ENSMUSG00000086593	Gm16548	predicted gene 16548	2.17	0.00
ENSMUSG00000042254	Cilp	cartilage intermediate layer protein	2.17	0.03
ENSMUSG00000087639	Gm15512	predicted gene 15512	2.17	0.00
ENSMUSG00000120252	Gm31504	predicted gene, 31504	2.17	0.01
ENSMUSG00000029516	Cit	citron rho-interacting serine/threonine kinase	2.18	0.04
ENSMUSG00000087411	Gm16048	predicted gene 16048	2.18	0.00
ENSMUSG00000047067	Dusp28	dual specificity phosphatase 28	2.18	0.00
ENSMUSG00000073144	4930599N23Rik	RIKEN cDNA 4930599N23 gene	2.18	0.00
ENSMUSG00000021624	Cd180	CD180 molecule	2.18	0.00
ENSMUSG00000038600	Atp6v0a4	ATPase H+ transporting V0 subunit a4	2.18	0.04
ENSMUSG00000038213	Tapbpl	TAP binding protein like	2.18	0.04
ENSMUSG00000012848	Rps5	ribosomal protein S5	2.19	0.01
ENSMUSG00000104379	Gm37509	predicted gene, 37509	2.19	0.01
ENSMUSG00000085653	Gm15179	predicted gene 15179	2.19	0.01
ENSMUSG00000001911	Nfix	nuclear factor I X	2.19	0.00
ENSMUSG00000025508	Rplp2	ribosomal protein lateral stalk subunit P2	2.19	0.03
ENSMUSG00000090733	Rps27	ribosomal protein S27	2.19	0.00
ENSMUSG00000028245	Nsmaf	neutral sphingomyelinase activation associated factor	2.19	0.00
ENSMUSG00000035275	Raver2	ribonucleoprotein, PTB binding 2	2.19	0.00

Essemble ID	Gene Symbol	Gene Description	Fold Change	P Value
ENSMUSG00000087135	Gm16096	predicted gene 16096	2.19	0.00
ENSMUSG00000030329	Pianp	PILR alpha associated neural protein	2.19	0.01
ENSMUSG00000107846	Gm43963	predicted gene, 43963	2.20	0.00
ENSMUSG00000048292	Or10v5	olfactory receptor family 10 subfamily V member 5	2.20	0.02
ENSMUSG00000097558	Gm26902	predicted gene, 26902	2.20	0.02
ENSMUSG00000032046	Abhd12	abhydrolase domain containing 12, lysophospholipase	2.20	0.00
ENSMUSG00000121149	Gm57157	predicted gene, 57157	2.20	0.00
ENSMUSG00000087052	Gm8093	predicted gene 8093	2.20	0.00
ENSMUSG00000028234	Rps20	ribosomal protein S20	2.21	0.01
ENSMUSG00000079641	Rpl39	ribosomal protein L39	2.21	0.04
ENSMUSG00000043017	Ptgir	prostaglandin I2 receptor	2.21	0.02
ENSMUSG00000011877	Git1	GIT ArfGAP 1	2.21	0.01
ENSMUSG00000005705	Agrp	agouti related neuropeptide	2.22	0.00
ENSMUSG00000015474	Ppt2	palmitoyl-protein thioesterase 2	2.22	0.00
ENSMUSG00000056476	Med12l	mediator complex subunit 12L	2.22	0.00
ENSMUSG00000063882	Uqcrh	ubiquinol-cytochrome c reductase hinge protein like	2.22	0.01
ENSMUSG00000061315	Naca	nascent polypeptide associated complex subunit alpha	2.23	0.01
ENSMUSG00000020704	Asic2	acid sensing ion channel subunit 2	2.23	0.00
ENSMUSG00000109715	Gm45606	predicted gene 45606	2.23	0.00
ENSMUSG00000021363	Mak	male germ cell associated kinase	2.23	0.00
ENSMUSG00000112421	4933406B15Rik	RIKEN cDNA 4933406B15 gene	2.23	0.00
ENSMUSG00000064254	Ethe1	ETHE1 persulfide dioxygenase	2.23	0.00
ENSMUSG00000004707	Ly9	lymphocyte antigen 9	2.24	0.02
ENSMUSG00000093577	Gm20632	predicted gene 20632	2.24	0.00
ENSMUSG00000038503	Mesd	mesoderm development LRP chaperone	2.24	0.03
ENSMUSG00000046811	Gltpd2	glycolipid transfer protein domain containing 2	2.24	0.00
ENSMUSG00000120629	Gm56575	predicted gene, 56575	2.24	0.00
ENSMUSG00000110070	Gm45619	predicted gene 45619	2.24	0.00
ENSMUSG00000084876	Gm14965	predicted gene 14965	2.25	0.00
ENSMUSG00000046688	Tifa	TRAF interacting protein with forkhead associated domain	2.25	0.00
ENSMUSG00000051606	2010001K21Rik	RIKEN cDNA 2010001K21 gene	2.25	0.01
ENSMUSG00000039001	Rps21	ribosomal protein S21	2.25	0.02
ENSMUSG00000085232	Gm11962	polymerase (RNA) II (DNA directed) polypeptide D pseudogene	2.25	0.03
ENSMUSG00000027284	Cdan1	codanin 1	2.25	0.00
ENSMUSG00000024900	Cpt1a	carnitine palmitoyltransferase 1A	2.25	0.00
ENSMUSG00002075297	Gm55904	predicted gene, 55904	2.25	0.01
ENSMUSG00000029860	Zyx	zyxin	2.25	0.00
ENSMUSG00000022724	Riox2	ribosomal oxygenase 2	2.25	0.00
ENSMUSG00000020453	Patz1	POZ/BTB and AT hook containing zinc finger 1	2.26	0.00
ENSMUSG00000110698	Gm45875	predicted gene 45875	2.26	0.00
ENSMUSG00000028975	Pex14	peroxisomal biogenesis factor 14	2.26	0.00
ENSMUSG00000028081	Rps3a1	ribosomal protein S3A1	2.26	0.01
ENSMUSG00000087266	Gm15991	predicted gene 15991	2.26	0.01
ENSMUSG00000101210	Gm28720	predicted gene 28720	2.26	0.01
ENSMUSG00000022971	Ifnar2	interferon alpha and beta receptor subunit 2	2.27	0.02
ENSMUSG00000053929	Zftraf1	zinc finger TRAF-type containing 1	2.27	0.00

Essemble ID	Gene Symbol	Gene Description	Fold Change	P Value
ENSMUSG00000078747	Gm20878	predicted gene, 20878	2.27	0.01
ENSMUSG00000097204	Gm17690	predicted gene, 17690	2.27	0.00
ENSMUSG00000022074	Tnfrsf10b	TNF receptor superfamily member 10a	2.27	0.00
ENSMUSG00002075890	Gm56433	predicted gene, 56433	2.28	0.00
ENSMUSG00000038564	Ift172	intraflagellar transport 172	2.28	0.01
ENSMUSG00000024999	Noc3l	NOC3 like DNA replication regulator	2.28	0.03
ENSMUSG00000042788	Cimip2b	ciliary microtubule inner protein 2B	2.28	0.00
ENSMUSG00000050315	Synpo2	synaptopodin 2	2.28	0.01
ENSMUSG00000120367	Gm57294	predicted gene, 57294	2.28	0.00
ENSMUSG00000001525	Tubb5	tubulin beta class I	2.29	0.00
ENSMUSG00000032737	Inppl1	inositol polyphosphate phosphatase like 1	2.29	0.00
ENSMUSG00000041841	Rpl37	ribosomal protein L37	2.29	0.02
ENSMUSG00000063015	Ccni	cyclin I	2.29	0.00
ENSMUSG00000097675	1700101I11Rik	RIKEN cDNA 1700101I11 gene	2.29	0.00
ENSMUSG00000033088	Triobp	TRIO and F-actin binding protein	2.29	0.02
ENSMUSG00000120615	Gm56802	predicted gene, 56802	2.29	0.00
ENSMUSG00000115049	Gm49172	predicted gene, 49172	2.29	0.00
ENSMUSG00000109452	Gm44530	predicted gene 44530	2.29	0.03
ENSMUSG00000030788	Rnf141	ring finger protein 141	2.30	0.00
ENSMUSG00000048763	Hoxb3	homeobox B3	2.30	0.00
ENSMUSG00000049659	Aftph	aftiphilin	2.30	0.05
ENSMUSG00000092741	Mir3074-1	microRNA 3074	2.30	0.00
ENSMUSG00000061787	Rps17	ribosomal protein S17	2.31	0.02
ENSMUSG00000091906	1700099I09Rik	RIKEN cDNA 1700099I09 gene	2.31	0.00
ENSMUSG00000024240	Epc1	enhancer of polycomb homolog 1	2.31	0.01
ENSMUSG00000118489	Gm53038	predicted gene, 53038	2.31	0.00
ENSMUSG00000029071	Dvl1	dishevelled segment polarity protein 1	2.31	0.00
ENSMUSG00000038412	Higd1a	HIG1 hypoxia inducible domain family member 1A	2.31	0.05
ENSMUSG00000000959	Oxa1l	OXA1L mitochondrial inner membrane protein	2.31	0.00
ENSMUSG00000025128	Bhlhe22	basic helix-loop-helix family member e22	2.31	0.00
ENSMUSG00000007041	Clic1	chloride intracellular channel 1	2.31	0.02
ENSMUSG00000032601	Prkar2a	protein kinase cAMP-dependent type II regulatory subunit alpha	2.31	0.00
ENSMUSG00000029603	Dtx1	deltex E3 ubiquitin ligase 1	2.31	0.01
ENSMUSG00000072704	Smim10l1	small integral membrane protein 10 like 1	2.32	0.00
ENSMUSG00000036560	Lgi4	leucine rich repeat LGI family member 4	2.32	0.01
ENSMUSG00000034928	Rnf44	ring finger protein 44	2.32	0.00
ENSMUSG00000097471	5830432E09Rik	RIKEN cDNA 5830432E09 gene	2.32	0.00
ENSMUSG00000028955	Vamp3	vesicle associated membrane protein 3	2.33	0.03
ENSMUSG00000037742	Eef1a1	eukaryotic translation elongation factor 1 alpha 1	2.33	0.00
ENSMUSG00000000957	Mmp14	matrix metallopeptidase 14	2.33	0.01
ENSMUSG00000037257	Aagab	alpha and gamma adaptin binding protein	2.33	0.01
ENSMUSG00000071454	Dtnb	dystrobrevin beta	2.33	0.04
ENSMUSG00000015745	Plekho1	pleckstrin homology domain containing O1	2.34	0.00
ENSMUSG00000004665	Cnn2	calponin 2	2.34	0.02
ENSMUSG00000025290	Rps24	ribosomal protein S24	2.34	0.00
ENSMUSG00000079435	Rpl36a	ribosomal protein L36A	2.34	0.00

Essemble ID	Gene Symbol	Gene Description	Fold Change	P Value
ENSMUSG00000031397	Tktl1	transketolase like 1	2.34	0.02
ENSMUSG00000026646	Suv39h2	SUV39H2 histone lysine methyltransferase	2.34	0.01
ENSMUSG00000110637	Gm7807	ribosomal protein S7 pseudogene	2.34	0.01
ENSMUSG00000089707	Slain1os	SLAIN motif family, member 1, opposite strand	2.34	0.01
ENSMUSG00000061972	Or1o4	olfactory receptor family 1 subfamily O member 4	2.35	0.01
ENSMUSG00000028799	Zfp362	zinc finger protein 362	2.35	0.05
ENSMUSG00000030609	Aen	apoptosis enhancing nuclease	2.35	0.00
ENSMUSG00000053965	Pde5a	phosphodiesterase 5A	2.35	0.00
ENSMUSG00000015970	Chdh	choline dehydrogenase	2.35	0.01
ENSMUSG00000104535	Gm42686	predicted gene 42686	2.35	0.00
ENSMUSG00000030530	Furin	furin, paired basic amino acid cleaving enzyme	2.35	0.00
ENSMUSG00000073152	Gm10475	predicted gene 10475	2.35	0.00
ENSMUSG00000096938	9530052E02Rik	RIKEN cDNA 9530052E02 gene	2.36	0.00
ENSMUSG00000096606	Tpbgl	trophoblast glycoprotein like	2.36	0.01
ENSMUSG00000091119	Ccdc152	coiled-coil domain containing 152	2.36	0.00
ENSMUSG00000000276	Dgke	diacylglycerol kinase epsilon	2.36	0.01
ENSMUSG00000025241	Fyco1	FYVE and coiled-coil domain autophagy adaptor 1	2.36	0.01
ENSMUSG00000062270	Morf4l1	mortality factor 4 like 1	2.36	0.01
ENSMUSG00000001506	Col1a1	collagen type I alpha 1 chain	2.36	0.01
ENSMUSG00000039599	Fam149b	family with sequence similarity 149 member B1	2.36	0.00
ENSMUSG00000086360	Gm16214	predicted gene 16214	2.36	0.00
ENSMUSG00000087231	E230016M11Rik	RIKEN cDNA E230016M11 gene	2.36	0.00
ENSMUSG00000042759	Apobr	apolipoprotein B receptor	2.37	0.00
ENSMUSG00000085645	Hoxb5os	homeobox B5 and homeobox B6, opposite strand	2.37	0.00
ENSMUSG00000100039	Gm28959	predicted gene 28959	2.37	0.00
ENSMUSG00000085546	Gm14252	predicted gene 14252	2.37	0.00
ENSMUSG00000031487	Brf2	BRF2 RNA polymerase III transcription initiation factor subunit	2.37	0.00
ENSMUSG00000019768	Esr1	estrogen receptor 1	2.37	0.01
ENSMUSG00000033054	Npat	nuclear protein, coactivator of histone transcription	2.37	0.02
ENSMUSG00000028796	Phc2	polyhomeotic homolog 2	2.37	0.01
ENSMUSG00000024993	Dennd10	DENN domain containing 10	2.38	0.01
ENSMUSG00000044636	Csrnp2	cysteine and serine rich nuclear protein 2	2.38	0.00
ENSMUSG00000108709	4933431G14Rik	RIKEN cDNA 4933431G14 gene	2.38	0.02
ENSMUSG00000101451	Gm29430	predicted gene 29430	2.38	0.02
ENSMUSG00000079333	Gm11096	predicted gene 11096	2.38	0.00
ENSMUSG00000086444	Gm15961	predicted gene 15961	2.38	0.01
ENSMUSG00000034994	Eef2	eukaryotic translation elongation factor 2	2.39	0.01
ENSMUSG00000091659	Gm17099	predicted gene 17099	2.39	0.01
ENSMUSG00000005481	Ddx39a	DExD-box helicase 39A	2.39	0.00
ENSMUSG00000034271	Jdp2	Jun dimerization protein 2	2.39	0.03
ENSMUSG00000021866	Anxa11	annexin A11	2.39	0.02
ENSMUSG00000003970	Rpl8	ribosomal protein L8	2.40	0.00
ENSMUSG00000117814	Gm50431	predicted gene, 50431	2.40	0.00
ENSMUSG00000043259	Fam13c	family with sequence similarity 13 member C	2.40	0.04
ENSMUSG00000041417	Pik3r1	phosphoinositide-3-kinase regulatory subunit 1	2.40	0.01
ENSMUSG00000023259	Slc26a6	solute carrier family 26 member 6	2.40	0.00

Essemble ID	Gene Symbol	Gene Description	Fold Change	P Value
ENSMUSG00000086953	Aknaos	AT-hook transcription factor, opposite strand	2.40	0.00
ENSMUSG00000055322	Tns1	tensin 1	2.41	0.00
ENSMUSG00000039741	Bahcc1	BAH domain and coiled-coil containing 1	2.41	0.00
ENSMUSG00000037060	Cavin3	caveolae associated protein 3	2.41	0.00
ENSMUSG00000037003	Tns2	tensin 2	2.41	0.00
ENSMUSG00000053714	4732471J01Rik	RIKEN cDNA 4732471J01 gene	2.41	0.00
ENSMUSG00000066406	Akap13	A-kinase anchoring protein 13	2.41	0.00
ENSMUSG00000099966	2810402E24Rik	RIKEN cDNA 2810402E24 gene	2.42	0.00
ENSMUSG00000081343	Gm16071	ribosomal protein L7A pseudogene	2.42	0.00
ENSMUSG00000062127	Cttnbp2nl	CTTNBP2 N-terminal like	2.42	0.00
ENSMUSG00000090841	Myl6	myosin light chain 6	2.42	0.00
ENSMUSG00000035569	Ankrd11	ankyrin repeat domain containing 11	2.42	0.00
ENSMUSG00000025223	Ldb1	LIM domain binding 1	2.42	0.00
ENSMUSG00000042766	Trim46	tripartite motif containing 46	2.42	0.00
ENSMUSG00000084863	Gm12523	predicted gene 12523	2.42	0.00
ENSMUSG00000042359	Osbpl6	oxysterol binding protein like 6	2.42	0.00
ENSMUSG00000026991	Pkp4	plakophilin 4	2.43	0.04
ENSMUSG00000019822	Smpd2	sphingomyelin phosphodiesterase 2	2.43	0.00
ENSMUSG00000041120	Nbl1	NBL1, DAN family BMP antagonist	2.43	0.00
ENSMUSG00000073985	Gm10602	predicted gene 10602	2.43	0.00
ENSMUSG00000027427	Polr3f	RNA polymerase III subunit F	2.43	0.00
ENSMUSG00000039787	Cercam	cerebral endothelial cell adhesion molecule	2.43	0.00
ENSMUSG00000039047	Pigk	phosphatidylinositol glycan anchor biosynthesis class K	2.43	0.01
ENSMUSG00000028082	Sh3d19	SH3 domain containing 19	2.44	0.04
ENSMUSG00000097098	9330111N05Rik	RIKEN cDNA 9330111N05 gene	2.44	0.02
ENSMUSG00000031486	Adgra2	adhesion G protein-coupled receptor A2	2.44	0.00
ENSMUSG00000028339	Col15a1	collagen type XV alpha 1 chain	2.44	0.02
ENSMUSG00000117413	Gm50044	predicted gene, 50044	2.44	0.00
ENSMUSG00000084803	5830444B04Rik	RIKEN cDNA 5830444B04 gene	2.45	0.00
ENSMUSG00000113471	Gm48116	predicted gene, 48116	2.45	0.01
ENSMUSG00000027569	Mrgbp	MRG domain binding protein	2.45	0.00
ENSMUSG00000025145	Lrrc45	leucine rich repeat containing 45	2.45	0.05
ENSMUSG00000003039	Fam32a	family with sequence similarity 32 member A	2.46	0.03
ENSMUSG00000021477	Ctsl	cathepsin V	2.46	0.00
ENSMUSG00000086719	Gm16275	predicted gene 16275	2.46	0.01
ENSMUSG00000085080	2310044K18Rik	RIKEN cDNA 2310044K18 gene	2.46	0.02
ENSMUSG00000087690	Gm16031	predicted gene 16031	2.46	0.00
ENSMUSG00000015932	Dstn	destrin, actin depolymerizing factor	2.46	0.02
ENSMUSG00000001418	Glmp	glycosylated lysosomal membrane protein	2.46	0.00
ENSMUSG00000066568	Lsm14a	LSM14A mRNA processing body assembly factor	2.46	0.00
ENSMUSG00000112925	Gm31763	predicted gene, 31763	2.47	0.00
ENSMUSG00000025646	Atrip	ATR interacting protein	2.47	0.00
ENSMUSG00000028229	Rmdn1	regulator of microtubule dynamics 1	2.47	0.00
ENSMUSG00000003154	Foxj2	forkhead box J2	2.47	0.00
ENSMUSG00000086395	A630014C17Rik	RIKEN cDNA A630014C17 gene	2.47	0.00
ENSMUSG00000013236	Ptprs	protein tyrosine phosphatase receptor type S	2.47	0.03

Essemble ID	Gene Symbol	Gene Description	Fold Change	P Value
ENSMUSG00000020775	Mrpl38	mitochondrial ribosomal protein L38	2.48	0.00
ENSMUSG00000036606	Plxnb2	plexin B2	2.48	0.01
ENSMUSG00000029455	Aldh2	aldehyde dehydrogenase 2 family member	2.48	0.00
ENSMUSG00000068220	Lgals1	galectin 1	2.48	0.00
ENSMUSG00000019461	Plscr3	phospholipid scramblase 3	2.48	0.00
ENSMUSG00000041309	Nkx6-2	NK6 homeobox 2	2.48	0.00
ENSMUSG00000118445	Gm50469	predicted gene, 50469	2.49	0.00
ENSMUSG00000052920	Prkg1	protein kinase cGMP-dependent 1	2.49	0.05
ENSMUSG00000022389	Tef	TEF transcription factor, PAR bZIP family member	2.49	0.04
ENSMUSG00000120299	Gm56490	predicted gene, 56490	2.49	0.00
ENSMUSG00000037012	Hk1	hexokinase 1	2.49	0.00
ENSMUSG00000116946	Gm41442	predicted gene, 41442	2.49	0.00
ENSMUSG00000041075	Fzd7	frizzled class receptor 7	2.49	0.02
ENSMUSG00000097788	Gm16596	predicted gene, 16596	2.49	0.00
ENSMUSG00000017404	Rpl19	ribosomal protein L19	2.49	0.00
ENSMUSG00000033128	Gga1	golgi associated, gamma adaptin ear containing, ARF binding protein 1	2.49	0.00
ENSMUSG00000051790	Nlgn2	neuroligin 2	2.50	0.01
ENSMUSG00000054021	Sirt5	sirtuin 5	2.51	0.00
ENSMUSG00000026504	Sdccag8	SHH signaling and ciliogenesis regulator SDCCAG8	2.51	0.00
ENSMUSG00000038593	Tctn1	tectonic family member 1	2.51	0.00
ENSMUSG00000048279	Sacs	sacsin	2.51	0.00
ENSMUSG00000119960	Gm30639	predicted gene, 30639	2.52	0.00
ENSMUSG00000022010	Tsc22d1	TSC22 domain family member 1	2.52	0.05
ENSMUSG00000038540	Tmc3	transmembrane channel like 3	2.53	0.00
ENSMUSG00000117391	4930500A05Rik	RIKEN cDNA 4930500A05 gene	2.53	0.00
ENSMUSG00000058600	Rpl30	ribosomal protein L30	2.54	0.02
ENSMUSG00000001786	Fbxo7	F-box protein 7	2.55	0.00
ENSMUSG00000120746	Gm56841	predicted gene, 56841	2.55	0.01
ENSMUSG00000107458	Gm44040	predicted gene, 44040	2.55	0.00
ENSMUSG00000035929	H2-Q4	major histocompatibility complex, class I, A	2.55	0.00
ENSMUSG00000037747	Phyhipl	phytanoyl-CoA 2-hydroxylase interacting protein like	2.55	0.00
ENSMUSG00000024036	Slc37a1	solute carrier family 37 member 1	2.55	0.05
ENSMUSG00000035873	Pawr	pro-apoptotic WT1 regulator	2.55	0.01
ENSMUSG00000029426	Scarb2	scavenger receptor class B member 2	2.55	0.04
ENSMUSG00000000486	Septin1	septin 1	2.55	0.01
ENSMUSG00000022475	Hdac7	histone deacetylase 7	2.56	0.00
ENSMUSG00000026730	Pter	phosphotriesterase related	2.56	0.01
ENSMUSG00000030336	Cd27	CD27 molecule	2.56	0.00
ENSMUSG00000004530	Coro1c	coronin 1C	2.56	0.00
ENSMUSG00000030805	Stx4a	syntaxin 4	2.56	0.00
ENSMUSG00000022286	Grhl2	grainyhead like transcription factor 2	2.56	0.01
ENSMUSG00000077450	Rab11b	RAB11B, member RAS oncogene family	2.57	0.00
ENSMUSG00000018217	Pmp22	peripheral myelin protein 22	2.57	0.03
ENSMUSG00000100486	Gm4131	predicted gene 4131	2.57	0.00
ENSMUSG00000052684	Jun	Jun proto-oncogene, AP-1 transcription factor subunit	2.57	0.02
ENSMUSG00000029050	Ski	SKI proto-oncogene	2.58	0.04

Essemble ID	Gene Symbol	Gene Description	Fold Change	P Value
ENSMUSG00000063457	Rps15	ribosomal protein S15	2.58	0.01
ENSMUSG00000085057	Gm13415	predicted gene 13415	2.58	0.00
ENSMUSG00000114136	4921511I17Rik	RIKEN cDNA 4921511I17 gene	2.58	0.00
ENSMUSG00000049295	Zfp219	zinc finger protein 219	2.58	0.00
ENSMUSG00000086043	Gm12473	predicted gene 12473	2.58	0.00
ENSMUSG00000086245	Gm16170	predicted gene 16170	2.58	0.00
ENSMUSG00000109847	Gm45278	predicted gene 45278	2.59	0.00
ENSMUSG00000041354	Rgl2	ral guanine nucleotide dissociation stimulator like 2	2.59	0.00
ENSMUSG00000074203	G430095P16Rik	RIKEN cDNA G430095P16 gene	2.59	0.00
ENSMUSG00000041791	Capza3	capping actin protein of muscle Z-line subunit alpha 3	2.59	0.05
ENSMUSG00000030801	Kat8	lysine acetyltransferase 8	2.59	0.01
ENSMUSG00000032698	Lmo2	LIM domain only 2	2.59	0.00
ENSMUSG00000022863	Btg3	BTG anti-proliferation factor 3	2.59	0.00
ENSMUSG00000035726	Supt16	SPT16 homolog, facilitates chromatin remodeling subunit	2.60	0.00
ENSMUSG00000032334	Loxl1	lysyl oxidase like 1	2.60	0.01
ENSMUSG00000025856	Pdgfa	platelet derived growth factor subunit A	2.60	0.00
ENSMUSG00000020303	Stc2	stanniocalcin 2	2.61	0.00
ENSMUSG00000098973	Mir6236	microRNA 6236	2.61	0.00
ENSMUSG00000049653	Spatc1	spermatogenesis and centriole associated 1	2.61	0.02
ENSMUSG00000089804	Gm16136	predicted gene 16136	2.61	0.00
ENSMUSG00000074818	Pdzd7	PDZ domain containing 7	2.61	0.00
ENSMUSG00000091393	5330438I03Rik	RIKEN cDNA 5330438I03 gene	2.61	0.01
ENSMUSG00000040532	Abhd11	abhydrolase domain containing 11	2.61	0.00
ENSMUSG00000021690	Jmy	junction mediating and regulatory protein, p53 cofactor	2.61	0.03
ENSMUSG00000106583	4930447N08Rik	RIKEN cDNA 4930447N08 gene	2.61	0.00
ENSMUSG00000002833	Hdgfl2	HDGF like 2	2.61	0.01
ENSMUSG00000035495	Tstd2	thiosulfate sulfurtransferase like domain containing 2	2.61	0.01
ENSMUSG00000035969	Rusc2	RUN and SH3 domain containing 2	2.62	0.04
ENSMUSG00000046876	Atxn1	ataxin 1	2.63	0.00
ENSMUSG00000046408	1700067K01Rik	chromosome 19 open reading frame 67	2.63	0.00
ENSMUSG00000018160	Med1	mediator complex subunit 1	2.63	0.00
ENSMUSG00000092365	BC023719	ribosomal protein S6 pseudogene	2.63	0.00
ENSMUSG00000033712	Ccar2	cell cycle and apoptosis regulator 2	2.63	0.00
ENSMUSG00000074228	Gm10645	predicted gene 10645	2.63	0.00
ENSMUSG00000120544	Gm57081	predicted gene, 57081	2.64	0.01
ENSMUSG00000072962	Gm16401	predicted gene 16401	2.64	0.00
ENSMUSG00000055675	Kbtbd11	kelch repeat and BTB domain containing 11	2.64	0.00
ENSMUSG00000118340	Gm50301	predicted gene, 50301	2.65	0.00
ENSMUSG00000097729	2310015A10Rik	RIKEN cDNA 2310015A10 gene	2.65	0.00
ENSMUSG00000024269	Tpgs2	tubulin polyglutamylase complex subunit 2	2.65	0.04
ENSMUSG00000029009	Mthfr	methylenetetrahydrofolate reductase	2.65	0.00
ENSMUSG00000038886	Man2a2	mannosidase alpha class 2A member 2	2.66	0.00
ENSMUSG00000120788	Gm36393	predicted gene, 36393	2.66	0.00
ENSMUSG00000075256	Cerkl	ceramide kinase like	2.66	0.00
ENSMUSG00000001802	Lrp3	LDL receptor related protein 3	2.66	0.00
ENSMUSG00000111856	6530413G14Rik	RIKEN cDNA 6530413G14 gene	2.66	0.00

Essemble ID	Gene Symbol	Gene Description	Fold Change	P Value
ENSMUSG00000024985	Tcf7l2	transcription factor 7 like 2	2.66	0.00
ENSMUSG00000078794	Dact3	dishevelled binding antagonist of beta catenin 3	2.66	0.00
ENSMUSG00000022518	4930562C15Rik	chromosome 16 open reading frame 96	2.67	0.00
ENSMUSG00000008682	Rpl10	ribosomal protein L10	2.67	0.02
ENSMUSG00000087399	Gm11899	predicted gene 11899	2.68	0.00
ENSMUSG00000022610	Mapk12	mitogen-activated protein kinase 12	2.68	0.00
ENSMUSG00000022179	4931414P19Rik	chromosome 14 open reading frame 93	2.68	0.00
ENSMUSG00000112110	Gm15608	predicted gene 15608	2.69	0.00
ENSMUSG00000102349	Gm37376	predicted gene, 37376	2.69	0.00
ENSMUSG00000086746	Gm15222	predicted gene 15222	2.69	0.00
ENSMUSG00000001288	Rarg	retinoic acid receptor gamma	2.69	0.01
ENSMUSG00000028978	Nos3	nitric oxide synthase 3	2.69	0.00
ENSMUSG00000029580	Actb	actin beta	2.70	0.01
ENSMUSG00000097636	Mirt1	myocardial infarction associated transcript 1	2.70	0.01
ENSMUSG00000092550	Gm20496	predicted gene 20496	2.70	0.00
ENSMUSG00000101671	Gm28221	predicted gene 28221	2.70	0.00
ENSMUSG00000113165	Gm47863	predicted gene, 47863	2.70	0.00
ENSMUSG00000028161	Ppp3ca	protein phosphatase 3 catalytic subunit alpha	2.71	0.01
ENSMUSG00000032562	Gnai2	G protein subunit alpha i2	2.71	0.00
ENSMUSG00000040703	Cyp2s1	cytochrome P450 family 2 subfamily S member 1	2.71	0.01
ENSMUSG00000025202	Scd3	stearoyl-coenzyme A desaturase 3	2.71	0.00
ENSMUSG00000110291	Gm37419	predicted gene, 37419	2.71	0.00
ENSMUSG00000021130	Galnt16	polypeptide N-acetylgalactosaminyltransferase 16	2.72	0.01
ENSMUSG00000025650	Col7a1	collagen type VII alpha 1 chain	2.72	0.01
ENSMUSG00000086606	Gm13205	predicted gene 13205	2.72	0.00
ENSMUSG00000081282	Gm13836	40S ribosomal protein S5 pseudogene	2.73	0.02
ENSMUSG00000033161	Atp1a1	ATPase Na+/K+ transporting subunit alpha 1	2.73	0.04
ENSMUSG00000019564	Arid3a	AT-rich interaction domain 3A	2.73	0.01
ENSMUSG00000114860	Gm49291	predicted gene, 49291	2.73	0.00
ENSMUSG00000117676	Gm50314	predicted gene, 50314	2.73	0.02
ENSMUSG00000090054	Gm15629	predicted gene 15629	2.73	0.00
ENSMUSG00000104093	A330015K06Rik	RIKEN cDNA A330015K06 gene	2.74	0.00
ENSMUSG00000025083	Afap1l2	actin filament associated protein 1 like 2	2.74	0.01
ENSMUSG00000019470	Xab2	XPA binding protein 2	2.74	0.00
ENSMUSG00000038121	Fam210a	family with sequence similarity 210 member A	2.74	0.00
ENSMUSG00000060419	Rps16-ps2	ribosomal protein S16, pseudogene 2	2.74	0.00
ENSMUSG00000114705	Gm18760	corepressor interacting with RBPJ, 1 pseudogene	2.75	0.00
ENSMUSG00000047675	Rps8	ribosomal protein S8	2.75	0.00
ENSMUSG00000021939	Ctsb	cathepsin B	2.76	0.00
ENSMUSG00000010663	Fads1	fatty acid desaturase 1	2.76	0.00
ENSMUSG00000038872	Zfhx3	zinc finger homeobox 3	2.76	0.00
ENSMUSG00000035235	Trim13	tripartite motif containing 13	2.76	0.00
ENSMUSG00000015659	Serac1	serine active site containing 1	2.76	0.00
ENSMUSG00000062980	Cped1	cadherin like and PC-esterase domain containing 1	2.76	0.00
ENSMUSG00000044125	9530080O11Rik	RIKEN cDNA 9530080O11 gene	2.76	0.01
ENSMUSG00000086867	Gm4577	predicted gene 4577	2.76	0.01

Essemble ID	Gene Symbol	Gene Description	Fold Change	P Value
ENSMUSG00000022906	Parp9	poly(ADP-ribose) polymerase family member 9	2.77	0.00
ENSMUSG00000040536	Necab1	N-terminal EF-hand calcium binding protein 1	2.77	0.00
ENSMUSG00000031714	Gab1	GRB2 associated binding protein 1	2.77	0.00
ENSMUSG00000031445	Proz	protein Z, vitamin K dependent plasma glycoprotein	2.78	0.00
ENSMUSG00000087524	Gm14285	predicted gene 14285	2.78	0.00
ENSMUSG00000114382	Gm4814	predicted gene 4814	2.78	0.01
ENSMUSG00000020196	Cabin1	calcineurin binding protein 1	2.78	0.01
ENSMUSG00000001036	Epn2	epsin 2	2.78	0.00
ENSMUSG00000038457	Tmem255b	transmembrane protein 255B	2.78	0.00
ENSMUSG00000038793	Lefty1	left-right determination factor 1	2.78	0.00
ENSMUSG00000028034	Fubp1	far upstream element binding protein 1	2.78	0.00
ENSMUSG00000021171	Esyt2	extended synaptotagmin 2	2.79	0.03
ENSMUSG00000044052	Ccr10	C-C motif chemokine receptor 10	2.79	0.00
ENSMUSG00000039684	Gm5422	proteasome (prosome, macropain) 26S subunit, non-ATPase, 2 pseudogene	2.79	0.02
ENSMUSG00000119584	Rn18s-rs5	18s RNA, related sequence 5	2.80	0.01
ENSMUSG00000116712	Gm49598	predicted gene, 49598	2.80	0.03
ENSMUSG00000051339	2900026A02Rik	RIKEN cDNA 2900026A02 gene	2.80	0.03
ENSMUSG00000017412	Cacnb4	calcium voltage-gated channel auxiliary subunit beta 4	2.80	0.00
ENSMUSG00000018593	Sparc	secreted protein acidic and cysteine rich	2.80	0.01
ENSMUSG00000089713	Gm16564	predicted gene 16564	2.80	0.00
ENSMUSG00000051413	Plagl2	PLAG1 like zinc finger 2	2.81	0.00
ENSMUSG00000106112	Gm43434	predicted gene 43434	2.81	0.00
ENSMUSG00000046168	Kcnrg	potassium channel regulator	2.81	0.00
ENSMUSG00000092368	A930015D03Rik	RIKEN cDNA A930015D03 gene	2.81	0.00
ENSMUSG00000027357	Crls1	cardiolipin synthase 1	2.82	0.05
ENSMUSG00000024190	Dusp1	dual specificity phosphatase 1	2.82	0.00
ENSMUSG00000114968	A630019I02Rik	RIKEN cDNA A630019I02 gene	2.83	0.00
ENSMUSG00000030403	Vasp	vasodilator stimulated phosphoprotein	2.84	0.00
ENSMUSG00000120003	Gm57201	predicted gene, 57201	2.84	0.00
ENSMUSG00000108219	Gm44101	predicted gene, 44101	2.84	0.00
ENSMUSG00000032060	Cryab	crystallin alpha B	2.84	0.00
ENSMUSG00000109511	Nup62	nucleoporin 62	2.85	0.01
ENSMUSG00000035382	Pcsk7	proprotein convertase subtilisin/kexin type 7	2.85	0.00
ENSMUSG00000112950	Gm32369	predicted gene, 32369	2.85	0.00
ENSMUSG00000106678	Gm43457	predicted gene 43457	2.85	0.01
ENSMUSG00000050556	Kcnb1	potassium voltage-gated channel subfamily B member 1	2.86	0.00
ENSMUSG00000028936	Rpl22	ribosomal protein L22	2.86	0.01
ENSMUSG00000073096	Lrrc61	leucine rich repeat containing 61	2.86	0.00
ENSMUSG00000030493	Faap24	FA core complex associated protein 24	2.86	0.00
ENSMUSG00000019907	Ppp1r12a	protein phosphatase 1 regulatory subunit 12A	2.86	0.00
ENSMUSG00000014837	Matcap1	microtubule associated tyrosine carboxypeptidase 1	2.87	0.00
ENSMUSG00000044783	Hjurp	Holliday junction recognition protein	2.87	0.00
ENSMUSG00000000958	Slc7a7	solute carrier family 7 member 7	2.87	0.00
ENSMUSG00000058297	Spock2	SPARC (osteonectin), cwcv and kazal like domains proteoglycan 2	2.87	0.02
ENSMUSG00000064373	Selenop	selenoprotein P	2.87	0.00
ENSMUSG00000029675	Eln	elastin	2.88	0.05

Essemble ID	Gene Symbol	Gene Description	Fold Change	P Value
ENSMUSG00000026223	Itm2c	integral membrane protein 2C	2.88	0.00
ENSMUSG00000096957	E230013L22Rik	RIKEN cDNA E230013L22 gene	2.88	0.00
ENSMUSG00000030400	Ercc2	ERCC excision repair 2, TFIIH core complex helicase subunit	2.88	0.00
ENSMUSG00000111447	Gm48249	predicted gene, 48249	2.88	0.00
ENSMUSG00000022591	Gm9747	predicted gene 9747	2.89	0.00
ENSMUSG00000112841	Gm48752	predicted gene, 48752	2.89	0.00
ENSMUSG00000057069	Ero1b	endoplasmic reticulum oxidoreductase 1 beta	2.89	0.00
ENSMUSG00000043795	Prr33	proline rich 33	2.89	0.00
ENSMUSG00000108158	Gm44002	predicted gene, 44002	2.89	0.00
ENSMUSG00000034041	Lyl1	LYL1 basic helix-loop-helix family member	2.90	0.00
ENSMUSG00000044701	Il27	interleukin 27	2.90	0.00
ENSMUSG00000100465	Gm29264	predicted gene 29264	2.90	0.01
ENSMUSG00000002844	Adprh	ADP-ribosylarginine hydrolase	2.90	0.04
ENSMUSG00000025212	Sfxn3	sideroflexin 3	2.90	0.01
ENSMUSG00000020542	Myocd	myocardin	2.90	0.03
ENSMUSG00000105458	Mir3074-2	microRNA 3074	2.91	0.01
ENSMUSG00000071547	Nt5dc2	5'-nucleotidase domain containing 2	2.91	0.00
ENSMUSG00000020160	Meis1	Meis homeobox 1	2.91	0.00
ENSMUSG00000021224	Numb	NUMB endocytic adaptor protein	2.91	0.01
ENSMUSG00000027931	Npr1	natriuretic peptide receptor 1	2.91	0.00
ENSMUSG00000026193	Fn1	fibronectin 1	2.92	0.00
ENSMUSG00000034664	Itga2b	integrin subunit alpha 2b	2.92	0.00
ENSMUSG00000036225	Kctd1	potassium channel tetramerization domain containing 1	2.92	0.00
ENSMUSG00000026207	Speg	striated muscle enriched protein kinase	2.92	0.00
ENSMUSG00000110545	Gm7730	nucleoside diphosphate kinase B pseudogene	2.92	0.02
ENSMUSG00000040463	Mybbp1a	MYB binding protein 1a	2.92	0.00
ENSMUSG00000096962	Gm26622	predicted gene, 26622	2.92	0.01
ENSMUSG00000019055	Plod1	procollagen-lysine,2-oxoglutarate 5-dioxygenase 1	2.92	0.02
ENSMUSG00000079261	Gm15217	predicted gene 15217	2.93	0.00
ENSMUSG00000092341	Malat1	metastasis associated lung adenocarcinoma transcript 1	2.93	0.01
ENSMUSG00000103502	9330121J05Rik	RIKEN cDNA 9330121J05 gene	2.95	0.00
ENSMUSG00000024816	Frmd8	FERM domain containing 8	2.95	0.00
ENSMUSG00000026222	Sp100	nuclear antigen Sp100	2.95	0.00
ENSMUSG00000021874	4933413J09Rik	RIKEN cDNA 4933413J09 gene	2.95	0.00
ENSMUSG00000086308	G630016G05Rik	RIKEN cDNA G630016G05 gene	2.95	0.00
ENSMUSG00000020687	Cdc27	cell division cycle 27	2.95	0.00
ENSMUSG00000024644	Cndp2	carnosine dipeptidase 2	2.96	0.03
ENSMUSG00000109876	Gm45449	predicted gene 45449	2.96	0.00
ENSMUSG00000093954	Gm16867	predicted gene, 16867	2.96	0.05
ENSMUSG00000006205	Htra1	HtrA serine peptidase 1	2.96	0.01
ENSMUSG00000054006	D630008O14Rik	RIKEN cDNA D630008O14 gene	2.97	0.00
ENSMUSG00000038866	Zcchc2	zinc finger CCHC-type containing 2	2.97	0.01
ENSMUSG00000117000	Gm32509	predicted gene, 32509	2.97	0.02
ENSMUSG00000020473	Aebp1	AE binding protein 1	2.98	0.00
ENSMUSG00000097224	Gm26716	predicted gene, 26716	2.98	0.00
ENSMUSG00000036686	Cc2d1a	coiled-coil and C2 domain containing 1A	2.98	0.01

Essemble ID	Gene Symbol	Gene Description	Fold Change	P Value
ENSMUSG00000074141	Il4i1	interleukin 4 induced 1	2.99	0.00
ENSMUSG00000086266	Igf2os	IGF2 antisense RNA	2.99	0.01
ENSMUSG00000107379	Gm43126	predicted gene 43126	2.99	0.00
ENSMUSG00000100980	Gm29100	predicted gene 29100	2.99	0.00
ENSMUSG00000048747	E130114P18Rik	RIKEN cDNA E130114P18 gene	2.99	0.00
ENSMUSG00000036244	Tbc1d21	TBC1 domain family member 21	3.00	0.01
ENSMUSG00000050675	Gp1ba	glycoprotein Ib platelet subunit alpha	3.00	0.00
ENSMUSG00000106232	Gm43236	predicted gene 43236	3.00	0.01
ENSMUSG00000015149	Sirt2	sirtuin 2	3.00	0.00
ENSMUSG00000013419	Zbtb47	zinc finger and BTB domain containing 47	3.01	0.00
ENSMUSG00000061232	H2-K1	major histocompatibility complex, class I, A	3.01	0.00
ENSMUSG00000025422	Agap2	ArfGAP with GTPase domain, ankyrin repeat and PH domain 2	3.01	0.00
ENSMUSG00000074457	S100a16	S100 calcium binding protein A16	3.01	0.04
ENSMUSG00000120308	Gm56915	predicted gene, 56915	3.01	0.00
ENSMUSG00000021493	Pdlim7	PDZ and LIM domain 7	3.02	0.00
ENSMUSG00000085762	Lrrc75aos2	leucine rich repeat containing 75A, opposite strand 2	3.02	0.00
ENSMUSG00000097819	Gm26813	predicted gene, 26813	3.03	0.00
ENSMUSG00000038025	Phf2	PHD finger protein 2	3.03	0.00
ENSMUSG00000038393	Txnip	thioredoxin interacting protein	3.03	0.01
ENSMUSG00000028337	Coro2a	coronin 2A	3.03	0.01
ENSMUSG00000003847	Nfat5	nuclear factor of activated T cells 5	3.03	0.00
ENSMUSG00000102368	4930590L20Rik	RIKEN cDNA 4930590L20 gene	3.04	0.00
ENSMUSG00000037108	Zcwpw1	zinc finger CW-type and PWWP domain containing 1	3.05	0.04
ENSMUSG00000038664	Herc1	HECT and RLD domain containing E3 ubiquitin protein ligase family member 1	3.05	0.00
ENSMUSG00000118667	Ahnak2	AHNAK nucleoprotein 2	3.05	0.01
ENSMUSG00000110166	Gm45641	predicted gene 45641	3.06	0.00
ENSMUSG00000026245	Farsb	phenylalanyl-tRNA synthetase subunit beta	3.06	0.00
ENSMUSG00000025407	Gli1	GLI family zinc finger 1	3.06	0.00
ENSMUSG00000030317	Timp4	TIMP metallopeptidase inhibitor 4	3.06	0.01
ENSMUSG00000047485	Klhl34	kelch like family member 34	3.06	0.00
ENSMUSG00000091373	Gm8810	predicted gene 8810	3.06	0.00
ENSMUSG00000085595	Gm16090	predicted gene 16090	3.07	0.00
ENSMUSG00000087291	Gm11946	predicted gene 11946	3.07	0.00
ENSMUSG00000039485	Tspyl4	TSPY like 4	3.08	0.00
ENSMUSG00000085624	Gm14573	predicted gene 14573	3.08	0.00
ENSMUSG00000120070	Gm56719	predicted gene, 56719	3.08	0.00
ENSMUSG00000003429	Rps11	ribosomal protein S11	3.09	0.00
ENSMUSG00000059742	Kcnh7	potassium voltage-gated channel subfamily H member 7	3.09	0.00
ENSMUSG00000032300	1700017B05Rik	chromosome 15 open reading frame 39	3.09	0.02
ENSMUSG00000107682	Gm33024	predicted gene, 33024	3.10	0.00
ENSMUSG00000025104	Hdgfl3	HDGF like 3	3.10	0.00
ENSMUSG00000037503	Fam168b	family with sequence similarity 168 member B	3.10	0.00
ENSMUSG00000024038	Ndufv3	NADH:ubiquinone oxidoreductase subunit V3	3.11	0.00
ENSMUSG00000054716	Zfp771	zinc finger protein 771	3.11	0.04
ENSMUSG00000051703	Tmem198	transmembrane protein 198	3.13	0.00
ENSMUSG00000044122	Proca1	protein interacting with cyclin A1	3.13	0.00

Essemble ID	Gene Symbol	Gene Description	Fold Change	P Value
ENSMUSG00000020679	Hnf1b	HNF1 homeobox B	3.13	0.00
ENSMUSG00000103789	Gm10129	predicted gene 10129	3.13	0.00
ENSMUSG00000031661	Nkd1	NKD inhibitor of WNT signaling pathway 1	3.13	0.03
ENSMUSG00000024369	Nelfe	negative elongation factor complex member E	3.14	0.00
ENSMUSG00000006642	Tcf23	transcription factor 23	3.15	0.00
ENSMUSG00000034974	Dapk3	death associated protein kinase 3	3.15	0.00
ENSMUSG00000073433	Arhgdig	Rho GDP dissociation inhibitor gamma	3.16	0.00
ENSMUSG00000027677	Ttc14	tetratricopeptide repeat domain 14	3.16	0.00
ENSMUSG00000001034	Mapk7	mitogen-activated protein kinase 7	3.16	0.00
ENSMUSG00000018819	Lsp1	lymphocyte specific protein 1	3.16	0.00
ENSMUSG00000028030	Tbck	TBC1 domain containing kinase	3.16	0.00
ENSMUSG00000024833	Pola2	DNA polymerase alpha 2, accessory subunit	3.16	0.00
ENSMUSG00000099146	0610031O16Rik	RIKEN cDNA 0610031O16 gene	3.17	0.00
ENSMUSG00000026043	Col3a1	collagen type III alpha 1 chain	3.17	0.00
ENSMUSG00000084844	Hoxb3os	homeobox B3 and homeobox B2, opposite strand	3.17	0.00
ENSMUSG00000018548	Trim37	tripartite motif containing 37	3.17	0.00
ENSMUSG00000116308	Gm49482	predicted gene, 49482	3.18	0.01
ENSMUSG00000011267	Zfp296	zinc finger protein 296	3.18	0.00
ENSMUSG00000059323	Tonsl	tonsoku like, DNA repair protein	3.19	0.00
ENSMUSG00000054493	Gm9947	predicted gene 9947	3.19	0.01
ENSMUSG00000032024	Clmp	CXADR like membrane protein	3.19	0.00
ENSMUSG00000016520	Lnx2	ligand of numb-protein X 2	3.19	0.01
ENSMUSG00000097772	5430416N02Rik	RIKEN cDNA 5430416N02 gene	3.20	0.01
ENSMUSG00000074385	Gm10684	predicted gene 10684	3.20	0.00
ENSMUSG00000106888	Gm42848	predicted gene 42848	3.20	0.01
ENSMUSG00000052957	Gas1	growth arrest specific 1	3.20	0.00
ENSMUSG00000029862	Clcn1	chloride voltage-gated channel 1	3.21	0.00
ENSMUSG00000026837	Col5a1	collagen type V alpha 1 chain	3.21	0.00
ENSMUSG00000051650	B3gnt2	UDP-GlcNAc:betaGal beta-1,3-N-acetylglucosaminyltransferase 2	3.21	0.00
ENSMUSG00000031207	Msn	moesin	3.21	0.00
ENSMUSG00000024922	Ovol1	ovo like transcriptional repressor 1	3.22	0.00
ENSMUSG00000108322	5430431A17Rik	RIKEN cDNA 5430431A17 gene	3.22	0.00
ENSMUSG00000091237	Gm17114	predicted gene 17114	3.22	0.00
ENSMUSG00000017210	Med24	mediator complex subunit 24	3.23	0.01
ENSMUSG00000118394	Gm50475	predicted gene, 50475	3.23	0.00
ENSMUSG00000000903	Vpreb3	V-set pre-B cell surrogate light chain 3	3.23	0.00
ENSMUSG00000070495	Ctcfl	CCCTC-binding factor like	3.24	0.00
ENSMUSG00000032383	Ppib	peptidylprolyl isomerase B	3.24	0.00
ENSMUSG00000075589	Gm11536	predicted gene 11536	3.24	0.00
ENSMUSG00000097885	5031434O11Rik	RIKEN cDNA 5031434O11 gene	3.24	0.00
ENSMUSG00000020674	Pxdn	peroxidasin	3.25	0.00
ENSMUSG00000097617	Gm10687	predicted gene 10687	3.25	0.00
ENSMUSG00000086356	Gm13441	predicted gene 13441	3.25	0.00
ENSMUSG00000034674	Tdg	thymine DNA glycosylase	3.25	0.00
ENSMUSG00000116942	Gm49744	predicted gene, 49744	3.25	0.02
ENSMUSG00000001168	Oas1h	2'-5' oligoadenylate synthetase 1H	3.26	0.01

Essemble ID	Gene Symbol	Gene Description	Fold Change	P Value
ENSMUSG00000098620	Gm27209	predicted gene 27209	3.26	0.00
ENSMUSG00000057897	Camk2b	calcium/calmodulin dependent protein kinase II beta	3.26	0.00
ENSMUSG00000086841	2410006H16Rik	RIKEN cDNA 2410006H16 gene	3.26	0.00
ENSMUSG00000062328	Rpl17	ribosomal protein L17	3.26	0.00
ENSMUSG00000044072	Eml6	EMAP like 6	3.26	0.00
ENSMUSG00000026176	Ctdsp1	CTD small phosphatase 1	3.27	0.00
ENSMUSG00000103857	Gm37249	predicted gene, 37249	3.27	0.01
ENSMUSG00000085414	Sspnos	sarcospan, opposite strand	3.27	0.00
ENSMUSG00000104443	4932442E05Rik	RIKEN cDNA 4932442E05 gene	3.28	0.00
ENSMUSG00000020812	Snhg16	small nucleolar RNA host gene 16	3.28	0.00
ENSMUSG00000085095	Gm15635	predicted gene 15635	3.28	0.00
ENSMUSG00000034593	Myo5a	myosin VA	3.28	0.00
ENSMUSG00000057072	Spata45	spermatogenesis associated 45	3.28	0.04
ENSMUSG00000111943	Gm47862	predicted gene, 47862	3.28	0.00
ENSMUSG00000003099	Ppp5c	protein phosphatase 5 catalytic subunit	3.28	0.01
ENSMUSG00000040666	Sh3bgr	SH3-binding domain glutamic acid-rich protein	3.29	0.00
ENSMUSG00000031775	Pllp	plasmolipin	3.29	0.00
ENSMUSG00000087272	Gm15409	predicted gene 15409	3.29	0.00
ENSMUSG00000074227	Spint2	serine peptidase inhibitor, Kunitz type 2	3.29	0.01
ENSMUSG00000120024	Gm53692	predicted gene, 53692	3.29	0.02
ENSMUSG00000079505	Gm11131	predicted gene 11131	3.29	0.00
ENSMUSG00000085315	A430018G15Rik	RIKEN cDNA A430018G15 gene	3.29	0.00
ENSMUSG00000075410	Prcd	photoreceptor disc component	3.30	0.00
ENSMUSG00000105391	Gm43401	predicted gene 43401	3.31	0.00
ENSMUSG00000031626	Sorbs2	sorbin and SH3 domain containing 2	3.31	0.00
ENSMUSG00000120686	Gm6297	predicted gene 6297	3.32	0.00
ENSMUSG00000042599	Kdm7a	lysine demethylase 7A	3.32	0.00
ENSMUSG00000032470	Mras	muscle RAS oncogene homolog	3.32	0.01
ENSMUSG00000095332	Gm9821	predicted gene 9821	3.33	0.00
ENSMUSG00000031825	Crispld2	cysteine rich secretory protein LCCL domain containing 2	3.33	0.00
ENSMUSG00000075585	6330403L08Rik	RIKEN cDNA 6330403L08 gene	3.33	0.00
ENSMUSG00000054517	Trim65	tripartite motif containing 65	3.33	0.00
ENSMUSG00000087480	Gm15910	predicted gene 15910	3.34	0.00
ENSMUSG00000023019	Gpd1	glycerol-3-phosphate dehydrogenase 1	3.34	0.00
ENSMUSG00000024966	Stip1	stress induced phosphoprotein 1	3.34	0.00
ENSMUSG00000100865	Gm9320	ribosomal protein S24 pseudogene	3.35	0.00
ENSMUSG00000087381	Gm16008	predicted gene 16008	3.36	0.00
ENSMUSG00000089669	Tnfsf13	TNF superfamily member 13	3.36	0.00
ENSMUSG00000097083	D930019O06Rik	RIKEN cDNA D930019O06	3.36	0.02
ENSMUSG00000091908	Gm17231	predicted gene 17231	3.36	0.00
ENSMUSG00000022665	Ccdc80	coiled-coil domain containing 80	3.37	0.00
ENSMUSG00000022757	Tfg	trafficking from ER to golgi regulator	3.37	0.00
ENSMUSG00000061451	Tmem151a	transmembrane protein 151A	3.37	0.00
ENSMUSG00000034892	Rps29	ribosomal protein S29	3.37	0.00
ENSMUSG00000062590	Armc9	armadillo repeat containing 9	3.37	0.00
ENSMUSG00000120216	Gm57014	predicted gene, 57014	3.37	0.00

Essemble ID	Gene Symbol	Gene Description	Fold Change	P Value
ENSMUSG00000048779	P2ry6	pyrimidinergic receptor P2Y6	3.37	0.00
ENSMUSG00000031355	Arhgap6	Rho GTPase activating protein 6	3.37	0.00
ENSMUSG00000079144	A130010J15Rik	chromosome 1 open reading frame 74	3.38	0.00
ENSMUSG00000047407	Tgif1	TGFB induced factor homeobox 1	3.38	0.00
ENSMUSG00000081223	Gm12247	predicted gene 12247	3.39	0.03
ENSMUSG00000036894	Rap2b	RAP2B, member of RAS oncogene family	3.40	0.00
ENSMUSG00000027438	Napb	NSF attachment protein beta	3.40	0.00
ENSMUSG00000078972	Gm15557	predicted gene 15557	3.41	0.00
ENSMUSG00000073557	Ppp1r12b	protein phosphatase 1, regulatory subunit 12B	3.41	0.02
ENSMUSG00000022895	Ets2	ETS proto-oncogene 2, transcription factor	3.41	0.01
ENSMUSG00000091745	Gm17098	predicted gene 17098	3.41	0.00
ENSMUSG00000086527	Gm15856	predicted gene 15856	3.42	0.04
ENSMUSG00000024620	Pdgfrb	platelet derived growth factor receptor beta	3.42	0.01
ENSMUSG00000066902	Rps23-ps2	ribosomal protein S23, pseudogene 2	3.43	0.02
ENSMUSG00000120673	Gm56532	predicted gene, 56532	3.43	0.00
ENSMUSG00000020474	Polm	DNA polymerase mu	3.43	0.00
ENSMUSG00000060126	Tpt1	tumor protein, translationally-controlled 1	3.43	0.00
ENSMUSG00000075415	Fnbp1	formin binding protein 1	3.44	0.00
ENSMUSG00000097354	2310001H17Rik	RIKEN cDNA 2310001H17 gene	3.44	0.00
ENSMUSG00000020929	Eftud2	elongation factor Tu GTP binding domain containing 2	3.45	0.00
ENSMUSG00000117872	A530088E08Rik	RIKEN cDNA A530088E08 gene	3.45	0.00
ENSMUSG00000023092	Fhl1	four and a half LIM domains 1	3.45	0.01
ENSMUSG00000043051	Disc1	DISC1 scaffold protein	3.45	0.00
ENSMUSG00000097003	D930007P13Rik	Riken cDNA D930007P13 gene	3.45	0.00
ENSMUSG00000023104	Rfc2	replication factor C subunit 2	3.45	0.00
ENSMUSG00000074211	Sdhaf1	succinate dehydrogenase complex assembly factor 1	3.45	0.00
ENSMUSG00000089715	Cbx6	chromobox 6	3.45	0.01
ENSMUSG00000041147	Brca2	BRCA2 DNA repair associated	3.46	0.00
ENSMUSG00000102605	Gm37264	predicted gene, 37264	3.46	0.00
ENSMUSG00000027593	Raly	RALY heterogeneous nuclear ribonucleoprotein	3.46	0.00
ENSMUSG00000097419	Gm26666	predicted gene, 26666	3.47	0.00
ENSMUSG00000036136	Fam110c	family with sequence similarity 110 member C	3.47	0.00
ENSMUSG00000032135	Mcam	melanoma cell adhesion molecule	3.48	0.00
ENSMUSG00000028763	Hspg2	heparan sulfate proteoglycan 2	3.48	0.00
ENSMUSG00000041560	Nop53	NOP53 ribosome biogenesis factor	3.48	0.00
ENSMUSG00000042289	Hsd3b7	hydroxy-delta-5-steroid dehydrogenase, 3 beta- and steroid delta-isomerase 7	3.48	0.01
ENSMUSG00000120890	Gm56665	predicted gene, 56665	3.48	0.01
ENSMUSG00000087389	Gm15592	predicted gene 15592	3.48	0.00
ENSMUSG00000029556	Hnf1a	HNF1 homeobox A	3.49	0.01
ENSMUSG00000020827	Mink1	misshapen like kinase 1	3.50	0.00
ENSMUSG00000028334	Nans	N-acetylneuraminate synthase	3.50	0.00
ENSMUSG00000090152	Gm16285	predicted gene 16285	3.50	0.00
ENSMUSG00000021620	Acot12	acyl-CoA thioesterase 12	3.50	0.01
ENSMUSG00000035342	Lzts2	leucine zipper tumor suppressor 2	3.50	0.00
ENSMUSG00000113404	Gm48622	predicted gene, 48622	3.50	0.00
ENSMUSG00000073529	F830208F22Rik	RIKEN cDNA F830208F22 gene	3.50	0.00

Essemble ID	Gene Symbol	Gene Description	Fold Change	P Value
ENSMUSG00000107317	Gm19719	predicted gene, 19719	3.51	0.00
ENSMUSG00000027651	Rprd1b	regulation of nuclear pre-mRNA domain containing 1B	3.51	0.00
ENSMUSG00000045775	Slc16a5	solute carrier family 16 member 5	3.51	0.00
ENSMUSG00000039062	Anpep	alanyl aminopeptidase, membrane	3.52	0.01
ENSMUSG00000097616	1110019D14Rik	RIKEN cDNA 1110019D14 gene	3.52	0.00
ENSMUSG00000090558	Gm17202	predicted gene 17202	3.52	0.00
ENSMUSG00000086098	Gm14291	predicted gene 14291	3.53	0.00
ENSMUSG00000019846	Lama4	laminin subunit alpha 4	3.53	0.00
ENSMUSG00000034758	Tle6	TLE family member 6, subcortical maternal complex member	3.54	0.00
ENSMUSG00000008206	Cers4	ceramide synthase 4	3.54	0.00
ENSMUSG00000056445	Hoxaas2	Hoxa cluster antisense RNA 2	3.55	0.00
ENSMUSG00000053553	3110082I17Rik	chromosome 7 open reading frame 50	3.55	0.00
ENSMUSG00000018750	Zbtb4	zinc finger and BTB domain containing 4	3.55	0.00
ENSMUSG00000031543	Ank1	ankyrin 1	3.56	0.00
ENSMUSG00000117231	Gm41609	predicted gene, 41609	3.56	0.00
ENSMUSG00000092371	Gm20511	predicted gene 20511	3.56	0.00
ENSMUSG00000086604	Gm15510	predicted gene 15510	3.56	0.00
ENSMUSG00000051652	Lrrc3	leucine rich repeat containing 3	3.57	0.04
ENSMUSG00000041189	Chrnb1	cholinergic receptor nicotinic beta 1 subunit	3.57	0.00
ENSMUSG00000036281	Snapc4	small nuclear RNA activating complex polypeptide 4	3.58	0.01
ENSMUSG00000025081	Tdrd1	tudor domain containing 1	3.58	0.03
ENSMUSG00000026421	Csrp1	cysteine and glycine rich protein 1	3.58	0.00
ENSMUSG00000025869	Nop16	NOP16 nucleolar protein	3.58	0.01
ENSMUSG00000040488	Ltbp4	latent transforming growth factor beta binding protein 4	3.59	0.00
ENSMUSG00000028967	Errfi1	ERBB receptor feedback inhibitor 1	3.59	0.00
ENSMUSG00000036620	Mgat4b	alpha-1,3-mannosyl-glycoprotein 4-beta-N-acetylglucosaminyltransferase B	3.60	0.00
ENSMUSG00000086918	4930429F24Rik	RIKEN cDNA 4930429F24 gene	3.60	0.00
ENSMUSG00000100280	Gm28417	predicted gene 28417	3.60	0.00
ENSMUSG00000038024	Dennd4c	DENN domain containing 4C	3.61	0.00
ENSMUSG00000025902	Sox17	SRY-box transcription factor 17	3.61	0.00
ENSMUSG00000090174	Gm10612	predicted gene 10612	3.61	0.00
ENSMUSG00000104238	Gm37587	predicted gene, 37587	3.62	0.00
ENSMUSG00000031390	Avpr2	arginine vasopressin receptor 2	3.62	0.00
ENSMUSG00000027857	Tshb	thyroid stimulating hormone subunit beta	3.62	0.00
ENSMUSG00000090394	4930523C07Rik	KIAA0040	3.62	0.00
ENSMUSG00000021464	Ror2	receptor tyrosine kinase like orphan receptor 2	3.63	0.00
ENSMUSG00000097328	Tnfsf12	TNF superfamily member 12	3.63	0.00
ENSMUSG00000061207	Stk19	serine/threonine kinase 19	3.63	0.02
ENSMUSG00000107002	Ncbp2as2	NCBP2 antisense 2 (head to head)	3.63	0.00
ENSMUSG00000027577	Chrna4	cholinergic receptor nicotinic alpha 4 subunit	3.63	0.00
ENSMUSG00000078706	Gm53	predicted gene 53	3.63	0.00
ENSMUSG00000026463	Atp2b4	ATPase plasma membrane Ca2+ transporting 4	3.64	0.00
ENSMUSG00000007805	Twist2	twist family bHLH transcription factor 2	3.64	0.01
ENSMUSG00000007038	Neu1	neuraminidase 1	3.64	0.00
ENSMUSG00000024049	Myom1	myomesin 1	3.65	0.00
ENSMUSG00000040774	Cept1	choline/ethanolamine phosphotransferase 1	3.65	0.00

Essemble ID	Gene Symbol	Gene Description	Fold Change	P Value
ENSMUSG00000086032	Gm15929	predicted gene 15929	3.65	0.00
ENSMUSG00000028463	Car9	carbonic anhydrase 9	3.66	0.00
ENSMUSG00000086451	4933431K23Rik	RIKEN cDNA 4933431K23 gene	3.66	0.00
ENSMUSG00000048126	Col6a3	collagen type VI alpha 3 chain	3.66	0.00
ENSMUSG00000094035	Ldlrad2	low density lipoprotein receptor class A domain containing 2	3.66	0.00
ENSMUSG00000046806	Cyren	cell cycle regulator of NHEJ	3.67	0.00
ENSMUSG00000031570	Plpp5	phospholipid phosphatase 5	3.67	0.00
ENSMUSG00000031375	Bgn	biglycan	3.67	0.01
ENSMUSG00000089759	3632454L22Rik	RIKEN cDNA 3632454L22 gene	3.68	0.00
ENSMUSG00000102877	Gm38314	predicted gene, 38314	3.68	0.00
ENSMUSG00000026833	Olfm1	olfactomedin 1	3.68	0.00
ENSMUSG00000116840	Gm21691	predicted gene, 21691	3.68	0.00
ENSMUSG00000092609	Gm20481	predicted gene 20481	3.70	0.00
ENSMUSG00000033685	Ucp2	uncoupling protein 2	3.70	0.01
ENSMUSG00000026031	Cflar	CASP8 and FADD like apoptosis regulator	3.70	0.00
ENSMUSG00000086835	Gm11775	predicted gene 11775	3.70	0.00
ENSMUSG00000022394	L3mbtl2	L3MBTL histone methyl-lysine binding protein 2	3.71	0.00
ENSMUSG00000030870	Ubfd1	ubiquitin family domain containing 1	3.71	0.00
ENSMUSG00000034254	Agpat1	1-acylglycerol-3-phosphate O-acyltransferase 1	3.71	0.00
ENSMUSG00000090307	1700071M16Rik	RIKEN cDNA 1700071M16 gene	3.71	0.00
ENSMUSG00000103219	Gm37787	predicted gene, 37787	3.72	0.00
ENSMUSG00000085957	Syna	syncytin a	3.72	0.00
ENSMUSG00000024664	Fads3	fatty acid desaturase 3	3.74	0.00
ENSMUSG00000020864	Ankrd40	ankyrin repeat domain 40	3.74	0.00
ENSMUSG00000045100	Slc25a26	solute carrier family 25 member 26	3.74	0.00
ENSMUSG00000032875	Arhgef17	Rho guanine nucleotide exchange factor 17	3.75	0.00
ENSMUSG00000024236	Svil	supervillin	3.75	0.00
ENSMUSG00000092283	Gm20412	predicted gene 20412	3.75	0.00
ENSMUSG00000021666	Gfm2	GTP dependent ribosome recycling factor mitochondrial 2	3.76	0.00
ENSMUSG00000050931	Sgms2	sphingomyelin synthase 2	3.77	0.00
ENSMUSG00000111888	Gm46212	predicted gene, 46212	3.77	0.00
ENSMUSG00000116665	E130310I04Rik	RIKEN cDNA E130310I04 gene	3.78	0.00
ENSMUSG00000031600	Vps37a	VPS37A subunit of ESCRT-I	3.78	0.00
ENSMUSG00000097836	Gm26903	predicted gene, 26903	3.78	0.00
ENSMUSG00000028906	Epb41	erythrocyte membrane protein band 4.1	3.78	0.00
ENSMUSG00000091575	2010016I18Rik	RIKEN cDNA 2010016I18 gene	3.78	0.00
ENSMUSG00000018899	Irf1	interferon regulatory factor 1	3.78	0.00
ENSMUSG00000087515	Gm13554	predicted gene 13554	3.78	0.00
ENSMUSG00000116241	Gm49458	predicted gene, 49458	3.78	0.01
ENSMUSG00000040152	Thbs1	thrombospondin 1	3.79	0.00
ENSMUSG00000030748	Il4ra	interleukin 4 receptor	3.79	0.00
ENSMUSG00000106155	Gm43495	predicted gene 43495	3.80	0.00
ENSMUSG00000023169	Slc38a1	solute carrier family 38 member 1	3.81	0.00
ENSMUSG00000085071	Gm14066	predicted gene 14066	3.83	0.00
ENSMUSG00000015968	Cacna1d	calcium voltage-gated channel subunit alpha1 D	3.83	0.00
ENSMUSG00000022678	Nde1	nudE neurodevelopment protein 1	3.83	0.00

Essemble ID	Gene Symbol	Gene Description	Fold Change	P Value
ENSMUSG00000112652	4921516A02Rik	RIKEN cDNA 4921516A02 gene	3.83	0.00
ENSMUSG00000101859	Gm29233	predicted gene 29233	3.83	0.00
ENSMUSG00000023967	Mrps18a	mitochondrial ribosomal protein S18A	3.84	0.00
ENSMUSG00000042613	Pbxip1	PBX homeobox interacting protein 1	3.84	0.00
ENSMUSG00002075603	Gm55583	predicted gene, 55583	3.84	0.00
ENSMUSG00000116162	Gm36329	predicted gene, 36329	3.84	0.01
ENSMUSG00000023039	Krt7	keratin 7	3.84	0.01
ENSMUSG00000078588	Ccdc24	coiled-coil domain containing 24	3.86	0.00
ENSMUSG00000033200	Tpsg1	tryptase gamma 1	3.86	0.00
ENSMUSG00000019929	Dcn	decorin	3.87	0.00
ENSMUSG00000030726	Pold3	DNA polymerase delta 3, accessory subunit	3.87	0.01
ENSMUSG00000030672	Mylpf	myosin light chain 11	3.88	0.00
ENSMUSG00000118295	Gm8437	predicted gene 8437	3.88	0.01
ENSMUSG00000060098	Prmt7	protein arginine methyltransferase 7	3.88	0.00
ENSMUSG00000024112	Cacna1h	calcium voltage-gated channel subunit alpha1 H	3.88	0.00
ENSMUSG00000086937	Gm15063	predicted gene 15063	3.89	0.00
ENSMUSG00000031891	Hsd11b2	hydroxysteroid 11-beta dehydrogenase 2	3.89	0.03
ENSMUSG00000033763	Mtss2	MTSS I-BAR domain containing 2	3.89	0.00
ENSMUSG00000030782	Tgfb1i1	transforming growth factor beta 1 induced transcript 1	3.89	0.01
ENSMUSG00000029600	Rita1	RBPJ interacting and tubulin associated 1	3.91	0.00
ENSMUSG00000112765	9430078K24Rik	RIKEN cDNA 9430078K24 gene	3.92	0.00
ENSMUSG00000108232	Gm43916	predicted gene, 43916	3.92	0.00
ENSMUSG00000015750	Aph1a	aph-1 homolog A, gamma-secretase subunit	3.93	0.00
ENSMUSG00000105745	Gm42521	predicted gene 42521	3.93	0.04
ENSMUSG00000097160	A630072L19Rik	RIKEN cDNA A630072L19 gene	3.93	0.00
ENSMUSG00000051378	Kif18b	kinesin family member 18B	3.93	0.01
ENSMUSG00002075428	Gm54609	predicted gene, 54609	3.94	0.05
ENSMUSG00000085227	6330418K02Rik	RIKEN cDNA 6330418K02 gene	3.95	0.00
ENSMUSG00000028364	Tnc	tenascin C	3.95	0.03
ENSMUSG00000117179	Gm49907	predicted gene, 49907	3.96	0.00
ENSMUSG00000045231	BC106179	cDNA sequence BC106179	3.96	0.00
ENSMUSG00000029096	Htra3	HtrA serine peptidase 3	3.97	0.02
ENSMUSG00000050234	Gja4	gap junction protein alpha 4	3.97	0.00
ENSMUSG00000034786	Gpsm3	G protein signaling modulator 3	3.97	0.00
ENSMUSG00000117964	Gm36043	predicted gene, 36043	3.97	0.00
ENSMUSG00000037443	Cep85	centrosomal protein 85	3.98	0.01
ENSMUSG00000032589	Bsn	bassoon presynaptic cytomatrix protein	3.98	0.00
ENSMUSG00000107736	Gm44148	predicted gene, 44148	3.98	0.00
ENSMUSG00000056648	Hoxb8	homeobox B8	3.98	0.01
ENSMUSG00000060950	Trmt61a	tRNA methyltransferase 61A	3.98	0.00
ENSMUSG00000060380	C030014I23Rik	RIKEN cDNA C030014I23 gene	3.99	0.00
ENSMUSG00000056468	5730596B20Rik	RIKEN cDNA 5730596B20 gene	3.99	0.00
ENSMUSG00000041881	Ndufa7	NADH:ubiquinone oxidoreductase subunit A7	4.00	0.01
ENSMUSG00000034543	Morc2a	MORC family CW-type zinc finger 2	4.01	0.01
ENSMUSG00000051331	Cacna1c	calcium voltage-gated channel subunit alpha1 C	4.03	0.00
ENSMUSG00000041571	Selenow	selenoprotein W	4.03	0.00

Essemble ID	Gene Symbol	Gene Description	Fold Change	P Value
ENSMUSG00000085888	Gm12224	predicted gene 12224	4.03	0.00
ENSMUSG00000109866	Gm45714	predicted gene 45714	4.03	0.00
ENSMUSG00000025492	Ifitm3	interferon induced transmembrane protein 3	4.04	0.00
ENSMUSG00000054640	Slc8a1	solute carrier family 8 member A1	4.05	0.00
ENSMUSG00000026830	Ermn	ermin	4.05	0.01
ENSMUSG00000058470	Gm8369	predicted gene 8369	4.05	0.00
ENSMUSG00000098871	Mir6381	microRNA 6381	4.06	0.00
ENSMUSG00000018920	Cxcl16	C-X-C motif chemokine ligand 16	4.06	0.00
ENSMUSG00000055216	9430025C20Rik	RIKEN cDNA 9430025C20 gene	4.07	0.00
ENSMUSG00000121087	Gm56902	predicted gene, 56902	4.08	0.00
ENSMUSG00000022664	Slc35a5	solute carrier family 35 member A5	4.08	0.00
ENSMUSG00000031451	Gas6	growth arrest specific 6	4.08	0.00
ENSMUSG00000058558	Rpl5	ribosomal protein L5	4.08	0.00
ENSMUSG00000038520	Tbc1d17	TBC1 domain family member 17	4.08	0.00
ENSMUSG00000108171	Gm43915	predicted gene, 43915	4.09	0.00
ENSMUSG00000021127	Zfp36l1	ZFP36 ring finger protein like 1	4.09	0.00
ENSMUSG00000019467	Arhgef25	Rho guanine nucleotide exchange factor 25	4.10	0.00
ENSMUSG00000013766	Ly6g6e	lymphocyte antigen 6 family member G6E	4.10	0.00
ENSMUSG00000032366	Tpm1	tropomyosin 1, alpha	4.10	0.00
ENSMUSG00000052273	Dnah3	dynein axonemal heavy chain 3	4.10	0.00
ENSMUSG00000107428	Gm44154	predicted gene, 44154	4.11	0.00
ENSMUSG00000105403	Gm43618	predicted gene 43618	4.11	0.00
ENSMUSG00000031636	Pdlim3	PDZ and LIM domain 3	4.11	0.00
ENSMUSG00000025380	Fscn2	fascin actin-bundling protein 2, retinal	4.12	0.00
ENSMUSG00000111737	Gm47248	predicted gene, 47248	4.12	0.03
ENSMUSG00000110831	Gm48159	predicted gene, 48159	4.13	0.00
ENSMUSG00000026427	Eif2d	eukaryotic translation initiation factor 2D	4.14	0.00
ENSMUSG00000020810	Cygb	cytoglobin	4.14	0.00
ENSMUSG00000120880	1700029K24Rik	RIKEN cDNA 1700029K24 gene	4.14	0.00
ENSMUSG00000102717	Gm37759	predicted gene, 37759	4.16	0.00
ENSMUSG00000112429	Gm47093	predicted gene, 47093	4.16	0.00
ENSMUSG00000120280	Gm56713	predicted gene, 56713	4.16	0.00
ENSMUSG00000086327	Slfn5os	schlafen 5, opposite strand	4.18	0.00
ENSMUSG00000075600	Zc3h3	zinc finger CCCH-type containing 3	4.19	0.02
ENSMUSG00000097315	Gm26568	predicted gene, 26568	4.19	0.00
ENSMUSG00000027339	Rassf2	Ras association domain family member 2	4.20	0.00
ENSMUSG00000003604	Aven	apoptosis and caspase activation inhibitor	4.20	0.00
ENSMUSG00000089647	Gm2245	predicted gene 2245	4.21	0.00
ENSMUSG00000018378	2210416O15Rik	RIKEN cDNA 2210416O15 gene	4.21	0.00
ENSMUSG00000097194	9330175E14Rik	RIKEN cDNA 9330175E14 gene	4.21	0.00
ENSMUSG00000104354	Gm37126	predicted gene, 37126	4.21	0.00
ENSMUSG00000040462	Os9	OS9 endoplasmic reticulum lectin	4.22	0.00
ENSMUSG00000087030	Gm16143	predicted gene 16143	4.22	0.02
ENSMUSG00000065420	Mir142b	microRNA 142	4.24	0.00
ENSMUSG00000055629	B4galnt4	beta-1,4-N-acetyl-galactosaminyltransferase 4	4.25	0.03
ENSMUSG00000115902	D730005E14Rik	RIKEN cDNA D730005E14 gene	4.25	0.00

Essemble ID	Gene Symbol	Gene Description	Fold Change	P Value
ENSMUSG00000031328	Flna	filamin A	4.25	0.00
ENSMUSG00000057914	Cacnb2	calcium voltage-gated channel auxiliary subunit beta 2	4.27	0.00
ENSMUSG00000110498	A630001O12Rik	RIKEN cDNA A630001O12 gene	4.27	0.00
ENSMUSG00000053749	Gm9920	predicted gene 9920	4.28	0.00
ENSMUSG00000029661	Col1a2	collagen type I alpha 2 chain	4.28	0.00
ENSMUSG00000004105	Angptl2	angiopoietin like 2	4.28	0.00
ENSMUSG00000018774	Cd68	CD68 molecule	4.28	0.00
ENSMUSG00000018752	Tnfsfm13	TNFSF12-TNFSF13 readthrough	4.28	0.00
ENSMUSG00000021185	Dglucy	D-glutamate cyclase	4.28	0.00
ENSMUSG00000018143	Mafk	MAF bZIP transcription factor K	4.28	0.00
ENSMUSG00000017969	Ptgis	prostaglandin I2 synthase	4.30	0.00
ENSMUSG00000008028	1700008O03Rik	chromosome 19 open reading frame 81	4.31	0.00
ENSMUSG00000020377	Ltc4s	leukotriene C4 synthase	4.31	0.00
ENSMUSG00000106015	Gm42980	predicted gene 42980	4.31	0.02
ENSMUSG00000097222	Gata6os	GATA binding protein 6, opposite strand	4.31	0.00
ENSMUSG00000120685	Gm41846	predicted gene, 41846	4.31	0.00
ENSMUSG00000058441	Panx2	pannexin 2	4.32	0.00
ENSMUSG00000048644	Ctxn1	cortexin 1	4.33	0.01
ENSMUSG00000094962	Gm21954	predicted gene, 21954	4.33	0.00
ENSMUSG00000121089	Gm57052	predicted gene, 57052	4.33	0.00
ENSMUSG00000020932	Gfap	glial fibrillary acidic protein	4.34	0.00
ENSMUSG00000018845	Unc45b	unc-45 myosin chaperone B	4.34	0.00
ENSMUSG00000031995	St14	ST14 transmembrane serine protease matriptase	4.34	0.02
ENSMUSG00000026883	Dab2ip	DAB2 interacting protein	4.35	0.00
ENSMUSG00000041757	Plekha6	pleckstrin homology domain containing, family A member 6	4.35	0.00
ENSMUSG00000030030	1700003E16Rik	chromosome 2 open reading frame 81	4.35	0.00
ENSMUSG00000044550	Tceal3	transcription elongation factor A like 6	4.36	0.00
ENSMUSG00000038319	Kcnh2	potassium voltage-gated channel subfamily H member 2	4.36	0.01
ENSMUSG00000032413	Rasa2	RAS p21 protein activator 2	4.37	0.00
ENSMUSG00000000673	Haao	3-hydroxyanthranilate 3,4-dioxygenase	4.37	0.00
ENSMUSG00000041731	Pgm5	phosphoglucomutase 5	4.37	0.00
ENSMUSG00000056290	Ms4a4b	membrane-spanning 4-domains, subfamily A, member 4C	4.38	0.00
ENSMUSG00000022419	Deptor	DEP domain containing MTOR interacting protein	4.38	0.00
ENSMUSG00000097923	Gm26577	predicted gene, 26577	4.38	0.00
ENSMUSG00000030218	Mgp	matrix Gla protein	4.39	0.00
ENSMUSG00000046027	Stard5	StAR related lipid transfer domain containing 5	4.39	0.00
ENSMUSG00000060586	H2-Eb1	major histocompatibility complex, class II, DR beta 5	4.39	0.00
ENSMUSG00000101223	4930568A12Rik	RIKEN cDNA 4930568A12 gene	4.40	0.00
ENSMUSG00000085604	Dhx58os	DExH-box helicase 58, opposite strand	4.40	0.00
ENSMUSG00000092482	Gm20531	predicted gene 20531	4.41	0.00
ENSMUSG00000020241	Col6a2	collagen type VI alpha 2 chain	4.42	0.00
ENSMUSG00000005533	Igf1r	insulin like growth factor 1 receptor	4.42	0.00
ENSMUSG00000033966	Cdkl4	cyclin dependent kinase like 4	4.43	0.01
ENSMUSG00000052013	Btla	B and T lymphocyte associated	4.43	0.00
ENSMUSG00000110702	Gm45767	predicted gene 45767	4.44	0.00
ENSMUSG00000120721	Gm56792	predicted gene, 56792	4.45	0.00

Essemble ID	Gene Symbol	Gene Description	Fold Change	P Value
ENSMUSG00000019823	Mical1	microtubule associated monooxygenase, calponin and LIM domain containing 1	4.45	0.00
ENSMUSG00000092071	A230065N10Rik	RIKEN cDNA A230065N10 gene	4.46	0.00
ENSMUSG00000040690	Col16a1	collagen type XVI alpha 1 chain	4.47	0.00
ENSMUSG00000100121	1700025N23Rik	RIKEN cDNA 1700025N23 gene	4.48	0.01
ENSMUSG00000054675	Tmem119	transmembrane protein 119	4.51	0.00
ENSMUSG00000085491	4930527E20Rik	RIKEN cDNA 4930527E20 gene	4.52	0.01
ENSMUSG00000028871	Rspo1	R-spondin 1	4.52	0.00
ENSMUSG00000031703	Itfg1	integrin alpha FG-GAP repeat containing 1	4.53	0.00
ENSMUSG00000041840	Haus1	HAUS augmin like complex subunit 1	4.53	0.00
ENSMUSG00000024736	Tmem132a	transmembrane protein 132A	4.54	0.00
ENSMUSG00000020829	Slc46a1	solute carrier family 46 member 1	4.54	0.00
ENSMUSG00000001508	Sgca	sarcoglycan alpha	4.54	0.00
ENSMUSG00000000194	Gpr107	G protein-coupled receptor 107	4.58	0.04
ENSMUSG00000026365	Cfh	complement factor H	4.59	0.00
ENSMUSG00000039098	Gm9767	predicted gene 9767	4.61	0.00
ENSMUSG00000021273	Fdft1	farnesyl-diphosphate farnesyltransferase 1	4.63	0.00
ENSMUSG00000039018	Mtg1	mitochondrial ribosome associated GTPase 1	4.63	0.00
ENSMUSG00000107567	4930480K02Rik	RIKEN cDNA 4930480K02 gene	4.63	0.00
ENSMUSG00000090208	Gm15851	predicted gene 15851	4.63	0.00
ENSMUSG00000015461	Atf6b	activating transcription factor 6 beta	4.63	0.00
ENSMUSG00000085696	Hoxaas3	Hoxa cluster antisense RNA 3	4.63	0.00
ENSMUSG00000020439	Smtn	smoothelin	4.63	0.00
ENSMUSG00000037058	Paip2	poly(A) binding protein interacting protein 2	4.64	0.00
ENSMUSG00000025477	Inpp5a	inositol polyphosphate-5-phosphatase A	4.65	0.01
ENSMUSG00000022836	Mylk	myosin light chain kinase	4.66	0.00
ENSMUSG00000005611	Irag1	inositol 1,4,5-triphosphate receptor associated 1	4.66	0.00
ENSMUSG00000057236	Rbbp4	RB binding protein 4, chromatin remodeling factor	4.68	0.00
ENSMUSG00000038178	Slc43a2	solute carrier family 43 member 2	4.68	0.00
ENSMUSG00000054263	Lifr	LIF receptor subunit alpha	4.69	0.00
ENSMUSG00000020381	Mrnip	MRN complex interacting protein	4.71	0.00
ENSMUSG00000112766	Gm40645	predicted gene, 40645	4.71	0.00
ENSMUSG00000119994	A430046D13Rik	Riken cDNA A430046D13 gene	4.71	0.00
ENSMUSG00000108854	D830036C21Rik	RIKEN cDNA D830036C21 gene	4.71	0.00
ENSMUSG00000086814	Gm15677	predicted gene 15677	4.71	0.00
ENSMUSG00000097779	4833407H14Rik	RIKEN cDNA 4833407H14 gene	4.73	0.03
ENSMUSG00000001155	Ftcd	formimidoyltransferase cyclodeaminase	4.73	0.00
ENSMUSG00000087431	Gm16054	predicted gene 16054	4.74	0.00
ENSMUSG00000073415	Gm10501	predicted gene 10501	4.75	0.00
ENSMUSG00000086555	Gm13446	predicted gene 13446	4.75	0.00
ENSMUSG00000060212	Pcnx2	pecanex 2	4.75	0.00
ENSMUSG00000016942	Tmprss6	transmembrane serine protease 6	4.76	0.00
ENSMUSG00000099568	Gm28513	predicted gene 28513	4.77	0.00
ENSMUSG00000027447	Cst3	cystatin C	4.77	0.00
ENSMUSG00000052160	Pld4	phospholipase D family member 4	4.78	0.01
ENSMUSG00000098284	A330093E20Rik	RIKEN cDNA A330093E20 gene	4.78	0.00
ENSMUSG00000051367	Six1	SIX homeobox 1	4.79	0.01

Essemble ID	Gene Symbol	Gene Description	Fold Change	P Value
ENSMUSG00000078923	Ube2v1	ubiquitin conjugating enzyme E2 V1	4.79	0.00
ENSMUSG00000047090	Tmem198b	transmembrane protein 198b	4.79	0.00
ENSMUSG00000046711	Hmga1	high mobility group AT-hook 1	4.82	0.00
ENSMUSG00000040688	Tbl3	transducin beta like 3	4.83	0.00
ENSMUSG00000020027	Socs2	suppressor of cytokine signaling 2	4.83	0.00
ENSMUSG00000086866	4930512H18Rik	RIKEN cDNA 4930512H18 gene	4.84	0.00
ENSMUSG00000038143	Stox2	storkhead box 2	4.84	0.00
ENSMUSG00000038738	Shank1	SH3 and multiple ankyrin repeat domains 1	4.87	0.00
ENSMUSG00000070574	2310016G11Rik	RIKEN cDNA 2310016G11 gene	4.88	0.02
ENSMUSG00000027860	Vangl1	VANGL planar cell polarity protein 1	4.89	0.00
ENSMUSG00000049097	Ankrd34a	ankyrin repeat domain 34A	4.91	0.00
ENSMUSG00000051236	Msrb3	methionine sulfoxide reductase B3	4.91	0.00
ENSMUSG00000053214	Gm9899	predicted gene 9899	4.92	0.00
ENSMUSG00000082536	Gm13456	eukaryotic translation elongation factor 1 alpha 1 pseudogene	4.92	0.01
ENSMUSG00000075525	Gm10849	predicted gene 10849	4.93	0.00
ENSMUSG00000026737	Pip4k2a	phosphatidylinositol-5-phosphate 4-kinase type 2 alpha	4.94	0.00
ENSMUSG00000113079	Gm48709	predicted gene, 48709	4.94	0.00
ENSMUSG00000023046	Igfbp6	insulin like growth factor binding protein 6	4.95	0.00
ENSMUSG00000031586	Rbpms	RNA binding protein, mRNA processing factor	4.95	0.00
ENSMUSG00000022108	Itm2b	integral membrane protein 2B	4.96	0.00
ENSMUSG00000020902	Ntn1	netrin 1	4.96	0.00
ENSMUSG00000087633	LOC118568483	uncharacterized LOC118568483	4.97	0.00
ENSMUSG00000036918	Ttc7	tetratricopeptide repeat domain 7A	4.97	0.00
ENSMUSG00000030708	Dnajb13	DnaJ heat shock protein family (Hsp40) member B13	4.98	0.00
ENSMUSG00000049775	Tmsb4x	thymosin, beta 4, X chromosome	4.99	0.00
ENSMUSG00000059430	Actg2	actin gamma 2, smooth muscle	5.01	0.00
ENSMUSG00000015476	Prrt1	proline-rich transmembrane protein 1	5.02	0.00
ENSMUSG00000025034	Trim8	tripartite motif containing 8	5.04	0.00
ENSMUSG00000120165	Gm57379	predicted gene, 57379	5.04	0.00
ENSMUSG00000090246	Gm17017	predicted gene 17017	5.05	0.00
ENSMUSG00000005836	Gata6	GATA binding protein 6	5.07	0.00
ENSMUSG00000048572	Tmem252	transmembrane protein 252	5.08	0.00
ENSMUSG00000028457	Atp8b5	ATPase, class I, type 8B, member 5	5.08	0.00
ENSMUSG00000017764	Zswim1	zinc finger SWIM-type containing 1	5.10	0.00
ENSMUSG00000004931	Apba3	amyloid beta precursor protein binding family A member 3	5.10	0.00
ENSMUSG00000021903	Galnt15	polypeptide N-acetylgalactosaminyltransferase 15	5.10	0.00
ENSMUSG00000090009	Gm16282	predicted gene 16282	5.11	0.00
ENSMUSG00000029029	Wrap73	WD repeat containing, antisense to TP73	5.12	0.00
ENSMUSG00000009585	Apobec3	apolipoprotein B mRNA editing enzyme catalytic subunit 3B	5.12	0.00
ENSMUSG00000087626	Gm15050	predicted gene 15050	5.12	0.00
ENSMUSG00000096107	Gm16505	predicted gene 16505	5.13	0.00
ENSMUSG00000104283	Gm37459	predicted gene, 37459	5.14	0.00
ENSMUSG00000036452	Arhgap26	Rho GTPase activating protein 26	5.14	0.00
ENSMUSG00000033082	Clec1a	C-type lectin domain family 1 member A	5.15	0.01
ENSMUSG00000033318	Gstt2	glutathione S-transferase theta 2B	5.15	0.00
ENSMUSG00000024295	Cyp4f41-ps	cytochrome P450, family 4, subfamily f, polypeptide 41 pseudogene	5.16	0.00

Essemble ID	Gene Symbol	Gene Description	Fold Change	P Value
ENSMUSG00000068263	Efcc1	EF-hand and coiled-coil domain containing 1	5.16	0.00
ENSMUSG00000000530	Acvrl1	activin A receptor like type 1	5.17	0.00
ENSMUSG00000045404	Kcnk13	potassium two pore domain channel subfamily K member 13	5.17	0.00
ENSMUSG00000040586	Ofd1	OFD1 centriole and centriolar satellite protein	5.19	0.00
ENSMUSG00000015971	Actr8	actin related protein 8	5.20	0.00
ENSMUSG00000001270	Ckb	creatine kinase B	5.21	0.00
ENSMUSG00000104653	Gm43109	predicted gene 43109	5.21	0.00
ENSMUSG00000086610	Gm15408	predicted gene 15408	5.22	0.00
ENSMUSG00000030621	Me3	malic enzyme 3	5.23	0.00
ENSMUSG00000047747	Rnf150	ring finger protein 150	5.23	0.00
ENSMUSG00000032375	Aph1b	aph-1 homolog B, gamma-secretase subunit	5.23	0.02
ENSMUSG00000086052	Gm11802	predicted gene 11802	5.24	0.00
ENSMUSG00000033768	Nrxn2	neurexin 2	5.25	0.00
ENSMUSG00000092369	1700039E22Rik	RIKEN cDNA 1700039E22 gene	5.26	0.00
ENSMUSG00000044037	Als2cl	ALS2 C-terminal like	5.27	0.00
ENSMUSG00000120742	Gm56506	predicted gene, 56506	5.28	0.00
ENSMUSG00000031972	Acta1	actin alpha 1, skeletal muscle	5.29	0.00
ENSMUSG00000049349	Gm5105	predicted gene 5105	5.29	0.00
ENSMUSG00000030022	Adamts9	ADAM metallopeptidase with thrombospondin type 1 motif 9	5.30	0.01
ENSMUSG00000085492	Trmt61b	tRNA methyltransferase 61B	5.32	0.00
ENSMUSG00000070601	Vmn2r84	vomeronasal 2, receptor 88	5.33	0.00
ENSMUSG00000030732	Chrdl2	chordin like 2	5.33	0.00
ENSMUSG00000101693	Gm19461	predicted gene, 19461	5.34	0.00
ENSMUSG00000063856	Gpx1	glutathione peroxidase 1	5.34	0.00
ENSMUSG00000073411	H2-D1	major histocompatibility complex, class I, A	5.35	0.00
ENSMUSG00000047656	Trpt1	tRNA phosphotransferase 1	5.38	0.00
ENSMUSG00000021792	Prxl2a	peroxiredoxin like 2A	5.39	0.00
ENSMUSG00000034413	Neurl1b	neuralized E3 ubiquitin protein ligase 1B	5.39	0.00
ENSMUSG00000029866	Kel	Kell metallo-endopeptidase (Kell blood group)	5.39	0.00
ENSMUSG00000093390	Gm20652	predicted gene 20652	5.39	0.00
ENSMUSG00000026879	Gsn	gelsolin	5.40	0.00
ENSMUSG00000008348	Ubc	ubiquitin C	5.40	0.00
ENSMUSG00000120432	Gm56600	predicted gene, 56600	5.40	0.01
ENSMUSG00000032344	Cgas	cyclic GMP-AMP synthase	5.41	0.00
ENSMUSG00000052426	Gm9877	predicted gene 9877	5.41	0.00
ENSMUSG00000103367	Gm38158	predicted gene, 38158	5.41	0.00
ENSMUSG00000070509	Rgma	repulsive guidance molecule BMP co-receptor a	5.42	0.00
ENSMUSG00000087160	Gm16336	predicted gene 16336	5.42	0.00
ENSMUSG00000097756	A730056A06Rik	RIKEN cDNA A730056A06 gene	5.43	0.00
ENSMUSG00000118074	Gm31907	predicted gene, 31907	5.43	0.00
ENSMUSG00000037570	Mcrs1	microspherule protein 1	5.44	0.00
ENSMUSG00000031221	Igbp1	immunoglobulin binding protein 1	5.45	0.03
ENSMUSG00000034968	Lbx2	ladybird homeobox 2	5.46	0.01
ENSMUSG00000022358	Fbxo32	F-box protein 32	5.46	0.00
ENSMUSG00000113619	Gm47118	predicted gene, 47118	5.48	0.00
ENSMUSG00000028128	F3	coagulation factor III, tissue factor	5.48	0.01

Essemble ID	Gene Symbol	Gene Description	Fold Change	P Value
ENSMUSG00000031575	Ash2l	ASH2 like, histone lysine methyltransferase complex subunit	5.48	0.00
ENSMUSG00000089835	Gm7097	predicted gene 7097	5.50	0.00
ENSMUSG00000064280	Ccdc146	coiled-coil domain containing 146	5.51	0.00
ENSMUSG00000054404	Slfn5	schlafen family member 5	5.54	0.00
ENSMUSG00000086507	Adap2os	ArfGAP with dual PH domains 2, opposite strand	5.55	0.00
ENSMUSG00000022756	Slc7a4	solute carrier family 7 member 4	5.58	0.00
ENSMUSG00000006221	Hspb7	heat shock protein family B (small) member 7	5.61	0.00
ENSMUSG00000062638	Btnl1	butyrophilin-like 1	5.61	0.00
ENSMUSG00000085684	4930469K13Rik	RIKEN cDNA 4930469K13 gene	5.65	0.00
ENSMUSG00000093378	Gm20663	predicted gene 20663	5.65	0.00
ENSMUSG00000110457	Gm45752	predicted gene 45752	5.65	0.00
ENSMUSG00000038188	Scarf1	scavenger receptor class F member 1	5.65	0.00
ENSMUSG00000022540	Rogdi	rogdi atypical leucine zipper	5.66	0.00
ENSMUSG00000053769	Lysmd1	LysM domain containing 1	5.68	0.00
ENSMUSG00000085377	4930551L18Rik	RIKEN cDNA 4930551L18 gene	5.69	0.03
ENSMUSG00000047181	Samd14	sterile alpha motif domain containing 14	5.70	0.00
ENSMUSG00000094127	G530012D18Rik	RIKEN cDNA G530012D1 gene	5.71	0.00
ENSMUSG00000017466	Timp2	TIMP metallopeptidase inhibitor 2	5.73	0.00
ENSMUSG00000074491	Clec4g	C-type lectin domain family 4 member G	5.73	0.01
ENSMUSG00000010048	Ifrd2	interferon related developmental regulator 2	5.74	0.00
ENSMUSG00000029309	Sparcl1	SPARC like 1	5.75	0.00
ENSMUSG00000006906	Stambp	STAM binding protein	5.75	0.00
ENSMUSG00000014599	Csf1	colony stimulating factor 1	5.75	0.00
ENSMUSG00000112510	Gm47566	predicted gene, 47566	5.76	0.00
ENSMUSG00000032741	Tpcn1	two pore segment channel 1	5.76	0.00
ENSMUSG00000042121	Ssh1	slingshot protein phosphatase 1	5.77	0.00
ENSMUSG00000040270	Bach2	BTB domain and CNC homolog 2	5.77	0.00
ENSMUSG00000001119	Col6a1	collagen type VI alpha 1 chain	5.77	0.00
ENSMUSG00000034751	Mast4	microtubule associated serine/threonine kinase family member 4	5.78	0.00
ENSMUSG00000039405	Prss23	serine protease 23	5.79	0.00
ENSMUSG00000092517	Art2a	ADP-ribosyltransferase 2a	5.80	0.01
ENSMUSG00000026452	Syt2	synaptotagmin 2	5.81	0.00
ENSMUSG00000091996	Smim35	small integral membrane protein 35	5.82	0.00
ENSMUSG00000073628	Gm10552	predicted gene 10552	5.83	0.00
ENSMUSG00000085890	Tnfsf13os	tumor necrosis factor (ligand) superfamily, member 13, opposite strand	5.83	0.00
ENSMUSG00000028630	Dyrk2	dual specificity tyrosine phosphorylation regulated kinase 2	5.84	0.00
ENSMUSG00000110652	Gm33023	predicted gene, 33023	5.84	0.00
ENSMUSG00000120429	Gm56827	predicted gene, 56827	5.84	0.00
ENSMUSG00000050107	Haspin	histone H3 associated protein kinase	5.86	0.00
ENSMUSG00000111521	Gm48529	predicted gene, 48529	5.88	0.00
ENSMUSG00000030760	Acer3	alkaline ceramidase 3	5.88	0.00
ENSMUSG00000050106	Tmc8	transmembrane channel like 8	5.88	0.00
ENSMUSG00000007891	Ctsd	cathepsin D	5.88	0.00
ENSMUSG00000026959	Grin1	glutamate ionotropic receptor NMDA type subunit 1	5.88	0.00
ENSMUSG00000114329	Gm30489	predicted gene, 30489	5.88	0.00
ENSMUSG00000015943	Bola1	bolA family member 1	5.90	0.00

Essemble ID	Gene Symbol	Gene Description	Fold Change	P Value
ENSMUSG00000029089	Pacrgl	parkin coregulated like	5.92	0.01
ENSMUSG00000037035	Inhbb	inhibin subunit beta B	5.92	0.01
ENSMUSG00000022962	Gart	phosphoribosylglycinamide formyltransferase, phosphoribosylglycinamide synthetase, phosphoribosylaminoimidazole synthetase	5.92	0.00
ENSMUSG00000038721	Hoxb7	homeobox B7	5.92	0.00
ENSMUSG00000116010	Gm36026	predicted gene, 36026	5.93	0.00
ENSMUSG00000030590	Fam98c	family with sequence similarity 98 member C	5.95	0.00
ENSMUSG00000081809	Gm15539	predicted gene 15539	5.95	0.00
ENSMUSG00000038250	Usp38	ubiquitin specific peptidase 38	5.96	0.00
ENSMUSG00000029761	Cald1	caldesmon 1	5.98	0.00
ENSMUSG00000073821	8030451A03Rik	RIKEN cDNA 8030451A03 gene	5.99	0.02
ENSMUSG00000015942	Gtf2ird2	GTF2I repeat domain containing 2	6.00	0.00
ENSMUSG00000031994	Adamts8	ADAM metallopeptidase with thrombospondin type 1 motif 8	6.04	0.00
ENSMUSG00000108079	Gm44210	predicted gene, 44210	6.04	0.00
ENSMUSG00000021070	Bdkrb2	bradykinin receptor B2	6.04	0.02
ENSMUSG00000111673	Gm29724	predicted gene, 29724	6.06	0.00
ENSMUSG00000040451	Sgms1	sphingomyelin synthase 1	6.08	0.00
ENSMUSG00000120665	Gm56761	predicted gene, 56761	6.09	0.00
ENSMUSG00000108436	Gm44851	predicted gene 44851	6.11	0.00
ENSMUSG00000111203	Gm48719	predicted gene, 48719	6.11	0.00
ENSMUSG00000048096	Lmod1	leiomodin 1	6.14	0.00
ENSMUSG00000030739	Myh14	myosin heavy chain 14	6.17	0.03
ENSMUSG00000059713	Rcan3	RCAN family member 3	6.17	0.01
ENSMUSG00000021846	Peli2	pellino E3 ubiquitin protein ligase family member 2	6.19	0.00
ENSMUSG00000026535	Ifi202b	interferon activated gene 202B	6.19	0.00
ENSMUSG00000099590	C330022C24Rik	RIKEN cDNA C330022C24 gene	6.22	0.00
ENSMUSG00000108126	Gm43909	predicted gene, 43909	6.22	0.00
ENSMUSG00000047098	Rnf31	ring finger protein 31	6.25	0.00
ENSMUSG00000111535	Gm35154	predicted gene, 35154	6.26	0.00
ENSMUSG00000101925	Gm28156	predicted gene 28156	6.26	0.00
ENSMUSG00000031750	Il34	interleukin 34	6.29	0.00
ENSMUSG00000006920	Ezh1	enhancer of zeste 1 polycomb repressive complex 2 subunit	6.29	0.00
ENSMUSG00000027811	4930579G24Rik	chromosome 4 open reading frame 46	6.29	0.00
ENSMUSG00000028464	Tpm2	tropomyosin 2, beta	6.29	0.00
ENSMUSG00000048489	Depp1	DEPP autophagy regulator 1	6.31	0.00
ENSMUSG00000105746	Gm43595	predicted gene 43595	6.31	0.00
ENSMUSG00000029161	Cgref1	cell growth regulator with EF-hand domain 1	6.33	0.00
ENSMUSG00000116030	Gm49500	predicted gene, 49500	6.38	0.00
ENSMUSG00000043903	Zfp469	zinc finger protein 469	6.38	0.00
ENSMUSG00000040829	Zmynd15	zinc finger MYND-type containing 15	6.40	0.00
ENSMUSG00000001870	Ltbp1	latent transforming growth factor beta binding protein 1	6.42	0.00
ENSMUSG00000034958	Atcay	ATCAY kinesin light chain interacting caytaxin	6.44	0.00
ENSMUSG00000121112	Gm57055	predicted gene, 49528	6.49	0.00
ENSMUSG00000116116	Gm671	predicted gene 671	6.51	0.00
ENSMUSG00000039633	Lonrf1	LON peptidase N-terminal domain and ring finger 1	6.52	0.01
ENSMUSG00000111797	Gm48081	predicted gene, 48081	6.55	0.00

Essemble ID	Gene Symbol	Gene Description	Fold Change	P Value
ENSMUSG00000109491	Gm15396	predicted gene 15396	6.55	0.00
ENSMUSG00000116439	Gm49528	predicted gene, 49528	6.56	0.00
ENSMUSG00000057342	Sphk2	sphingosine kinase 2	6.58	0.00
ENSMUSG00000026729	4930562F07Rik	RIKEN cDNA 4930562F07 gene	6.59	0.00
ENSMUSG00000068463	B630019A10Rik	RIKEN cDNA B630019A10 gene	6.59	0.00
ENSMUSG00000050663	Trhde	thyrotropin releasing hormone degrading enzyme	6.59	0.00
ENSMUSG00000118320	Gm50425	predicted gene, 50425	6.59	0.00
ENSMUSG00000120996	Gm56817	predicted gene, 56817	6.60	0.00
ENSMUSG00000076939	Iglv3	immunoglobulin lambda variable 3	6.61	0.00
ENSMUSG00000097871	B230104I21Rik	RIKEN cDNA B230104I21 gene	6.63	0.00
ENSMUSG00000010311	Optc	opticin	6.65	0.00
ENSMUSG00000074207	Adh1	alcohol dehydrogenase 1C (class I), gamma polypeptide	6.65	0.00
ENSMUSG00000025040	Fundc1	FUN14 domain containing 1	6.66	0.00
ENSMUSG00000067818	Myl9	myosin light chain 9	6.67	0.00
ENSMUSG00000104350	Gm38244	predicted gene, 38244	6.69	0.00
ENSMUSG00000029174	Tbc1d1	TBC1 domain family member 1	6.70	0.00
ENSMUSG00000020739	Nup85	nucleoporin 85	6.71	0.00
ENSMUSG00000120173	Gm56774	predicted gene, 56774	6.72	0.00
ENSMUSG00000115026	Gm49041	predicted gene, 49041	6.72	0.01
ENSMUSG00000051586	Mical3	microtubule associated monooxygenase, calponin and LIM domain containing 3	6.73	0.00
ENSMUSG00000020876	Snx11	sorting nexin 11	6.74	0.00
ENSMUSG00000003378	Grik5	glutamate ionotropic receptor kainate type subunit 5	6.76	0.00
ENSMUSG00000043541	Dnai7	dynein axonemal intermediate chain 7	6.78	0.00
ENSMUSG00000097157	Gm26512	predicted gene, 26512	6.80	0.00
ENSMUSG00000037379	Spon2	spondin 2	6.83	0.00
ENSMUSG00000001349	Cnn1	calponin 1	6.88	0.00
ENSMUSG00000053411	Cbx7	chromobox 7	6.88	0.00
ENSMUSG00000116220	A430088P11Rik	RIKEN cDNA A430088P11 gene	6.90	0.00
ENSMUSG00000038370	Pcp4l1	Purkinje cell protein 4-like 1	6.90	0.00
ENSMUSG00000037820	Tgm2	transglutaminase 2	6.91	0.00
ENSMUSG00000104159	Gm38099	predicted gene, 38099	6.93	0.00
ENSMUSG00000000982	Ccl3	C-C motif chemokine ligand 3 like 3	6.94	0.00
ENSMUSG00000034164	Emid1	EMI domain containing 1	6.95	0.00
ENSMUSG00000058756	Thra	thyroid hormone receptor alpha	6.96	0.00
ENSMUSG00000026126	Ptpn18	protein tyrosine phosphatase non-receptor type 18	6.97	0.00
ENSMUSG00000061410	Zcchc14	zinc finger CCHC-type containing 14	6.99	0.00
ENSMUSG00000024732	Ccdc86	coiled-coil domain containing 86	7.02	0.00
ENSMUSG00000000184	Ccnd2	cyclin D2	7.02	0.00
ENSMUSG00000020718	Polg2	DNA polymerase gamma 2, accessory subunit	7.04	0.00
ENSMUSG00000086791	Gm12147	predicted gene 12147	7.07	0.00
ENSMUSG00000041889	Shisa4	shisa family member 4	7.11	0.00
ENSMUSG00000086567	Gm2830	predicted gene 2830	7.11	0.00
ENSMUSG00000052336	Cx3cr1	C-X3-C motif chemokine receptor 1	7.13	0.00
ENSMUSG00000112734	Gm47644	predicted gene, 47644	7.15	0.00
ENSMUSG00000107078	Gm36840	predicted gene, 36840	7.18	0.00
ENSMUSG00000114361	Gm31104	predicted gene, 31104	7.19	0.01

Essemble ID	Gene Symbol	Gene Description	Fold Change	P Value
ENSMUSG00000086656	Gm15701	predicted gene 15701	7.21	0.00
ENSMUSG00000009681	Bcr	BCR activator of RhoGEF and GTPase	7.22	0.00
ENSMUSG00000048502	Duxbl1	double homeobox B-like 1	7.30	0.00
ENSMUSG00000074575	Kcng1	potassium voltage-gated channel modifier subfamily G member 1	7.32	0.00
ENSMUSG00000106709	Gm30270	predicted gene, 30270	7.32	0.00
ENSMUSG00000025076	Casp7	caspase 7	7.33	0.00
ENSMUSG00000103357	Gm7804	ribosomal protein SA pseudogene	7.34	0.00
ENSMUSG00000008845	Cd163	CD163 molecule	7.38	0.00
ENSMUSG00000044573	Acp1	acid phosphatase 1	7.40	0.00
ENSMUSG00000079419	Ms4a6c	membrane-spanning 4-domains, subfamily A, member 6C	7.41	0.00
ENSMUSG00000005580	Adcy9	adenylate cyclase 9	7.41	0.00
ENSMUSG00000027698	Nceh1	neutral cholesterol ester hydrolase 1	7.42	0.01
ENSMUSG00000028989	Angptl7	angiopoietin like 7	7.45	0.01
ENSMUSG00000052353	Cemip	cell migration inducing hyaluronidase 1	7.45	0.00
ENSMUSG00000077222	Gm22270	predicted gene, 22270	7.48	0.00
ENSMUSG00000105900	Gm43240	predicted gene 43240	7.48	0.00
ENSMUSG00000000690	Hoxb6	homeobox B6	7.54	0.00
ENSMUSG00000069516	Lyz2	lysozyme	7.54	0.00
ENSMUSG00000093722	Gm20612	predicted gene 20612	7.55	0.00
ENSMUSG00000075702	Selenom	selenoprotein M	7.59	0.00
ENSMUSG00000049112	Oxtr	oxytocin receptor	7.65	0.00
ENSMUSG00000034842	Art3	ADP-ribosyltransferase 3 (inactive)	7.68	0.00
ENSMUSG00000074737	C530025M09Rik	RIKEN cDNA C530025M09 gene	7.69	0.00
ENSMUSG00000120977	Gm32164	predicted gene, 32164	7.74	0.00
ENSMUSG00000035469	Rcbtb1	RCC1 and BTB domain containing protein 1	7.76	0.00
ENSMUSG00000089671	Gm16537	predicted gene 16537	7.76	0.00
ENSMUSG00000038700	Hoxb5	homeobox B5	7.76	0.00
ENSMUSG00000000120	Ngfr	nerve growth factor receptor	7.77	0.00
ENSMUSG00000101029	Gm29439	predicted gene 29439	7.78	0.00
ENSMUSG00000120878	Gm56663	predicted gene, 56663	7.78	0.00
ENSMUSG00000116707	Gm49590	predicted gene, 49590	7.79	0.02
ENSMUSG00000022505	Emp2	epithelial membrane protein 2	7.79	0.00
ENSMUSG00000024164	C3	complement C3	7.80	0.00
ENSMUSG00000121008	Gm56974	predicted gene, 56974	7.89	0.01
ENSMUSG00000026579	F5	coagulation factor V	7.90	0.00
ENSMUSG00000086170	Gm12144	predicted gene 12144	7.92	0.00
ENSMUSG00000098098	Bvht	braveheart long non-coding RNA	7.99	0.00
ENSMUSG00000060188	Cxcl17	C-X-C motif chemokine ligand 17	8.03	0.02
ENSMUSG00000018830	Myh11	myosin heavy chain 11	8.07	0.00
ENSMUSG00000085516	4930538E20Rik	RIKEN cDNA 4930538E20 gene	8.08	0.00
ENSMUSG00000092243	H2-T25	major histocompatibility complex, class I, F	8.09	0.00
ENSMUSG00000026361	Cdc73	cell division cycle 73	8.10	0.00
ENSMUSG00000094868	Dlx6os2	distal-less homeobox 6, opposite strand 2	8.15	0.00
ENSMUSG00000104077	Gm37027	predicted gene, 37027	8.15	0.00
ENSMUSG00000035783	Acta2	actin alpha 2, smooth muscle	8.16	0.00
ENSMUSG00000030786	Itgam	integrin subunit alpha M	8.18	0.00

Essemble ID	Gene Symbol	Gene Description	Fold Change	P Value
ENSMUSG00000120733	Gm56657	predicted gene, 56657	8.19	0.00
ENSMUSG00000097233	Gm17552	predicted gene, 17552	8.21	0.00
ENSMUSG00000026415	Fcamr	Fc alpha and mu receptor	8.28	0.00
ENSMUSG00000095079	Igha	immunoglobulin heavy constant alpha	8.29	0.01
ENSMUSG00000048065	Cyb5r2	cytochrome b5 reductase 2	8.35	0.00
ENSMUSG00000065389	Gm23100	predicted gene, 23100	8.37	0.00
ENSMUSG00000026429	Ube2t	ubiquitin conjugating enzyme E2 T	8.38	0.01
ENSMUSG00000028036	Ptgfr	prostaglandin F receptor	8.40	0.00
ENSMUSG00000019880	Rspo3	R-spondin 3	8.46	0.00
ENSMUSG00000021200	Asb2	ankyrin repeat and SOCS box containing 2	8.51	0.00
ENSMUSG00000111854	Gm48740	predicted gene, 48740	8.51	0.00
ENSMUSG00000017756	Slc12a7	solute carrier family 12 member 7	8.58	0.00
ENSMUSG00000073633	Fbxo36	F-box protein 36	8.61	0.02
ENSMUSG00000097108	Gm26581	predicted gene, 26581	8.63	0.00
ENSMUSG00000120679	Gm33799	predicted gene, 33799	8.64	0.00
ENSMUSG00000113636	Gm8616	ribonucleotide reductase M2 B (TP53 inducible) pseudogene	8.65	0.00
ENSMUSG00000085533	Gm12146	predicted gene 12146	8.67	0.00
ENSMUSG00000100600	A230077H06Rik	RIKEN cDNA A230077H06 gene	8.70	0.00
ENSMUSG00000085944	1700003D09Rik	RIKEN cDNA 1700003D09 gene	8.72	0.00
ENSMUSG00000084890	A830036E02Rik	RIKEN cDNA A830036E02 gene	8.74	0.00
ENSMUSG00000042514	Klhl14	kelch like family member 14	8.82	0.00
ENSMUSG00000040424	Hipk4	homeodomain interacting protein kinase 4	8.84	0.00
ENSMUSG00000036899	Trpv5	transient receptor potential cation channel subfamily V member 5	8.93	0.00
ENSMUSG00000050765	Gm5084	predicted gene 5084	8.94	0.00
ENSMUSG00000108530	4930429H19Rik	RIKEN cDNA 4930429H19 gene	8.95	0.00
ENSMUSG00000015501	Hivep2	HIVEP zinc finger 2	8.97	0.00
ENSMUSG00000039899	Fgl2	fibrinogen like 2	9.00	0.00
ENSMUSG00000037852	Cpe	carboxypeptidase E	9.03	0.00
ENSMUSG00000038594	Cep85l	centrosomal protein 85 like	9.10	0.00
ENSMUSG00000077426	Gm26387	predicted gene, 26387	9.21	0.00
ENSMUSG00000052446	Zfp961	zinc finger protein 961	9.25	0.00
ENSMUSG00000034156	Tspoap1	TSPO associated protein 1	9.28	0.00
ENSMUSG00000004939	Nmrk2	nicotinamide riboside kinase 2	9.34	0.00
ENSMUSG00000068740	Celsr2	cadherin EGF LAG seven-pass G-type receptor 2	9.35	0.00
ENSMUSG00000090877	Hspa1b	heat shock protein 1B	9.44	0.00
ENSMUSG00000103434	Gm36993	predicted gene, 36993	9.48	0.00
ENSMUSG00000064215	Ifi27	interferon, alpha-inducible protein 27	9.55	0.00
ENSMUSG00000021379	Id4	inhibitor of DNA binding 4	9.55	0.00
ENSMUSG00000085971	Gm15411	predicted gene 15411	9.57	0.00
ENSMUSG00000017760	Ctsa	cathepsin A	9.67	0.00
ENSMUSG00000098197	BC051537	cDNA sequence BC051537	9.72	0.00
ENSMUSG00000051076	Vtcn1	V-set domain containing T cell activation inhibitor 1	9.73	0.00
ENSMUSG00000086381	AV064505	expressed sequence AV064505	9.75	0.01
ENSMUSG00000025969	Nrp2	neuropilin 2	9.84	0.00
ENSMUSG00000047139	Cd24a	CD24a antigen	10.00	0.00
ENSMUSG00000111796	Gm47575	predicted gene, 47575	10.03	0.00

Essemble ID	Gene Symbol	Gene Description	Fold Change	P Value
ENSMUSG00000089697	Gm15947	predicted gene 15947	10.03	0.00
ENSMUSG00000117007	Gm41556	predicted gene, 41556	10.06	0.00
ENSMUSG00000073421	H2-Ab1	major histocompatibility complex, class II, DQ beta 1	10.09	0.00
ENSMUSG00000081611	Gm13651	phosphoribosyl pyrophosphate synthetase-associated protein 2 pseudogene	10.13	0.00
ENSMUSG00000047330	Kcne4	potassium voltage-gated channel subfamily E regulatory subunit 4	10.23	0.00
ENSMUSG00000063873	Slc24a3	solute carrier family 24 member 3	10.30	0.00
ENSMUSG00000039126	Prune2	prune homolog 2 with BCH domain	10.36	0.00
ENSMUSG00000002985	Apoe	apolipoprotein E	10.41	0.00
ENSMUSG00000025329	Padi1	peptidyl arginine deiminase 1	10.51	0.01
ENSMUSG00000099757	BE692007	expressed sequence BE692007	10.59	0.00
ENSMUSG00000107075	Gm43068	predicted gene 43068	10.60	0.00
ENSMUSG00000028963	Uts2	urotensin 2	10.64	0.00
ENSMUSG00000114871	Gm21370	predicted gene, 21370	10.65	0.00
ENSMUSG00000080715	Gm5406	proteasome (prosome, macropain) subunit, alpha type 3 pseudogene	10.69	0.00
ENSMUSG00000118361	Gm50237	predicted gene, 50237	10.72	0.00
ENSMUSG00000106567	2010309G21Rik	RIKEN cDNA 2010309G21 gene	10.75	0.00
ENSMUSG00000045078	Rnf216	ring finger protein 216	11.03	0.00
ENSMUSG00000091060	Myocos	myocilin opposite strand	11.03	0.00
ENSMUSG00000108484	Gm18537	DENN/MADD domain containing 4C pseudogene	11.07	0.00
ENSMUSG00000092471	Cyp21a2-ps	cytochrome P450, family 21, subfamily a, polypeptide 2 pseudogene	11.08	0.00
ENSMUSG00000112023	Lilrb4b	leukocyte immunoglobulin like receptor B4	11.11	0.00
ENSMUSG00000016493	Cd46	CD46 molecule	11.22	0.00
ENSMUSG00000086801	Gm15943	predicted gene 15943	11.28	0.00
ENSMUSG00000073980	Gm9574	histocompatibility 2, T region pseudogene	11.30	0.00
ENSMUSG00000047730	Fcgbp	Fc gamma binding protein	11.38	0.01
ENSMUSG00000087002	Gm16277	predicted gene 16277	11.45	0.00
ENSMUSG00000021779	Thrb	thyroid hormone receptor beta	11.48	0.00
ENSMUSG00000114618	Gm41183	predicted gene, 41183	11.56	0.00
ENSMUSG00000079055	Slc8a3	solute carrier family 8 member A3	11.61	0.00
ENSMUSG00000025068	Gsto1	glutathione S-transferase omega 1	11.66	0.00
ENSMUSG00000114375	Gm31544	predicted gene, 31544	11.67	0.00
ENSMUSG00000026417	Pigr	polymeric immunoglobulin receptor	11.70	0.00
ENSMUSG00000085089	Arhgap15os	Rho GTPase activating protein 15, opposite strand	11.76	0.00
ENSMUSG00000028458	Tesk1	testis associated actin remodelling kinase 1	11.96	0.00
ENSMUSG00000098144	1700029N11Rik	RIKEN cDNA 1700029N11 gene	12.03	0.00
ENSMUSG00000090066	1110002E22Rik	chromosome 4 open reading frame 54	12.12	0.00
ENSMUSG00000076609	Igkc	immunoglobulin kappa constant	12.15	0.00
ENSMUSG00000021578	Ccdc127	coiled-coil domain containing 127	12.21	0.00
ENSMUSG00000036887	C1qa	complement C1q A chain	12.41	0.00
ENSMUSG00000031489	Adrb3	adrenoceptor beta 3	12.41	0.00
ENSMUSG00000089635	Gm16559	predicted gene 16559	12.49	0.00
ENSMUSG00000067149	Jchain	joining chain of multimeric IgA and IgM	12.66	0.00
ENSMUSG00000066235	Pomgnt2	protein O-linked mannose N-acetylglucosaminyltransferase 2 (beta 1,4-)	12.68	0.00
ENSMUSG00000050967	Creg2	cellular repressor of E1A stimulated genes 2	12.71	0.00
ENSMUSG00000072109	A530040E14Rik	RIKEN cDNA A530040E14 gene	12.79	0.00
ENSMUSG00000041577	Prelp	proline and arginine rich end leucine rich repeat protein	12.80	0.00

Essemble ID	Gene Symbol	Gene Description	Fold Change	P Value
ENSMUSG00000030737	Slco2b1	solute carrier organic anion transporter family member 2B1	12.82	0.00
ENSMUSG00000021567	Nkd2	NKD inhibitor of WNT signaling pathway 2	12.94	0.00
ENSMUSG00000017817	Jph2	junctophilin 2	12.98	0.00
ENSMUSG00000020788	Atp2a3	ATPase sarcoplasmic/endoplasmic reticulum Ca2+ transporting 3	13.03	0.00
ENSMUSG00000029304	Spp1	secreted phosphoprotein 1	13.03	0.01
ENSMUSG00000037166	Ppp1r14a	protein phosphatase 1 regulatory inhibitor subunit 14A	13.04	0.00
ENSMUSG00000110520	Gm45776	predicted gene 45776	13.31	0.03
ENSMUSG00000070034	Sp110	SP110 nuclear body protein	13.40	0.00
ENSMUSG00000034764	1700006J14Rik	RIKEN cDNA 1700006J14 gene	13.46	0.00
ENSMUSG00000004266	Ptpn6	protein tyrosine phosphatase non-receptor type 6	13.48	0.00
ENSMUSG00000031844	Hsd17b2	hydroxysteroid 17-beta dehydrogenase 2	13.53	0.01
ENSMUSG00000036896	C1qc	complement C1q C chain	13.68	0.00
ENSMUSG00000038055	Dexi	Dexi homolog	13.80	0.00
ENSMUSG00000113159	Gm48771	predicted gene, 48771	13.84	0.00
ENSMUSG00000023336	Wfdc1	WAP four-disulfide core domain 1	14.03	0.00
ENSMUSG00000106093	Gm42722	predicted gene 42722	14.61	0.03
ENSMUSG00000042854	Trp53rkb	TP53 regulating kinase	15.07	0.00
ENSMUSG00000049382	Krt8	keratin 8	15.24	0.00
ENSMUSG00000112397	Gm10824	predicted gene 10824	15.70	0.00
ENSMUSG00000050742	Or2m12	olfactory receptor family 2 subfamily M member 3	16.06	0.00
ENSMUSG00000102155	Gm37468	predicted gene, 37468	16.07	0.00
ENSMUSG00000024610	Cd74	CD74 molecule	16.13	0.00
ENSMUSG00000061353	Cxcl12	C-X-C motif chemokine ligand 12	16.13	0.00
ENSMUSG00000026574	Dpt	dermatopontin	16.38	0.00
ENSMUSG00000087477	Gm13822	predicted gene 13822	16.45	0.00
ENSMUSG00000090185	Gm15523	predicted gene 15523	16.60	0.00
ENSMUSG00000111116	Gm48065	predicted gene, 48065	16.74	0.00
ENSMUSG00000032572	Col6a4	collagen, type VI, alpha 4	16.81	0.00
ENSMUSG00000101493	2810405F17Rik	RIKEN cDNA 2810405F17 gene	17.20	0.00
ENSMUSG00000085872	Gm11505	predicted gene 11505	17.63	0.00
ENSMUSG00000025784	Clec3b	C-type lectin domain family 3 member B	18.01	0.00
ENSMUSG00000050195	Scd4	stearoyl-coenzyme A desaturase 4	18.03	0.01
ENSMUSG00000108778	Gm20083	predicted gene, 20083	20.04	0.00
ENSMUSG00000053519	Kcnip1	potassium voltage-gated channel interacting protein 1	20.74	0.00
ENSMUSG00000055452	Gm7353	zinc finger CCCH type containing 3 pseudogene	20.78	0.00
ENSMUSG00000052477	Sp140l2	Sp140 nuclear body protein like 2	20.91	0.00
ENSMUSG00000054619	Tmt1a	thiol methyltransferase 1A	21.89	0.00
ENSMUSG00000092200	Tnxa	tenascin XA (pseudogene)	22.07	0.00
ENSMUSG00000085213	Gm13091	predicted gene 13091	22.31	0.00
ENSMUSG00002075844	Gm55157	predicted gene, 55157	22.53	0.01
ENSMUSG00000102778	Gm38165	predicted gene, 38165	22.61	0.00
ENSMUSG00000085918	Gm13032	predicted gene 13032	24.68	0.00
ENSMUSG00000018012	Rac3	Rac family small GTPase 3	26.65	0.00
ENSMUSG00000014453	Blk	BLK proto-oncogene, Src family tyrosine kinase	27.87	0.00
ENSMUSG00000101024	Gm5692	DEAD (Asp-Glu-Ala-Asp) box polypeptide 18 pseudogene	28.10	0.00
ENSMUSG00000120512	5730409N16Rik	RIKEN cDNA 5730409N16 gene	28.72	0.00

Essemble ID	Gene Symbol	Gene Description	Fold Change	P Value
ENSMUSG00000036813	Entpd8	ectonucleoside triphosphate diphosphohydrolase 8	29.14	0.00
ENSMUSG00000076613	Ighg2b	immunoglobulin heavy constant gamma 2B	29.21	0.00
ENSMUSG00000035638	Muc20	mucin 20, cell surface associated	29.58	0.00
ENSMUSG00000032068	Plet1	placenta expressed transcript 1	30.03	0.00
ENSMUSG00000101304	Plet1os	placenta expressed transcript 1, opposite strand	30.21	0.00
ENSMUSG00000086868	Gm15883	predicted gene 15883	32.15	0.00
ENSMUSG00000098424	Gm27202	predicted gene 27202	33.15	0.00
ENSMUSG00000073631	Gm10553	predicted gene 10553	34.40	0.00
ENSMUSG00000030717	Nupr1	nuclear protein 1, transcriptional regulator	37.36	0.00
ENSMUSG00000026822	Lcn2	lipocalin 2	40.86	0.00
ENSMUSG00000107523	Gm44451	predicted gene 44451	44.08	0.00
ENSMUSG00000112682	Gm48874	predicted gene 48874	49.00	0.00
ENSMUSG00000003477	Inmt	indolethylamine N-methyltransferase	56.56	0.00
ENSMUSG00000076612	Ighg2c	immunoglobulin heavy constant gamma 2C	65.60	0.00
ENSMUSG00000104111	Gm37294	predicted gene, 37294	72.43	0.00
ENSMUSG00000050635	Sprr2f	small proline-rich protein 2F	79.18	0.00
ENSMUSG00000109501	Gm14377	predicted gene 14377	105.97	0.00
ENSMUSG00000089851	Gm16579	predicted gene 16579	119.19	0.00
ENSMUSG00000025150	Cbr2	carbonyl reductase 2	131.41	0.00
ENSMUSG00000032496	Ltf	lactotransferrin	191.63	0.00
ENSMUSG00002076431	Gm55161	predicted gene, 55161	213.24	0.00
